# Acknowledgement to Reviewers of *Molecules* in 2019

**DOI:** 10.3390/molecules25020427

**Published:** 2020-01-20

**Authors:** Molecules Editorial Office

**Affiliations:** MDPI, St. Alban-Anlage 66, 4052 Basel, Switzerland

The editorial team greatly appreciates the reviewers who have dedicated their considerable time and expertise to the journal’s rigorous editorial process over the past 12 months, regardless of whether the papers are finally published or not. In 2019, a total of 4619 papers were published in the journal, with a median time to first decision of 13 days and a median time from submission to publication of 32 days. The editors would like to express their sincere gratitude to the following reviewers for their generous contribution in 2019:
A. Elbarbry, FawzyLönnberg, HarriAbakumov, E. V.Lönnberg, TuomasAbarbri, MohamedLopes, GracilianaAbashev, George G.Lopes, Pedro E. M.Abbate, SergioLopez Rodilla, Jesus M.Abbina, SrinivasLópez, ConcepciónAbbott, AndrewLopez, Jose CristobalAbd El Hakim, YasminaLópez, Susana B BravoAbdalla, Maher Y.López-Cepero, María RocaAbd-ElGawad, AhmedLópez-Cornejo, PilarAbdellatif, AhmedLópez-Ferber, MiguelAbdel-Latif, EhabLópez-Linares, Juan CarlosAbdelmohsen, Usama RamadanLopez-Molina, CarlosAbdelwahed, SamehLópez-Padilla, AlexisAbdi Jalebi, MojtabaLópez-Ramón, Maria VictoriaAbdollahi, MehdiLópez-Salas, NievesAbeywardana, TharindumalaLoppi, SteffanoAbhijit, SahaLorberboum-Galski, HayaAbonia, RodrigoLorenzi, VanninaAboudi, KaoutarLorenzo, Jose M.Ábrányi-Balogh, PéterLorenzo-Morales, JacobAbuillan, WasimLosch, PitAbuin, GracielaLostao, María PilarAbu-Reidah, Ibrahim M.Losurdo, GiuseppeAbushammala, HatemLotina-Hennsen, BlasAccardo, GraziaLou, ChenguangAcedo, Jeella Z.Lou, HongxiangAcero, NuriaLouarn, EssylltAcha, VictorLoureiro, Felipe Dos SantosAchilias, Dimitris S.Lourenço, LeandroAchim, CristinaLovell, JonathanAchour, BrahimLowin, TorstenAčkar, ĐurđicaLoza-Mejía, Marco A.Acosta Contreras, Florentina NiurisLoza-Mejía, Marco AntonioAcree Jr., William E.Lozano-Ojalvo, DanielÁcs, KamillaLu, DongdongAdachi, SadaoLu, JingzhiAdamczak, MarekLu, Jyh-FengAdamczyk, AgataLu, Mei-ChinAdamczyk-Woźniak, AgnieszkaLu, QuanlongAdamek, JakubLu, ShaoyongAdams, JamesLu, ShuiyuAdams, James DavidLu, SizhaoAdamski, ZbigniewLu, WeiyueAdan, RogerLu, XiaoAddepalli, BalasubrahmanyamLu, YiAdebo, OluwafemiLu, YouAdeleye, Adeyemi S.Lubawy, JanAdhikari, Birendra B.Lubin-Germain, NadègeAdhikary, AmitavaLuca, OanaAdihou, HeleneLucarini, MassimoAdil, Syed FarooqLucarini, SimoneAdjaoud, OmarLucas, Jose AntonioAdly, Frady G.Luccini, LuigiAdonin, Sergey A.Lucena, RafaelAdunyah, Samuel E.Lucenti, ElenaAflori, MagdalenaLuchinat, ClaudioAfonin, Andrei V.Lucioli, SimonaAfonso, Carlos M.Ludwiczuk, AgnieszkaAfseth, Nils KristianLuévano-Contreras, ClaudiaAgarwal, VivechanaLugli, FedericoAgback, PeterLui, Edmund Man KingAgeitos, Jose ManuelLui, Vincent Chi-HangAggeli, Ioanna-KaterinaLuin, StefanoAgnieszka, SzopaLukáč, MilošAgòcs, AttilaLukas, JanAgrimi, GennaroLukasik, RafalAguayo, Maria GracielaLukaszewski, MariuszÁgueda, Vicente I.Lukic, IgorAguilar-Caballos, María PazLukova, PaolinaAguiló-Aguayo, NoemíŁukowiec, DariuszAguirre, Nestor F.Łukowski, PawełAguirre-Crespo, Francisco J.Lukšič, MihaAguirre-Soto, AlanLukyanov, PavelAgustin, DominiqueLuliński, SergiuszAgut, MontserratLung, Ming-YeouAhern, Gerard P.Lung, Shiu CheungAhmad, AamirLunov, OlegAhmad, AliLuo, LinAhmad, AmirLuo, RongcongAhmad, ShakilLuo, WentaiAhmad, ZulfiqarLuo, XianAhmadivand, ArashLuo, ZishengAhmed, Adel A.Lupu, StelianAhmed, MaryaLuque, AmaliaAhmed, Mohammad BoshirLuque, F. JavierAhmed, SaharLusczek, Elizabeth R.Ahn, Ji-YoungLushington, GerryAhvazi, BehzadLutz, MarianeAhvenainen, PatrikLutz, MartinAi, YongLuz, Rita De Cássia SilvaAich, NirupamLuzina, OlgaAiello, FrancescaLymperopoulou, TheopistiAiello, GildaŁyszczek, RenataAinsworth, Richard I.M Gómez, ClaraAiroudj, AissamM. Vorob’ev, MikhailAisa, Haji AkberMa, BuyongAitipamula, SrinivasuluMa, Dik-LungAitken, R. AlanMa, FuyingAkabayov, BarakMa, JieAkbar, MohammedMa, LongAkens, MargareteMa, SongqiAkhtar, SultanMa, TianyiAkingbade, Katherine L.Ma, WanhongAkitaya, TatsuoMa, XiaoliAkitsu, TakashiroMa, YuanAkopova, TatianaMa, ZhikunAksel, HacerMaász, GáborAlaibac, MauroMaayah, Zaid H.Alaimo, AlessandroMaayan, GaliaAlam, Md BadrulMabou Tagne, AlexAlam, Md KausarMacák, JanAlam, Mohammad AbrarMacauley, Matthew S.Al-Anssari, SarmadMaccallini, CristinaAlarif, Walied MohamedMacchi, BeatriceAlbalat, AmayaMacchi, PieroAlbarrán, GuadalupeMaccioni, EliasAlbericio, FernandoMacel, MirkaAlbonici, LoredanaMacheret, SergeyAlbouy, Pierre-AntoineMacia, AlbaAlbuquerque, HélioMacías, RamónAlbuquerque, MagalyMacias-Gonzalez, ManuelAlcantara, AndresMaciążek-Jurczyk, MałgorzataAlcarde, Andre RicardoMaciejczyk, MateuszAlcaro, StefanoMackenzie, SallyAlcolea, VeronicaMackereth, CameronAlcoutlabi, MatazMacone, AlbertoAldabbagh, FawazMączka, MirosławAldred, KatieMączka, WandaAlduina, RosaMączyński, MarcinAlejandro-Martin, SergueiMaddelein, Marie-LiseAlekseeva, AnnaMadder, AnnemiekeAlemán, CarlosMadhukar, BurraAleu, JosefinaMadine, JillianAlexa, ErsiliaMadrid, AlejandroAlexiev, UlrikeMadsen, RobertAlexopoulos, AthanassiosMady, Mohamed F.Alexovič, MichalMadzhidov, TimurAl-Fatimi, MohamedMaeda, HayatoAlfinito, EleonoraMaeda, HiromitsuAlguacil, FranciscoMaeda, KazuyaAl-Horani, Rami A.Maeda, NobuoAli, Hayssam M.Maeda, TomokiAli, RameezMaekawa, MasamitsuAlikhani, EsmailMaeng, Han-JooAl-Isawi, Rawaa H KMaestrelli, FrancescaAljabali, Alaa A AMaestri, ElenaAllaf, KarimMaffei, GianlucaAllain, ClémenceMafra, IsabelAllman, Sarah AnnMafra, Marcos RogérioAl-Maharik, NawafMafu, SibongileAlmajano, María PilarMaga, GiovanniAlmansa, María SoledadMagalhães, Júlia M.C.S.Al-Masum, MohammadMagalhaes, Perola OliveiraAlmeida Júnior, José HumbertoMagaña, Luis FernandoAlmendros, GonzaloMagán-Fernández, AntonioAlmiro de Almeida, AgostinoMagdanz, VeronikaAlmoazen, HassanMaGee, DavidAlmogi-Hazan, OsnatMaggi, FilippoAl-Mrabeh, AhmadMaggi, Leonard B.Aloia, LuigiMaggiolini, MarcelloAlonso, Diego A.Magiorkinis, EmmanouilAlonso, Rosa MariaMagnacca, GiulianaAlpini, GianfrancoMagoulas, GeorgeAl-Rimawi, FuadMagriotis, Plato A.Alshannaq, AhmadMaher, SimonAlt, HelmutMahmood, KhalidAltemimi, AMMAR.BMahmood, NasirAltieri, FabioMahmoudi, FarzanehAltmann, FriedrichMahomoodal, FawziAltomare, ClaudiaMahy, Julien G.Alugubelly, NavathaMaiale, Santiago J.Alvarez Fernandez, Maria AntoniaMaicas, SergiAlvarez, AlejandraMaier, CameliaÁlvarez, EleuterioMaier, MartinAlvarez, GuzmánMaietti, AnnalisaAlvarez, Hugo ArielMainero Rocca, LuciaAlvarez, LauraMaione, FrancescoÁlvarez, María S.Maixent, Jean MichelAlvarez, Miriam PenaMajcher, Malgorzata A.Alvarez-Idaboy, Juan RaùlMajinda, Runner R.T.Alvarez-Suarez, JoseMajireck, MaxÁlvarez-Suarez, José M.Majkowska-Pilip, AgnieszkaAlves, ElianaMajkowska-Skrobek, GrażynaAlves, FranciscoMajolino, DomenicoAlves, MarcoMajoros, LaszloAlves, Vítor H.Majumdar, SusrutaAlves-Silva, Jorge MMakabe, HidehumiAlvira, ElenaMakal, TrevorAlZubaidi, Abbas K.Makarska-Bialokoz, MagdalenaAmachi, SeigoMakena, Monish RamAmante, Edna ReginaMaker, GarthAmariei, SoniaMaki, YasuyukiAmarowicz, RyszardMäki-Arvela, PäiviAmata, EmanueleMäkinen, SariAmato, FeliceMakisha, NikolayAmato, JussaraMakishima, MakotoAmbro, LubosMakris, Dimitris P.Amenabar, MaximilianoMakunga, Nokwanda P.Amer, HassanMalaguarnera, MicheleAmérica R., Vázquez-OlmosMalandrino, MeryAmes, James BMalavasi, GianlucaAmi, DilettaMalawska, BarbaraAmigo, LourdesMaldonado Hodar, Francisco JoséAmii, HidekiMalegori, CristinaAmillis, SotirisMalik, Muhammad ImranAmini, ShahrouzMalinge, Jan-MarcAmodio, NicolaMalinowska, MagdalenaAmoroso, SalvatoreMalinowski, SzymonAmovilli, ClaudioMallipeddi, Prema LathaAmsharov, KonstantinMallipeddi, SrikrishnanAn, ZhongfuMalmberg, PerAna Perez, RosaMalmström, LarsAna, Juan-GarcíaMaloberti, AlessandroAnadón, ArturoMalucelli, GiulioAnanikov, Valentine P.Malvestiti, IvaniAnas, Andrea RoxanneMalwal, Satish R.Anastasescu, MihaiMaly, Kenneth E.Ancuceanu, RobertMaly, PavelAncuceanu, Robert ViorelMalyarenko (Vishchuk), Olesya S.Andanson, Jean MichelMalyarenko, Timofey V.Andera, LadislavMalyi, Oleksandr I.Andjelkovic, MirjanaMamane, VictorAndo, SugihiroMamiński, MariuszAndolfi, AnnaMammadova-Bach, ElminaAndonie, RazvanMan, AdrianAndrade Cetto, AdolfoManabe, KeiAndrade, Jeanette M.Manakhov, AntonAndrade, José CarlosManca, GabrieleAndrade, Paula B.Manca, Maria LetiziaAndrea, PenoniMancianti, FrancescaAndreev, Dmitrii E.Mancini, InesAndreev, KonstantinMancini, SimoneAndreoletti, PierreManconi, MariaAndreolli, MarcoMancuso, RaffaellaAndrés-León, EduardoMandal, AbhirupAndreu, InmaculadaMandal, Mihir KumarAndreuzzi, EvaMandalari, GiuseppinaAndrianasolo, EricMandity, IstvanAndrianov, AlexanderMandoli, AlessandroAndrić, FilipMandrich, LuigiAndricopulo, Adriano D.Mandrioli, RobertoAndruch, VasilMandrone, ManuelaANDRYSIK, ZdenekManera, ClementinaAnestopoulos, IoannisManet, IlseAngel, BárbaraManfredi, Kirk P.Angeletti, MauroManfredini, StefanoAngelici, GaetanoMangalagiu, IonelAngelico, RuggeroMangalara, Jayachandra HariAngelini, PaolaMangano, KatiaAngelo, RICCIOMangoni, AlfonsoAngelova, MariaManiam, SubashaniAngelova, MihaelaManjunath, ManuboluAngelova, Violina T.Mann, FrancisAngiolella, LetiziaMannell, HannaAniello, FrancescoManner, SuviAnifandis, GeorgeManner, Virginia W.Anjos, OféliaMannu, AlbertoAnraku, MakotoManousi, NataliaAnsari, RaisMansell, StephenAntal, DianaMansikkamäki, AkseliAntal, IstvánManta, StellaAntipova, VeronicaMantell, CasimiroAntolak, HubertMantell, LinAntonino, FamulariMantzourani, IoannaAntonio Speretto, RaulMantzouridou, FaniAntonio, MiceliManzo, CiroAntonopoulou, IoMao, ZongwanAnuta, ValentinaMaran, UkoAnzai, Jun-ichiMarc, DanielAnzenbacher, PavelMarchand, Patrice A.Aoki, DanMarchesan, SilviaApone, FabioMarcinčáková, DanaAppendino, GiovanniMarciniec, KrzysztofAppetecchi, Giovanni BattistaMarco, CareddaAprile, Francesco A.Marco-Contelles, JoséAquaro, StefanoMarco-Contelles, Jose LuisArai, Midori A.Marcotullio, Maria CarlaArambula, JonathanMaréchal, Jean-DidierAramesh, MortezaMarega, CarlaAranaz Corral, InmaculadaMargetić, DavorArancibia, RodrigoMargiolaki, IreneArand, MichaelMarhuenda, JavierAraniciu, CătălinMari, MicheleAraniti, FabrizioMaria, CorosAraújo, Maria Eduarda MachadoMarichev, KostiantynAraujo, William Reis DeMarín, Josefa IsasiArce, LourdesMarin, LuminitaArce, Pedro F.Marinescu, MariaArciello, AngelaMarini, ElisabettaArczewska, MartaMarini, FedericoArdejani, MaziarMarini, FrancescaArderne, CharmaineMarino, TizianaArduini, ArduinoMarins, MozartArean, Carlos OteroMarjanović, MarkoAreche, CarlosMarkiewicz, Wojciech T.Arena, KatiaMarkou, GiorgosAres, Alicia EstherMarkov, GabrielArgitis, PanagiotisMarkowicz-Piasecka, MagdalenaArguelles, SandroMarminon, ChristelleArias-Montaño, José-AntonioMarques, André MesquitaAricò, FabioMarques, Isabel PaulaAriga, HiroyoshiMarques, M. MatildeARIMITSU, SatoruMarques, Maria Manuel B.Ariño, AgustínMarquez, EdgarAriza, Maria TeresaMarra, AlbertoArmalyte, JulijaMarracci, SilviaArmeli Minicante, SimonaMarras, AlexanderArmenta, SergioMarraudino, MarilenaArmitage, BruceMarrelli, MariangelaArnau, AndrésMarrocchi, AssuntaArndt, Patrick G.Marsat, GaryArnesen, ThomasMarschner, ChristophAronica, Laura AntonellaMarsh, Neil G.Arosio, PaoloMarshall, PaulArrigo, RossellaMarta, Ferreiro-GonzálezArruda, Thomas M.Martelo, Liliana M.Arsene, Andreea LetiţiaMartens, JürgenArshad, AdeelMarti, Marcelo A.Artati, AnnaMartí, RaúlArteaga, OriolMartí, SergioArtetxe, BeñatMartín Palma, M. ElenaArtiaga, LuisMartin, Christopher B.Artola, MartaMartin, Francis LAsahara, HaruyasuMartin, Jose MiguelAsai, DaisukeMartin, KeithAsamizu, ShumpeiMartin, NathanielAscheri, JoseMartin, RachelAscrizzi, RobertaMartín-Aragón, SagrarioAsencios, Yvan J.O.Martín-Cabrejas, Maria AngelesAshley, JonMartínez de la Fuente, JesúsAshour, Mohamed LotfyMartinez De Lizarrondo, SaraAshpole, Nicole M.Martinez Martinez, VirginiaAshton, LornaMartinez Rodriguez, FlemingAskes, SvenMartínez, AAsnaghi, LauraMartínez, AlbertoAspatwar, AshokMartínez, AnaAšperger, DanijelaMartínez, AntonioAsquith, Christopher R. M.Martínez, CarmenAssaf, Khaleel IbrahimMartínez, SidoniaAssirelli, AlbertoMartínez-Ávila, GuillermoAstakhova, KiraMartinez-Calvo, MiguelAta, AtharMartínez-Carmona, MarinaAtabaev, TimurMartínez-Espinosa, Rosa MaríaAthanassiou, ChristosMartínez-García, MarcosAtobe, MasakazuMartinez-Gil, LuisAtuchin, Victor V.Martínez-Pérez, Carlos A.Au, Sam H.Martinez-Quiroz, MariselaAubert-Kato, NathanaelMartínez-Sánchez, NoeliaAuclair, NicolasMartínez-Tomé, María JoséAudette, Gerald FMartinez-Triguero, JoaquinAugustyniak, AdamMartinka, JozefAugustyniak, AdrianMartín-Martínez, Francisco J.Auh, Joong-HyuckMartino, GiuseppeAussenac, ThierryMartinotti, SimonaAuvynet, ConstanceMartín-Ramos, PabloAvato, PinarosaMartín-Rapún, RafaelAvdeeva, VarvaraMartins Amaro, Ana Paula BetencourtAverbeck, DietrichMartins, IanAverin, Alexei D.Martins, José A.Avila-Román, JavierMartins, M RosarioAvin, VijayMartins, Maria CristinaAviñó, AnnaMartins, NataliaAvramov Ivić, MilkaMartins, NatáliaAwasthi, VibhuduttaMartín-Vertedor, DanielAyala-Zavala, J. F.Maruyama, KeiAyat, NadiaMarverti, GaetanoAyub, Ricardo AntonioMaryasin, BorisAzam, FaizulMarzo, TizianoAzofra, Luis MiguelMarzocco, StefaniaAzuma, TakashiMarzorati, StefaniaAzuma, YasutakaMasato, DavideAzushima, KengoMasek, AnnaBaasov, TimorMashima, RyuichiBabaev, EugeneMasi, AnnalisaBabeva, TsvetankaMasi, MarcoBabichev, SergiiMasini, Jorge CesarBabickova, JankaMaslak, Veselin R.Babo, PedroMasłyk, MaciejBaby, AndreMassa, AntonioBach, BenoitMassadi, Omar AlBach, Duc-HiepMassari, SerenaBąchor, RemigiuszMassiani, PascaleBack, KyoungwhanMassiot, GeorgesBácskay, IldikóMassotte, DominiqueBacso, ZsoltMassoud, SalahBączek, KatarzynaMastinu, AndreaBadanjak Sabolović, MarijaMasullo, MariorosarioBadea Doni, MihaelaMasurier, NicolasBadea, Mihaela (Transilvania University of Brasov)Masuyer, GeoffreyBadea, Mihaela (University of Bucharest)Mateeva, NellyBadimon, Juan J.Mateo, CesarBadino, PaolaMateo, JavierBae, ChangdeuckMateo, ReyesBae, Ji-YeongMaterazzi, StefanoBae, Ki HyunMaterska, MałgorzataBaeza, AlejandroMatesanz, Ana I.Bagdziunas, GintautasMatesic, LidiaBagi, PéterMathivathanan, LogeshBagnoli, PaolaMatiadis, DimitrisBahn, AndrewMATI-BAOUCHE, NarimaneBahrim, GabrielaMatijašic, MarioBai, ChengyingMatikonda, SiddharthBai, RenrenMatilla-Hernández, AntonioBai, ShiMatin, AbdulBai, ZhengwuMatkowski, AdamBaier, AndreaMatoga, DariuszBailly, ChristianMatos, InêsBailly, FabriceMatos, ManuelBairaktari, EleniMatos, PauloBais, Manish V.Mátravölgyi, BélaBajda, MarekMatsoukas, ThemisBajda, TomaszMatsubara, MasahikoBajek, AnnaMatsuda, HisashiBajusz, DávidMatsuda, YuBak, AndrzejMatsuda, YudaiBakare, OladapoMatsufuji, HiroshiBaker, MatthewMatsugi, MasatoBaker, RobertMatsugo, SeiichiBaker, YsobelMATSUMOTO, ArimasaBalabanova, LarissaMatsumoto, HiroyukiBalakina, MarinaMatsumoto, ShojiBalandin, Alexander A.Matsumoto, TakayukiBalasingam, Suresh KannanMatsumoto, YasuhikoBalasubramanian, BalamuralikrishnanMatsunaga, KazuhisaBalawejder, MaciejMatsuo, YukikoBalcar, VladimirMatsuura, HiroshiBalcells, MercèMatsuura, KazunoriBalcerek, MariaMatsuzaki, KeiichiBaldassari, SaraMattei, CesarBaldassarre, Maria ElisabettaMattei, MichaelBaldini, MarioMatteis, Valeria DeBaldino, LuciaMatthias F., MelzigBaldisserotto, AnnaMattii, LetiziaBaldovini, NicolasMattivi, FulvioBálint, ErikaMatuszewska, AnnaBallante, FlavioMatwijczuk, ArkadiuszBallard, NicholasMatysiak, JoannaBalmaseda, JorgeMatysiak, WiktorBalme, SébastienMavrogonatou, EleniBalogh, György TiborMáximo, PatríciaBaloni, PriyankaMayan, Maria D.Baltakys, KęstutisMayol, LauraBamba, Bio Sigui BrunoMayumi, KoichiBan, YusukeMayzlish-Gati, EinavBanach, MarcinMazivila, SarmentoBanach, MateuszMazur, MarcelinaBanchelli, MartinaMazur-Bialy, Agnieszka IrenaBanchero, MauroMazurek, SylwesterBandiello, EnricoMazzeo, GiuseppeBandura, LidiaMazzeo, MinaBandyopadhyay, DebasishMazzone, GloriaBanerjee, AbhinandanMazzoni, LucaBanerjee, AditiMazzoni, RitaBanerjee, Hirendra NathMba Blázquez, MiriamBanerjee, KalpitaMbita, ZukileBanerjee, Shib ShankarMcAdam, JohnBanfi, FrancescoMcCall, KimberlyBani, DanieleMccarthy, PeterBankova, VassyaMcCubrey, JamesBanoub, Joe HMcdonald, PeterBanoub, JosephMcDougal, Owen M.Bansal, Kuldeep K.McFeeters, RobertBao, JinkuMcKay, DavidBao, YongpingMcLachlan, GerryBarakat, AssemMcLaughlin, EmilyBaral, Nawa RajMcLeish, Michael J.Baranowska-Bosiacka, IrenaMcMillin, MatthewBarata, Joana F. B.McMorran, DavidBarba, Francisco J.Mcreynolds, Katherine D.Barbalexis, PanagiotisMeana Paneda, RubenBarbaric, MonikaMears, JasonBarbault, FlorentMechrez, GuyBarbieri, GiovannaMedana, ClaudioBarbosa, OracioMedeiros, Adriane Bianchi PedroniBarbosa, Raquel De MeloMedellín-Castillo, NahumBarczak, MariuszMedici, SerenellaBarczyński, PiotrMedina, Gisselle N.Barding, Gregory A.Medina-González, YaocihuatlBarek, JiriMedina-Ramírez, IlianaBarišić, LidijaMediouni, SoniaBarker, DavidMedunic, GordanaBarker, Philip JMedvedovici, AndreiBarkoula, Nektaria MarianthiMeegan, Mary J.Barlocco, DanielaMehbub, Mohammad FerdousBaron, MarcoMehrazma, BanafshehBaroutaji, AhmadMehta, GauravBarraja, PaolaMei, Wen-LiBarrajon, EnriqueMei, YangBarral Silva, María TeresaMei, YeBarreca, DavideMeier, Robert J.Barreca, SalvatoreMeier-Menches, Samuel M.Barreira, JoãoMeijers, W.C.F.W.Barreto, MariaMeissner, AnjaBarreto-Bergter, ElianaMejuto, JuanBarros Gonçalves, Luciana RochaMekinić, Ivana GeneralićBarros, Marcelo PaesMele, GiuseppeBarroso Flores, JoaquínMeleady, PaulaBarrott, JaredMelguizo-Guijarro, ManuelBarszczewska-Rybarek, IzabelaMelini, ValentinaBartella, LuciaMella, JaimeBartelt, AlexanderMellor, Andrew L.Barth, AndersMelo, AndréBartocci, PietroMelo, JoãoBartolotti, MassimoMelo, Maria J.Bartosz, GrzegorzMelo, PedroBartyzel, AgataMelo, PriscillaBarwicki, JanMelo, TâniaBaryshnikov, GlibMelone, Mariarosa Anna BeatriceBasak, SujitMelucci, DoraBasile, LiviaMena-Rejon, Gonzalo J.Basilio Heredia, JoseMendes, AdrianoBasini, GiuseppinaMendez, FranciscoBasit, AbdulMendez-Sanchez, NahumBasith, ShaherinMendiola, JoséBaskaran, RathinasamyMendoza-Nuñez, Victor ManuelBassin, PaulMeneghetti, FiorellaBastida, AgathaMeneguzzo, FrancescoBastos, JulioMenet, Marie ClaudeBaswan, SudhirMenezes, Helvécio CostaBatinic-Haberle, InesMenezes, Irwin Rose AlencarBatista De Carvalho, Luís A.E.Meng, GeBatista, IrineuMeng, GuowenBatty, Kevin T.Meng, QingshiBatule, Bhagwan SahebraoMeng, WeinaBaudoin-Dehoux, CécileMeng, XiangchaoBaudoux, JérômeMeng, XiaoliBauer, StefanMeng, XiaomingBaumann, AnjaMenna, MarialuisaBaumann, MarcusMenshutina, NataliaBavaro, Simona LuciaMera, PaulaBavaro, TeodoraMeredith, LauraBaykov, SergeyMerfort, IrmgardBayona, JosepMergny, Jean-LouisBayraktar, EminMerighi, AdalbertoBazire, AlexisMerino, GabrielBazrafshan, ZahraMerlino, AntonelloBazwinsky-Wutschke, IvonneMeškys, RolandasBazylińska, UrszulaMesquita, Raquel B.R.Bazzarelli, FabioMessyasz, BeataBazzicalupi, CarlaMetaferia, WondwosenBeaudoin, DanielMetelka, RadovanBębenek, EwaMetharom, PatBeberok, ArturMeuti, MeganBec, KrzysztofMeyer, AndreasBec, Krzysztof B.Meyer, Anne S.Becerra, ManuelMeyer, MichelBeck, William T.Mezencev, RomanBecker, Donald F.Mezzetti, BrunoBednar, PetrMezzomo Collares, FabrícioBednarczyk, MarekMiao, TianxinBeharry, AndrewMiastkowska, MalgorzataBeidaghy Dizaji, HosseinMicale, NicolaBein, AmirMichaelis, David J.Bejerano, Eva EpeldeMichailidou, FrederikiBekhit, Alaa El-Din AMichal, KohoutBekiaris, GeorgiosMichalik, JanBelardinelli, Juan ManuelMichalska, AnnaBeld, JorisMichaud, PhilippeBelenky, PeterMichel, PiotrBelitsky, Jason M.Michelacci, Yara M.Belkova, Natalia V.Micheletti, GabrieleBell, Thomas W.Michels, AlexanderBellelli, RobertoMichl, ThomasBellemin-Laponnaz, StéphaneMichon, ChristopheBellich, BarbaraMicillo, RaffaellaBello, ClaudiaMieczkowski, AdamBello, Federica DalMiedzińska, DanutaBello, MartinianoMiękus, NataliaBellstedt, PeterMiele, Adriana E.Belt, TiinaMiele, Adriana EricaBenaglia, MaurizioMierczynska-Vasilev, AgnieszkaBencini, AndreaMiernyk, Ján A.Bencivenni, GiorgioMiguel, Maria da Graça CostaBencsik, TimeaMiguel, Sónia P.Bende, AttilaMiki, YasuhiroBendova, MagdalenaMikołajczak, BeataBenedetti, MicheleMikołajczyk-Bator, KatarzynaBenesperi, IacopoMikros, EmmanuelBenhar, ItaiMikstacka, RenataBéni, SzabolcsMikulski, DawidBenítez Jiménez, José JesúsMikuš, PeterBenítez-King, GloriaMilán-Carrillo, JorgeBenito, José ManuelMilardi, DaniloBenoist, HervéMilea, DemetrioBenoit, DenizotMilella, LuigiBenso, BrunaMiles, Wayne OwenBenter, ThorstenMiletić, Goran I.Bento, FatimaMiletín, MiroslavBenvenuti, StefaniaMilia, EgleBeranek, MartinMilic, NatasaBerardi, Anna ConcettaMilledge, John J.Berberan-Santos, MarioMiller, GavinBerdonosov, PeterMiller, GlennBerenjian, AydinMiller, LarryBerestetskiy, AlexanderMiller, Marvin J.Berger, MarkusMillet, MauriceBerger, Ralf G.Millet, ÓscarBergés Tiznado, Magdalena ElizabethMiloso, MariarosariaBergman, JanMilowska, KarolinaBergonzi, Maria CamillaMilton, IñakiBergougnoux, VéroniqueMimaki, YoshihiroBerkhout, BenMin, DuBerlanga, Ángel MéridaMin, HyeyoungBerlin, K. DarrellMin, Won LeeBerlowska, JoannaMinak, GiangiacomoBermeshev, MaximMinami, IchiroBermudez, MarcelMinami, MasaakiBermudez, MariaMinami, YasunoriBernal, Freddy AlexanderMinárovits, JánosBernard, Marek K.Minato, Ken-ichiroBernardi, LucaMinbiole, Kevin P. C.Bernardini, GiuliaMindt, ThomasBernat, PrzemysławMinetti, GiampaoloBernocchi, GraziellaMinghetti, PaolaBernuy-Lopez, CarlosMinjares-Fuentes, Jose RafaelBerrada, HoudaMinkiewicz, PiotrBerríos, CristhianMinofar, BabakBerski, WiktorMintcheva, NeliBertea, Cinzia M.Minutolo, FilippoBerteina-Raboin, SabineMira, Nuno P.Bertelloni, FabrizioMirabelli, CarmenBertin, Matthew J.Miranda, Jose M.Bertinaria, MassimoMiranda, KatrinaBerton, PaulaMirkhani, Seyyed AlirezaBertran, JoanMiroslaw, BarbaraBertrand, JustineMirza, SabiruddinBertrand, MartineMirzoev, TimurBesalú, EmiliMishra, AmritaBescond, JocelynMishra, Geetesh K.BESOMBES, ColetteMishra, Jay S.Bessa, LucindaMishra, Pawan KumarBessho, TadayoshiMishra, SasmitaBesson, ThierryMiskulin, MajaBesson-Bard, AngéliqueMita, GiovanniBettazzi, FrancescaMitakou, SofiaBeuerle, TillMitchell, CarterBevilacqua, AntonioMitchell, Scott G.Beyeh, Ngong KodiahMitchenko, SergeBezdetnaya-Bolotine, LinaMitomo, HideyukiBezerra, Daniel P.Mitran, GheorghiţaBezza, Fisseha AndualemMitropoulos, Athanasios C.Bhagia, SamarthyaMitsudo, KoichiBhandari, Dhaka RamMittal, NiteshBhandari, KhagendraMittelbach, MartinBhaskara, Govinal BadigerMitulovic, GoranBhattacharjee, Apurba KrishnaMiura, YoshikoBhattacharjee, SonaliMiyabe, HidetoBhattacharya, SupriyoMiyanaga, AkimasaBhavsar, Amit P.Miyata, JunBhosale, Sheshanath V.Miyata, YasuyoshiBhuiyan, AzadMiyatake, TomohiroBhusal, RamMiyoshi, DaisukeBiagi, MarcoMiyoshi, NorioBiagini, GiuseppeMiyoshi, NoriyukiBiałas, Adam J.Miyoshi, ToshikazuBiałek, MarzenaMizerska-Kowalska, MagdalenaBialk-Bielinska, AnnaMizobata, TomohiroBiałoń, MariettaMizuno, TakayukiBiałowiec, AndrzejMladěnka, PřemyslBian, HuiyangMladenović, MilanBiancardi, VivianMlejnek, PetrBianchi, AntonioMlostoń, GrzegorzBianchi, GiuliaMnif, WissemBianco, ArmandodorianoMobbili, GiovannaBiancolillo, AlessandraMocan, AndreiBiedermann, DavidMoczkowska, MałgorzataBielenica, AnnaModenutti, CarlosBiernasiuk, AnnaModica, MariaBiesaga, MagdalegaModukuri, RamkumarBigiani, AlbertinoMogilski, SzczepanBijak, MichalMohamed, Ahmed El RubyBijak, MichałMohamed, SharmarkeBikiaris, DimitriosMohamedali, KhalidBikov, AndrásMohammad Zafar, ImamBilal, MuhammadMohammed, AltafBilewicz, AleksanderMohammed, YousufBilin´ska, LucynaMohan, Velram BalajiBilitewski, UrsulaMohanram, HariniBilyachenko, Alexey N.Mohsen, AttayebBimonte, SabrinaMoineau-Chane-Ching, Kathleen I.Binello, AriannaMok, Daniel Kam-WahBini, MarcellaMokaya, RobertBirkemeyer, ClaudiaMoldovan, BiancaBirringer, MarcMoldoveanu, CostelBirsa, Lucian MMolina, MariaBisaglia, MarcoMolinie, RolandBisbey, RyanMolins, BlancaBisi, AlessandraMöller, HeikoBisio, AntonellaMollica Nardo, VivianaBišová, KateřinaMollica, AdrianoBisson, JonathanMollica, FrancescoBisson, LindaMolnar, MajaBisti, SilviaMolognoni, LucianoBistoni, GiovanniMoloney, Mark G.Bisz, ElwiraMoltzau, Lise RománBiziuk, MarekMonchaud, DavidBiziuk, Marek K.Monclus, MichelBizzarri, ClaudiaMondal, MukuleshBizzo, HumbertoMonfort, OlivierBkaily, GhassanMong, Mei-ChinBlaak, JürgenMonge, DavidBlack, DavidMonguzzi, AngeloBlacker, ThomasMoni, LisaBlackwell, BarbaraMoniuszko-Szajwaj, BarbaraBlais, Jean-FrançoisMonsees, ThomasBlanco, ÁngelesMontagnani Marelli, MarinaBlanco, AntonioMontalvo González, EfigeniaBlanco, MariaMontalvo-Rodríguez, RafaelBlandin, GaetanMontana, Angel M.Blasco, TeresaMontaño Priede, José LuisBlasi, FrancescaMontaño, Gabriel A.Blazevic, IvicaMonte Lara, Mª ConcepciónBłażewska, Katarzyna M.Montecchio, DanieleBloino, JulienMonteiro, AntóniaBloksgaard, MariaMonteiro, BernardoBlom, BurgertMonteiro, MarioBloor, StephenMonteiro-Neto, ValérioBlumer-Schuette, Sara E.Montenegro, IvánBober - Majnusz, KatarzynaMontenegro, LuciaBobis, OtiliaMontero, OlimpioBobrowski, KrzysztofMontes Morán, Miguel A.Bocchinfuso, GianfrancoMontesano, CamillaBoccia, Antonella CaterinaMontesano, DomenicoBocian, AleksandraMontesarchio, DanielaBoddy, Christopher NMontgomery, Jason M.Bode, Robert F.Monti, DanielaBodenstine, ThomasMontiel-Smith, SaraBoder, EricMontone, Carmela MariaBodo, EnricoMontouillout, ValérieBoeglin, AlexMontowska, MagdalenaBoga, CarlaMoo, JamesBogacki, JanMoody, David E.Bøgwald, JarlMoon, Byeong CheolBohn, TorstenMoon, Il SooBoichuk, SergeiMoon, Joon-KwanBojarová, PavlaMoon, JuhyukBojarski, AndrzejMoon, YuseokBojić, MirzaMora, LeticiaBokach, NadezhdaMorabito, RosannaBolanos-Garcia, Victor MMoraes, Valéria R. S.Bolboacă, Sorana D.Morak-Młodawska, BeataBölcskei, HedvigMorales Narváez, EdenBölcskei, KataMorales Suárez-Varela, María M.Bolotin, Dmitrii S.Morales, JavierBolotov, LeonidMorales, Maria LourdesBombard, SophieMorales, PaulaBommagani, ShobanbabuMorales-Cruz, AbrahamBonadies, IreneMorales-de La Peña, MarianaBonamore, AlessandraMorales-González, José AntonioBonanomi, GiulianoMorales-Morales, DavidBondon, ArnaudMoran, JoseBondzior, BartoszMoran, WesleyBonikowski, RadosławMoran, YehuBonkovsky, Herbert L.Moreau, RegisBonnet, FannyMoreira Gonçalves, LuísBonnet, PascalMoreira Martínez, Ramón FelipeBonnet, Pierre-AntoineMoreira, Ana Sofia PereiraBonni, ShirinMoreira, José Claudio FonsecaBonomo, MatteoMoreira, Vânia M.Bonyár, AttilaMoreno, Diego A.Boo, Yong ChoolMoreno, J.J.Boon, Elizabeth M.Moreno-Piraján, Juan CarlosBorbas, AnikoMoreno-Rojas, José ManuelBorbás, AnikóMoreto, FernandoBorbas, K. EszterMorgan, AlexanderBorbone, NicolaMorgan, Rhianna K.Borcan, FlorinMorgat, ClémentBordiga, MatteoMorgera, Alessandro FraleoniBorfecchia, ElisaMori, KeijiBorges, Alisson CarraroMori, KohsukeBorges, AnabelaMori, MattiaBorges, RicardoMori, SeijiBorges, RosivaldoMori, ShigekiBoris, PejinMorigi, RitaBoros, LaszloMorikawa, SatoruBoros, LászlóMorikawa, ToshioBorota, AnaMorimoto, MasanoriBoroujerdi, RaminMorimoto, YumaBorovac, Josip A.Morimura, ShigeruBorowicz, MarcinMorini, LucaBorrego, Ana AriasMorio, FlorentBorriello, LuciaMORISAWA, YusukeBortolozzi, RobertaMorita, NaokiBorup Jensen, SvendMorita, NobuyoshiBorys, KrzysztofMoritz, MichałBorzenkov, MykolaMoriyama, KatsuhikoBoscá, FranciscoMorris, DonBoschi-Muller, SandrineMorris, JamesBoschin, GiovannaMortara, LorenzoBose, DebojitMosca, LucianaBose, SaurabhMoškon, MihaBoshir Ahmed, MohammadMossialos, DimitrisBoskovic, ZarkoMossine, Valeri V.Bosse, Jens B.Mostafavi, MehranBosserhoff, AnjaMostovich, EvgenyBosso, AntonellaMot, AugustinBotana, Ana MaríaMota, Antonio J.Botelho, Bruno G.Mota, ManuelBotelho, Monica C.Mota-Morales, Josué D.Boti, VasilikiMotokura, KenBotrè, FrancescoMou, YongchaoBotta, BrunoMoujir, Laila MoujirBotticella, ErmelindaMoura, Nuno M. M.Boualy, BrahimMourdikoudis, StefanosBoucher, David S.Mourtas, SpyridonBoudaden, JamilaMourtzinos, IoannisBouquillon, SandrineMoustafa, Ahmed A.Bour, PetrMoyano, AlbertBourdon, EmmanuelMoyano-Mendez, Jose RamonBoury, BrunoMozo, J. D.Bowen, JamesMphahlele, Malose JackBoyd, Robert D.Mrlík, MiroslavBoylan, FabioMroczek, AgnieszkaBozano, LuisaMroczek, TomaszBozdag, MuratMshvildadze, VakhtangBozhko, Yulia Yu.Mu, Q.X.Bozonet, StephanieMu, RuipuBožović, MijatMucci, AdeleBracher, FranzMuccilli, VeraBrachet, EtienneMuchtaridi, MuchtaridiBradley, LizMucignat, CarlaBradshaw, BenMück-Lichtenfeld, ChristianBraga, Susana SantosMucsi, ZoltánBraham Deep Singh, DhillonMuehlmann, Luis AlexandreBrancart, JoostMugica, VioletaBranchini, AlessioMühle, ChristianeBranco, VascoMukaiyama, AtsushiBrandt, CurtisMukherjee, RajibBrandt, RolandMukherjee, SudipBrant, Jonathan A.Mulas, MaurizioBrás, NatérciaMulcahy, BenBraun, Frank K.Muller, ChristianBravo, Francisca IsabelMüller, ChristophBravo, JeronimoMüller, Jochen A.Brčić Karačonji, IrenaMüller, ThomasBrebu, MihaiMulloy, BarbaraBrecker, LotharMun, JiyoungBregadze, VladimirMünch, AlexanderBrehm-Stecher, ByronMunday, MichaelBreidbach, AndreasMuniz-Miranda, FrancescoBreier, AlbertMuniz-Miranda, MaurizioBren, UrbanMuñoz, Marcelo AndrésBrenna, MariaMuñoz-Bonilla, AlexandraBrenna, StefanoMuñoz-Rugeles, LeonardoBrestic, MarianMunteanu, Florentina-DanielaBreton, Gary W.Murakami, KazumaBreza, MartinMurakami, KosakuBrichacek, MatthewMurakami, YasufumiBrigido, ClarisseMuramoto, KojiBrito-Parada, Pablo R.Muranaka, AtsuyaBrocławik, EwaMurata, ToshihiroBroderick, Tom L.Muratov, Eugene NBrogi, SimoneMuravyev, Nikita VBronisz, RobertMuravyev, Nikita V.Brooks, Tracy A.Murayama, HidekiBrouwers, Jos F.Murcia, J.J.Brovarets, Volodymyr S.Muresan, VladBrown, AngelaMurillo, WalterBrown, LindsayMurkin, AndrewBrudzynski, KatrinaMurkovic, MichaelBrullo, ChiaraMurphy, Caroline SBrumm, Phillip J.Murphy, Coleen T.Brunet, ChristopheMurphy, CormacBrunetti, CeciliaMurray Stewart, TracyBruni, GiovannaMurray, Jane S.Brunschweiger, AndreasMurray, JeremyBruserud, ØysteinMurry, Daryl J.Bryant, Matthew S.Muschiol, JanBrycki, BogumilMusgrave, IanBryk, PawełMusilek, KamilBryszewska, Malgorzata AnitaMusioł, RobertBrzezinski, MarekMusmarra, DinoBrzoska, Malgorzata MichalinaMusso, LoanaBubici, GiovanniMus-Veteau, IsabelleBuchberger, WolfgangMuszyńska, BożenaBuchet, ReneMuszyńska, EwaBucio, EmilioMuthana, MunittaBuckeridge, JohnMuthusamy, GovarthananBuckley, LeoMuthusamy, NagendranBuckman, JimMuto, AkiraBuckner, Steven W.Muziol, Tadeusz MikolajBudnikova, YuliaMyllymaa, SamiBudryn, GrażynaMysiorek, CarolineBudyka, Mikhail F.N’DO, Jotham Yhi-pênêBudzak, SimonNa, DokyunBudzisz, ElżbietaNa, Dong HeeBueno-Silva, BrunoNa, KyungsuBufo, Sabino AurelioNabok, AlexeiBugarin, AlejandroNaccarato, AttilioBuica, AstridNachon, FlorianBuisson, DidierNader, HelenaBujanovic, BiljanaNaderi, SoheilBukin, OlegNadolny, ZbigniewBulgariu, LauraNaftalin, RichardBuller, JensNagaki, AiichiroBullitt, EstherNagarajan, BalajiBumgardner, Joel D.Nagasawa, KazuoBun NG, TziNagata, ToshiBunce, Richard A.Nagatani, NaokiBunik, VictoriaNagatsugi, FumiBuranaj Hoxha, BesmirNagel, RaimundBuratta, SandraNagy, MiklósBURCUL, FrankoNahar, LamiaBureau, RonanNair, Devatha P.Burke, AnthonyNair, RemyaBurke, BenjaminNair, Sandeep SudhakaranBurlacu, AlexandrinaNajda, AgnieszkaBurns, D. ThorburnNajlah, MohammadBurns, Jonathan R.Naka, AkinobuBurns, Jorge S.Nakagawa, ToshinoriBurpo, Fred J.Nakagawa, YukiBurridge, Paul W.Nakagawa-Goto, KyokoBurrola-Aguilar, CristinaNakahama, Ken-ichiBurzuri, EnriqueNakai, HiroyukiBus, James S.Nakamichi, NoritakaBusardò, Francesco PaoloNakane, TakanoriBusquets, Maria AntòniaNakano, HideyukiBusto, NataliaNakashima, KenichiBustos, Diego MartinNakashima, TakuyaBustos-Terrones, YanethNakata, NorioBuszewicz, GrzegorzNakatani, NaokiButler, Mark S.Nakatani, YoshihikoBuzuk, MarijoNakayama, YasumuneBydlowski, Sérgio PauloNakayama, YuushouByler, KendallNaliwajko, SylwiaByrdin, MartinNallasamy, PalanisamyByun, JeehyeNallu, SumithaCaballero, AntonioNam, KwanghoCaballero, JulioNam, Tae GyuCabanillas, BeatrizNamasivayam, VigneshwaranCabot, CatalinaNambeesan, SavithriCabral, CéliaNamiesnik, JacekCabrele, ChiaraNanaki, StavroulaCabrera, HumbertoNanjundan, MeeraCābulis, UģisNano, AdelaCacciola, FrancescoNapoli, Edoardo MarcoCacho Teixeira, MiguelNapolitano, AlessandraCádiz-Gurrea, María De La LuzNardi, MonicaCadwallader, KeithNardini, MirellaCaeiro, MariaNardoni, SimonaCaffrey, PatrickNaryzhny, StanislavCahill, PaulNasarre, PatrickCahlíková, LucieNassar, Zeyad D.Cai, ChaoNastasa, CristinaCai, LishengNatalello, AntoninoCai, WenlongNatalicchio, AnnalisaCain, RickyNatarajan, ArutselvanCaires, Hugo R.Natarajan, Sathish KumarCairrão, ElisaNath, ArijitCais-Sokolińska, DorotaNaumann, StefanCalabrese, LuigiNaumovski, NenadCalabrò, FrancescoNaushad, MCalabrò, Paolo S.Navalon, SergioCalani, LucaNavarra, MicheleCalarco, AnnaNavarrete-Vazquez, GabrielCalcutt, MichaelNavarro, DanielaCaldeira, IldaNavarro, RodrigoCaldeira, MichaelNavarro-Hoyos, MirthaCaldera, FabrizioNaviaux, Robert K.Caldo, Kristian MarkNavickiene, SandroCaligiuri, IsabellaNaviglio, DanieleCalinescu, IoanNawirska-Olszańska, AgnieszkaCalleja, MontserratNawrot, BarbaraCallery, Patrick S.Naylor, AmyCallone, EmanuelaNazare, MarcCalogeropoulou, TheodoraNazarski, Ryszard B.Calokerinos, Antony C.Nazemi, AliCalpena, Ana C.Nazemifard, NedaCalvano, Cosima DamianaNazzaro, FilomenaCalvete, MarioNdieyira, JosephCalvo, ConceptiónNeațu, FlorentinaCamacho-Corona, M. R.Neda, IonCâmara, José SousaNedialkov, Paraskev T.Camblor, Miguel A.Nedialkov, Paraskev TodorovCamele, IppolitoNeels, OliverCameli, NormaNees, MatthiasCamerlingo, CarloNeffe, Axel T.Cameron, JosephNegri, GiuseppinaCaminade, Anne-MarieNegrón-Silva, Guillermo E.Camins, AntoniNehira, TatsuoCamire, Mary EllenNehmé, ReineCamp, CédricNelyubina, YuliaCampagne, Jean-MarcNemec, IvanCampbell, LeeNemnes, George AlexandruCampbell, MichaelNemoto, NobukatsuCampestre, CristinaNemzer, BorisCampiglia, PietroNenajdenko, Valentine G.Campillo, NataliaNeng, NunoCampo, RiccardoNeophytou, Christiana M.Campos Rosa, Joaquín MaríaNesmelov, Yuri E.Campos, FranciscoNesterov, DmytroCampos, MariaNesterova, Irina V.Campos-Martínez, J.Nestler, UlfCamproux, Anne-ClaudeNetscher, ThomasCamps, IhosvanyNeugebauer, DorotaCanals, Joan MiguelNeunert, GrazynaCancemi, PatriziaNevarez-Moorillon, Guadalupe VirginiaCanela-Garayoa, RamonNeves, VeraCangialosi, DanieleNewton, JasonCanini, AntonellaNg, CarolineCannavo, AlessandroNg, Ho LeungCannazza, GiuseppeNg, Kenneth Kai-SingCannon, Ronald E.Ng, Qin XiangCanselier, Jean PaulNg, Wei LongCantoral, Jesús ManuelNgai, Ming-YuCao, JieNgene, PeterCao, ShaotaoNgo, Hien T.T.Cao, ShugengNgo, Hoan ThanhCao, XuNgomba, RichardCao, YuheNguewa, PaulCaparros-Martin, JoséNguyen, Ngoc A.Caparrotta, StefaniaNguyen, Thanh BinhCapasso, RaffaeleNguyen, TrieuCapece, AngelaNicasio, Maria CarmenCapogni, MarcoNiclos Gutiérrez, JuanCappariello, AlfredoNicolas, CyrilCappello, TizianaNicolás, Francisco E.Capriati, VitoNicoletti, FerdinandoCapsoni, DorettaNicoletti, RosarioCarabineiro, SóniaNicoletti, VincenzoCarafa, MariaNicolotti, OrazioCaravan, PeterNiculau, EdenilsonCarbonaro, Carlo MariaNie, ChuanxiongCarbonell-Barachina, AngelNiedzielski, PrzemysławCardenas Zuniga, RobertoNiedzwiecki, LukaszCárdenas, SoledadNiehaus, Thomas D.Cardenia, VladimiroNielsen, Peter E.Cardey, BrunoNielsen, Vance G.Cardin, ChristineNieminen, KaarloCardinale, JensNiemirowicz-Laskowska, KatarzynaCardona, FrancescaNieto, PedroCardoso, DavidNikas, Spyros P.Cardoso, SusanaNiki, EtsuoCardoso, Susana M.Nikitenko, Sergey I.Cardoso, V.LNikolaev, ViacheslavCardullo, NunzioNikolaevskii, Stanislav A.Care, AndrewNikolaidis, Alexandros K.Carelli, StephanaNikolaidis, NikolaosCargnoni, FaustoNikolakakis, IoannisCaridi, AndreaNikolakopoulou, Angeliki M.Cariglia, MarcoNikolova, RositcaCariou, KevinNiles, Richard M.Carlberg, CarstenNimse, Satish BalasahebCarlini, Célia R.Ning, XuhuiCarmela, ConidiNishi, KosukeCarmela, SaturninoNishi, NaoyaCarmona-Ribeiro, Ana M.Nishimoto, YoshioCarnevale Neto, FaustoNishimura, ChiakiCaro, YanisNishinaga, TohruCarocho, Márcio SoaresNishiwaki, HisashiCarotenuto, MarcoNishiwaki, NagatoshiCarotti, AngeloNishiyama, AkihitoCarpentier, RodolpheNishiyama, YusukeCarqueijeiro, InêsNiskanen, Einari A.Carradori, SimoneNisnevitch, MarinaCarrara, MariaNissinen, MaijaCarrasco, HéctorNistor, CristinaCarrasco, SergioNitsche, ChristophCarré, VincentNitulescu, George MihaiCarreno, NeftaliNitulescu, MihaiCarrer, AlessandroNiu, GuangleCarrera, Ceferino A.Nixon, GemmaCarrera, LucreciaNoël, SébastienCarrieri, AntonioNoghero, AlessioCarrion, M. DoraNoguera, Nélida InésCartron, Pierre-FrançoisNogues, IsabelCarullo, GabrieleNoh, KeumhanCaruso, GiuseppeNokami, ToshikiCaruso, Joseph A.Nomngongo, Philiswa NosizoCarvajal-Millan, ElizabethNoor, NuruzzamanCarvalho Pimenta, DanielNorbury, Luke JamesCarvalho, IvoneNoren Hooten, NicoleCarvalho, PauloNorman, KennethCasado-Gavalda, Maria P.Norton, MichaelCasapullo, AgostinoNoshita, ToshiroCasares, JuanNosoudi, NasimCasassa, Luis FedericoNovak, JuricaCascione, MariafrancescaNovak, LajosCaseri, WalterNovak, ZoranCassani, Maria CristinaNovello, VittorinoCastaldo, RacheleNovič, MarjanaCastanheira, Elisabete M.S.Novikov, Alexander S.Castanho, MiguelNovikov, AndreiCastell Escuer, MargaridaNovío, SilviaCastellini, ElenaNovoveská, LucieCastiglione, FrancaNowacka, MalgorzataCastillo, SandraNowacka-Jechalke, NataliaCastro, José Javier FernándezNowacki, AndrzejCastro, María ÁngelesNowak, AdrianaCastro, Ricardo Dias DeNowak, DariuszCastro-Muñoz, RobertoNowak, DorotaCastro-Vargas, Henry I.Nowak, RenataCastrucci, Ana MariaNowak, WieslawCasula, MicheleNowicki, JanuszCatalan, MarceloNowicki, RomanCatani, MartinaNowotarski, ShannonCatarino, IsabelNtaikou, IoannaCatarino, Marcelo D.Ntalli, Nikoletta G.Catenacci, LauraNthunya, Lebea N.Catizzone, EnricoNtie-Kang, FideleCatto, MarcoNtougias, SpyridonCatucci, LuciaNumez, EstrellaCauda, ValentinaNuñez, AlbertoCavalcanti Da Silva Filho, EdsonNunez, OscarCavalcanti, João Henrique F.Nuñez, RosarioCavallaro, GiuseppeNurchi, Valeria MCavallo, EugenioNurtdinov, RamilCayuela-Sánchez, José AntonioNuţǎ, Diana CameliaCeccarelli, GabrieleNuthikattu, SaivageethiCeccarelli, MatteoNuzzo, DomenicoCecchetti, SerenaNuzzo, GenoveffaCejkova, JitkaNycz, JacekCelano, LauraNycz, Jacek E.Celeiro, MaríaNyman, Tuula AnneliCellamare, SaverioO’Doherty, GeorgeCen, ShanO’Keefe, Sean F.Ceña, ValentínO’Leary, SeánCendrowski, AndrzejO’Neil, GregoryCen-Pacheco, FranciscoO’Reilly, StevenCeretti, MonicaObara-Michlewska, MartaČerný, JiříObata, TohruCerny, MiroslavObot, I.B.Ceron-Carrasco, JoseObreza, AlešCerón-Carrasco, José PedroOcampo-García, Blanca ElíČeřovský, VáclavOchędzan-Siodłak, WiolettaCerqueira, FatimaOchi, ShinichiroCerra, BrunoOdelius, KarinCervino, GabrieleOdell, Luke R.Cesano, FedericoOfir, RivkaCesarino, IgorOftadeh, ShahinČesla, PetrOgino, IsaoČesonienė, LaimaOgnyanov, ManolCéspedes Acuña, Carlos LeonardoOgórek, RafałCha, ChaenyungOgra, YasumitsuCha, Sang-HoOgrinc, NivesChaboussant, GregoryOgungbe, IfedayoChachami, GeorgiaOh, Eun-TaexChacko, Jenu VargheseOh, HyunchulChae, HeeyoungOh, JaehyeonChai, Kyu YunOh, Jae-MinChai, YifengOh, Jae-WookChaimbault, PatrickOh, SangtaekChakrabarti, SubhadeepOh, Yoon SinChakraborty, RajaOhara, KeishiChakraborty, SayanOhashi, NamiChaleckis, RomanasOhisa, SatoruChalivendra, Subbaiah C.Ohnishi, MasatoshiChamaani, AmirOhno, OsamuChambaud, GilberteOhsedo, YutakaChambers, EdgarOHTA, KiminoriChamkha, AliOhta, ShinjiChan, Ben C. L.Ohto, KeisukeChan, Jerry C. C.Ohto, TatsuhikoChan, Kuan RongOjeda, JesusChan, Li-HsinOjeda-May, PedroChan, SunneyOjha, Shreesh K.Chan, WanOjovan, MichaelChan, Yi-TsuOjstršek, AlenkaChan, Yu-YiOkada, TaketoChance, R. J.Okada, YoshiharuChandler, KevinOkaiyeto, KunleChang, Che-ChienOkajima, TetsuyaChang, Cheng-ChungOkamoto, YoshiyukiChang, Chia MingOkazawa, AtsushiChang, Chia-cheOkuda-Ashitaka, EmikoChang, Chia-ChenOkumura, TadayoshiChang, Chih-WeiOláh, AttilaChang, Ching-yaoOlaru, Octavian TudorelChang, Dong WookOldham, LarryChang, Fang-RongOlea, Andres F.Chang, Geng-RueiOledzka, EwaChang, Hsueh-WeiOlejar, Kenneth J.Chang, Hsun-ShuoOlejniczak, Agnieszka B.Chang, Jui-ChengOlejnik, AnnaChang, Nan-ShanOlejnik, MalgorzataChang, Sue-JoanOlejníková, PetraChang, Te-ShengOlennikov, DaniilChang, Yuan ShiunOliva, Alexis M.Chang, Yu-WeiOliva, JoséChankhamjon, PranatchareeyaOlivares-Reyes, Jesús AlbertoChantana, JakapanOliveira, Alcineia C.Chao, Chih-HuaOliveira, AndreiaChao, Pi-YuOliveira, Eduardo De JesusChao, Wen-WanOliveira, Guedmiller S.Chapelle, DavidOliveira, Hugo M.Chapoval, Svetlana P.Oliveira, José M.Chaptal, VincentOliveira, Maria ConceiçãoCharbonneau, PatrickOliveira, Paula A.Charkhkar, HamidOliveira, RodrigoCharles, Marie ThérèseOlivero-Verbel, JesusCharvátová, HanaOliviero, GiorgiaChase, P. BryantOlova, NellyChassagnon, IreneOlsen, Christopher M.Chatterjee, RuchiraOlsen, ThomasChatterjee, SudiptaOlsovska, JanaChatzidimitriou, EffimiaOlszewska, Monika AChatzifragkou, AfrodotiOmar, FayeChatzopoulou, PaschalinaOmar, Syed HarisChau, Chi-FaiOms, OlivierChaudhary, VarunOms-Oliu, GemmaChaudhuri, Arunima Onchoke, KefaChauhan, HarshOndrias, KarolChauhan, NeerajOnduka, ToshimitsuChava, Rama KrishnaOnel, BuketChe, Chun-TaoOng, Eng ShiCheca, AntonioOniga, IlioaraCheel, JoséOniga, Smaranda DafinaChegaev, KostantinOniszczuk, AnnaChekmenev, Eduard Y.Onnis, ValentinaChellan, NireshniOno, Mario AugustoChemat, FaridOnysko, PetroChen, AizhengOon, Hazel H.Chen, BernardOostenbrink, ChrisChen, ChaoOpara, Elizabeth I.Chen, ChiOpatz, TillChen, ChiachungOpitz, JörgChen, Chia-YunOppert, BrendaChen, Chih-YenOprea, Corneliu IoanChen, Chuan-FengOpricǎ, LăcrămioaraChen, Chun-ChiOrczyk-Pawiłowicz, MagdalenaChen, Chun-JungOreshonkov, AleksandrChen, DaiqinOrian, LauraChen, GuangpingOrlando, GiustinoChen, GuanjunOrlando, TomasChen, GuoxunOrodnez, MarioChen, HaixiaOroian, Mircea AdrianChen, Han-QingOrozco, JahirChen, Hao PingOroz-Guinea, IsabelChen, HaoqingOrsolic, NadaChen, HaotongOrtega, María JesúsChen, HaoyuanOrth, James D.Chen, HongweiOrzelska-Górka, JolantaChen, HongzhangOsada-Oka, MayukoChen, Hsin-ChunOsajima, Josy A.Chen, Hsing-YinOsborn, HelenChen, Hsuan-YingOsma, JohannChen, Jen-TsungOśmiałowski, BorysChen, Jhy-DerOsório, Wislei R.Chen, JianboOspina-Millán, Claudia A.Chen, JiangangOstacolo, CarmineChen, Jian-LianØstergaard, JesperChen, Jiann-ChuOstovar, SaeidehChen, JinbiaoOstrowska, KingaChen, JinhuaOswald, IainChen, Jung-YaoOtero, AnaChen, Liang-YuOtero, CarolinaChen, Lih-GeengOtero, José A.Chen, LipingOtero-Espinar, Francisco JavierChen, Miaw-LingOtmačić Ćurković, HelenaChen, MingliOtręba, MichałChen, MingshengOtsuka, HideakiChen, MingzhouOtsuka, MasamiChen, Po-YuanOtsuka, YuzuruChen, Qiao-HongOtsuki, Takemichen, shuangOtt, MariaChen, ShuoOttria, RobertaChen, Shuo-BinOtulak-Kozieł, KatarzynaChen, SuzieÖtvös, Ferenc L.Chen, Teng-MingOu, Tian-miaoChen, TongOuazzani, JamalChen, Wei-TingOuellet-Plamondon, ClaudianeChen, WenchunOuyang, Xiao-kunChen, XiaOvando Martínez, MaribelChen, Xiao-LanOvidi, ElisaChen, XiaolinOwens, FredChen, XingOya, YuichiChen, XiuminOyama, DaiChen, XuechengOza, JavinChen, Yan TaoOzaki, ToshinoriChen, Yi-ChunOzawa, YoshikiChen, YingP. Morgado S. Neves, Maria Da GraçaChen, YuePabit, SuzetteChen, Yue-WenPacheco, NeithChen, Yu-KuoPacheco-Catalán, DaniellaChen, ZhaoPacheco-Fernández, IdairaChen, ZhePaci, MaurizioChen, ZhihangPacifico, SeverinaChen, ZhijunPacios, Luis F.Chen, ZhongningPaciulli, MariaChénais, BenoitPacor, SabrinaCheng, ChengPadilla-Martínez, Itzia I.Cheng, GangPaduano, AntonelloCheng, Hung-ChiPaduch, RomanCheng, Juei-TangPaek, Seung-mannCheng, Jya-WeiPaeng, KijungCheng, QingqingPaeschke, KatrinCheng, TaoPagani, StefaniaCheng, XiaomingPaganin, Valdecir AntonioCheng, Yuan-BinPagano, BrunoCheng, Yu-ShenPage, StephenCheng, ZhePagnacco, Maja C.Cherkasov, ArtemPagniez, FabriceChern, EdwardPagnotta, StefanoCherng, Jyh-ShiarnPaic, FraneChetcuti, Michael J.Paier, JoachimChetwynd, AndrewPaik, Hyun-DongChhabra, GaganPaik, Soon YoungChia, Win-LongPainter, GavinChianese, GiuseppinaPaiva, SandraChiang, Anthony S.T.Paixao Coelho, Jose AugustoChiang, Chin LungPakharukova, MariyaChiang, Hsiu-MeiPal, RiturajChiang, Meng-TsanPal, RumpaChiang, NaihaoPalacios, FranciscoChiang, Wen-DeePalacios, JavierChiang, Yi-TingPalacios-Santander, José MaríaChiappinelli, KatherinePalage, MarianaChiarelli, LaurentPalani, ChitraChiarini, MarcoPałasz, ArturChiarotto, IsabellaPale, PatrickChiasera, AlessandroPalenzuela López, José AntonioChichorro, Juliana G.Palermo, AmeliaChien, Shih-ChangPalikaras, KonstantinosChien, Yin-HsiuPalko-Labuz, AnnaChieppa, MarcelloPalla, FrancoChiessi, EsterPallandre, AntoineChifiriuc, CarmenPallavicini, PiersandroChilaka, Cynthia AdakuPalleschi, VinceChilders, Delma SusanPallottini, ValentinaChin, Young-wonPalma, MatteoChinchilla Salcedo, NuriaPalma, MiguelChinchilla, RafaelPalmieri, AlessandroChinthakindi, PraveenPalmisano, GiovanniChiou, Chun-TangPalomba, LetiziaChiou, Shih-HwaPalombi, LauraChira, KleopatraPalomo, ValleChirila, TraianPalù, GiorgioChis, VasilePalyi, GyulaChisaka, MitsuharuPampana, SilviaChisholm, John D.Pan, MiaoChitchumroonchokchai, ChureepornPan, XiaoyueChitescu, Carmen LidiaPan, YeChiu, Tai-chiaPanaro, Maria AntoniettaChiu, Tsai-HsinPande Katare, DeepshikhaChizzola, RemigiusPanderi, IreneChlebek, JakubPandey, Ramesh PrasadChlichlia, KaterinaPandey, RaviChmielarz, PawełPandey, SudipChmielewska, AleksandraPandit, SantoshCho, Dae WonPandith, AnupCho, Hea-YoungPanek, RafalCho, Jae YoulPaneth, AgataCho, Kyoung SangPaneth, PiotrCho, Seung-SikPang, BoCho, SungeunPanglisch, StefanChocholouš, PetrPanigrahi, MrutyunjayChoi, HyukjaePannerselvam, SureshChoi, Jae SuePannkuk, EvanChoi, Jae-HongPantazaki, Anastasia A.Choi, Jane RuPanzarasa, GuidoChoi, Keun-HyungPanzella, LuciaChoi, Ki ChoonPaolillo, MayraChoi, Min-KooPaolino, MarcoChoi, Soon MoPapadakis, RaffaelloChoi, Yeung JoonPapademas, PhotisChoi, Yong JunPapadimitriou, VassilikiChoi, Young HaePapadopoulos, Antonios N.Choi, Young HeePapaefstathiou, GiannisChoinopoulos, IoannisPapageorgiou, MariaChojnacka, AnnaPapagiannopoulou, DionysiaChojnacki, JarosławPapagni, AntonioCholewinski, GrzegorzPapaioannou, DionissiosChong, Parskson Lee GauPapakyriakou, AthanasiosChoo-Smith, Lin-P’ingPapale, MariaChorazy, SzymonPapanastasiou, IoannisChou, YachangPapetti, AdeleChow, Franklin Wang-NgaiPapi, AlessioChowański, SzymonPapoti, Vassiliki T.Chowdhury, RatulPappa, AglaiaChoy, Francis Y.M.Pappas, ChristosChrissafis, KonstantinosParadisi, FrancescaChristensen, Lars P.Paradiso, VeronicaChristoforides, EliasParajuli, DurgaChrobak, ElwiraParashar, DeepakChruściel, Jerzy J.Pardi, NorbertChruszcz, MaksymilianPardío-Sedas, Violeta T.Chu, JiaxiangPardo, LuiChuang, Show-MeiParedes, José ManuelChuang, Ta-HsienParida, SheetalChueh, Pin JuParikesit, Arli AdityaChun, Ock K.Park, Chan HoCHUNG, JING-GUNGPark, Chong-WookChung, Man-KyoPark, ChulhwanChung, SungjinPark, DaehwanChuu, Chih-PinPark, DaesungChuyen, Hoang V.Park, Deog-SuChwil, MirosławaPark, DianeChyau, Charng-CherngPark, Dong HyukCiaccheri, LeonardoPark, Dong SunCiancaleoni, GianlucaPark, DongheeCianciosi, DanilaPark, Eun-youngCiardelli, FrancescoPark, Han-A.Ciardiello, Maria AntoniettaPark, HanjungCicala, GianlucaPark, Il-KwonÇiçek, Serhat SezaiPark, JinhaCicenas, JonasPark, JunseongCicero, ArrigoPark, Kyoung SikCicero, NicolaPark, Min-JungCichero, ElenaPark, MiraCichorek, MiroslawaPark, Myung HwanCielecka-Piontek, JudytaPark, Sang WonCieplak, MaciejPark, Sung-GyooCieslar, GrzegorzPark, SungnamCieslik, WioletaPark, SungsuCifone, Maria GraziaPark, WansuCimmino, GiovanniPark, Woong JuneCimpoiu, ClaudiaParker, Jane K.Cinteza, Otilia LudmilaParmeggiani, FabioCiobica, AlinParnian, Mohammad JavadCioffi, FedericaParolini, CinziaCiolacu, DianaParpinello, GiuseppinaCione, ErikaParra, AlejandroČipak Gašparović, AnaParra, MargaritaCirri, DamianoParra-Saldívar, RobertoCirrincione, GirolamoParrella, EdoardoCiszewski, WojciechParrino, BarbaraCiszewski, Wojciech MichałParrino, FrancescoCitores, LuciaParsell, TrentonCiudad, CarlosParsonage, DerekCiulu, MarcoParthasarathy, AnutthamanČizmić, MirtaParus, AnnaClanchy, FelixPârvu, Alina ElenaClaramunt, Rosa M.Parzonko, AndrzejClarke, AnthonyPasán, JorgeClarke, PaulPascali, GiancarloClaudia, Delgadillo PugaPaschke-Kratzin, AngelikaClaus, HaraldPaschoal, C.W.A.Clément, GillesPaschos, Georgios K.Clément, NathaliePáscoa, RicardoClementi, CatiaPasha, F. A.Cliff, Margaret A.Pashikanti, SrinathCliment, VíctorPasini, DarioClodoveo, Maria LisaPasinszki, TiborCobo, MarthaPasqua, GabriellaCocca, EnnioPasqualone, AntonellaCochrane, StephenPasquinucci, LorellaCocovi Solberg, David JaimePasternak, AlexanderCodee, JeroenPasternak, AnnaCoelhoso, IsabelPastey, ManojCohen, YachinPašti, Igor A.Colby, DavidPastor-Anglada, MarçalColdea, TeodoraPastore, ChiaraCole, Kathryn E.Paszkiewicz, SandraCole, NeridaPaszkiewicz-Gawron, MartaCollet, Jean-paulPatel, ALOKCollina, SimonaPatel, DevenCollings, InesPatel, Yashomati M.Collins, HelenPateraki, IriniCollot, MayeulPathakoti, KavithaColmenares, Juan CarlosPatil, Ajay BhagwanColniță, AliaPatil, RaulColobatiu, LioraPatra, AmiyaColognesi, DanielePatra, NiranjanColombo, DiegoPatrizia, NittiColon Saez, JosePatruno, AntoniaČolović, Mirjana B.Pattabiraman, MaheshColuccia, MauroPattammattel, AjithColussi, AgustinPatterson, JenniferCombès, AudreyPatyra, EwelinaComito, Robert J.Paudel, AmritCompare, DeboraPaul, Narayan ChandraConcetta Valeria Lucia, GiosafattoPaulmurugan, RamasamyConcilio, SimonaPavao, Mauro SergioConforti, FilomenaPavela, RomanCongestri, RobertaPavelić, KrešimirConsole, LaraPavese, AlessandroConsonni, RobertoPavic, ValentinaConstantinou, ViolettaPavle, JovanovConte, LanfrancoPavlov, DanailConti, BarbaraPavlov, Youri IContiero, JonasPawar, PrashantContini, AlessandroPawełczyk, AnnaCopolovici, Dana MariaPawłat, JoannaCoppedè, FabioPawlicki, MiłoszCorassin, CarlosPawlik, AnnaCorazzin, MircoPawluć, PiotrCorchete, PurificacionPayton, JohnCordani, MarcoPazdro, RobertCordaro, MarikaPazourek, JiriCordero, Chiara EmiliaPeana, Massimiliano F.Corfield, Anthony P.Pechkova, EugeniaCorke, HaroldPecorini, IsabellaCornejo, JavieraPeczuh, MarkCornett, ClausPedatella, SilvanaCornilescu, GabrielPedersen, Steen BønløkkeCornu, AgnesPedraza Chaverri, JoséCorrea, ArkaitzPedro, José R.Corrêa, Arlene GonçalvesPedrosa, RafaelCorrea, Daniel S.Peiretti, Pier GiorgioCorrea-Basurto, JosePeixoto, Philippe AlexandreCorreia, Ana CristinaPejin, BorisCorreia, João D. G.Pekar, MiloslavCorreia, ManuelaPelegov, DmitryCorsaro, CarmeloPelin, MarcoCortes, NataliePellei, MauraCortijo, MiguelPellis, AlessandroCorvo, Marta C.P.C.Peluso, IlariaCosentino, MarcoPena-Pereira, FranciscoCosma, PinalysaPence, BrandtCosme, FernandaPeng, Wen-HuangCossignani, LinaPeng, ZhihongCossío, UnaiPeng, ZhiliCosta, Carlos MiguelPengo, PaoloCosta, Elisabete V.Penissi, Alicia B.Costa, GeisonPeppelenbosch, M.P.Costa, GiosuèPeptu, CristianCosta, Paulo JorgePeralta, RosaneCosta, PedroPercino, JudithCosta, Vera MarisaPercival, Susan S.Costantini, SusanPerdih, AndrejCostantino, UmbertoPereira, Alice S.Costas, MiguelPereira, AnaCostisor, OtiliaPereira, CarlaCotella, DiegoPereira, DavidCouce, María D.Pereira, FlorbelaCouderc, FrançoisPereira, JorgeCourtois, GillesPereira, José AugustoCoutinho, Henrique Douglas MeloPereira, LeonelCoutinho, Paulo José GomesPereira, Maria De LourdesCova, TâniaPereira, Olívia R.Covino, RobertoPereira, RubenCoy-Barrera, EricssonPereira, VandaCoyne, MarkPereira-Maia, EleneCozzolino, Anthony F.Perelomov, Leonid V.Cozzolino, DanielPeres, António M.Cragg, PeterPerestrelo, RosaCravero, Maria CarlaPerestrelo, Rosa Maria De SáCremades Ugarte, JavierPérez Alvarez, Maria JoséCrespo, IrenePerez Silanés, SilviaCrespo-Hernández, CarlosPérez Trujillo, MíriamCretoiu, DragosPérez, Ana G.Crisafi, FrancescaPérez, AndyCrișan, GianinaPérez, ElíasCrisan, LuminitaPerez, MartaCrisponi, GuidoPérez-Badia, RosaCroce, Anna CletaPérez-Casas, SilviaCrucho, Carina Isabel CorreiaPérez-Fernández, MaríaCrupi, PasqualePérez-Gálvez, AntonioCrus-Topete, DianaPérez-García, SeleneCruz, CarlaPérez-Lamela, ConcepciónCruz, Rui M.S.Pérez-Villanueva, JaimeCruz, SandraPeri, FrancescoCruz-Lopez, OlgaPerinelli, Diego RomanoCruz-Sosa, F.Perino, SandrineCsámpai, AntalPerissutti, BeatriceCsősz, ÉvaPeriyasamy, PalsamyCsuk, RenéPerlepes, SpyrosCsupor, DezsőPerlepes, Spyros P.Cucinotta, FabioPerles, JosefinaCuculić, VladoPermyakov, EugeneCuerva, Juan M.Perna, FilippoCui, HuaqingPerna, GiuseppeCui, ZhibinPerni, MicheleCumming, Adam SPernyeszi, TímeaCummins, Nathan W.Peron, GregorioCunha, SaraPerrone, GiancarloCuny, Gregory D.Perry, RachelCuomo, FrancescaPescitelli, GennaroCupellini, LorenzoPešić, MilicaCuperlovic-Culf, MiroslavaPessela João, BenevidesCurti, ClaudioPestov, AlexanderCurtis, Anthony D.M.Peter, AncaCvrček, LadislavPéter, AntalCybinska, JoannaPeter, Emanuel K.Cybulska, IwonaPeter, FranciscCygorek, MoritzPeter, KatherineCyplik, PawełPeters, ChristinCzaplewski, CezaryPeters, VerenaCzarnocki, ZbigniewPeterson, BrandonCzekala, WojciechPeterson, LarrynCzekelius, ConstantinPetit, Patrice X.Czernek, JiříPetkova, NadezhdaCzerwińska, EwaPetrakis, EleftheriosCzerwinska, MonikaPetrásková, LucieCzerwińska, MonikaPetrella, AndreaCzigany, ZsoltPetriccione, MilenaCzogalla, AleksanderPetricevich, VeraCzosnek, CezaryPetrie, Paul R.D’Acquarica, IlariaPetrikaite, VilmaD’Agostino, PaulPetroianu, GeorgD’Alessandro, GiuseppinaPetroni, KatiaD’Ambrosio, MichelePetropoulos, SpyridonD’Anna, FrancescaPetropoulos, Spyridon A.D’Anneo, AntonellaPetrou, Athinoula L.D’Archivio, Angelo AntonioPetrou, ChristosD’Auria, MaurizioPetrov, DmitryD’Errico, StefanoPetrov, DrazenD’Orazio, GiovanniPetrov, PanayotD’Urso, AlessandroPetrova, Krasimira T.Da Cunha, Mário Antônio AlvesPetrova, MarinaDa Rosa, MarceloPetrović, BiljanaDa Silva Júnior, Eufrânio N.Petrovski, ŽeljkoDa Silva Pena, RosinelsonPetrucci, RitaDa Silva, João FerreiraPetruczynik, AnnaDa Silva, José P.PEYRON, Jean-FrançoisDa Silva, Luís C. N.Peyrottes, SuzanneDa Silva, Luis Cláudio NascimentoPeyton, David H.Da Veiga Junior, Valdir FlorencioPezeshki, AtiyeDacarro, GiacomoPfattner, RaphaelDagmara, MalinaPhan, Chin-SoonDahiya, RajivPhilipp, ManfredDahl, Jan-UlrikPhilips, NeenaDahoumane, Si AmarPhillips, JoshuaDai, WeilinPiao, Hai-longDai, YuminPiao, XijunDai, ZhaohuaPiasecka, AnnaDai, ZiweiPiatkevich, KirylDaiguebonne, CarolePiccialli, GennaroDal Ben, DiegoPiccialli, VincenzoDal Ben, MatteoPiccinelli, FabioDall’Acqua, StefanoPiccolella, SimonaDall’Olio, FabioPichler, VerenaDallavalle, SabrinaPicone, DeliaDallemagne, PatrickPidot, Sacha J.Dalmora, Sérgio LuizPieczywek, Piotr M.Dalpozzo, RenatoPiel, MarkusDalzell, JohnathanPielech-Przybylska, KatarzynaDamalanka, VishnuPielichowska, KingaDambolena, José S.Pielichowski, KrzysztofDamkaci, FehmiPiemontese, LucaDamma, DevaiahPiepenbrink, KurtDams-Kozlowska, HannaPiermatti, OrianaDanciu, CorinaPierre, GuillaumeDandawate, PrasadPieters, RolandDaneshgar, SabaPietrasik, JoannaDanezis, GeorgePietrella, DonatellaDangol, ManitaPietropolli Charmet, AndreaDaniel-da-Silva, AnaPietzsch, Hans-JürgenDaniellou, RichardPifferi, ValentinaDanielson, NeilPigge, F. ChristopherDanijela, BognerPignitter, MarcDanikiewicz, WitoldPike, Nicholas A.Danjou, Pierre-EdouardPillaiyar, ThanigaimalaiDanko, MartinPilo-Pais, MauricioDansette, Patrick M.Piluso, SusannaDarabantu, MirceaPina, Maria PilarDaranas, Antonio HernándezPinalli, RobertaDarbani, BehroozPinar, AnitaDarcy, JustinPineda, MiguelDardenne, LaurentPinedo-Rivilla, CristinaDas, PrasantaPiñeiro, MartaDasgupta, SrimoyeePinela, JoséDasgupta, SubhamPinheiro, JoaquimDash, RanjeetPinheiro, MarinaDátka, JerzyPinho, SimãoDaugelavicius, RimantasPini, ElenaDaverey, AmitaPinkas, JiriDavid, Iulia GabrielaPintea, AdelaDavidchack, Ruslan LPinteala, MarianaDavioud-Charvet, ElisabethPinter, TylerDavis, Rohan A.Pinto Da Silva, LuísDawson, James A.Pinto, Diana CláudiaDaygon, DaraPinto, Ernanide Aguiar Pertinhez, ThelmaPinto, EugéniaDe Almeida, AndreiaPinto, SandraDe Alvarenga, Elson SantiagoPinzaru, IuliaDe Araújo, Daniele RibeiroPinzi, LucaDe B. Harrington, PeterPiosik, JacekDe Barros Fernandes, PedroPiotrowska, MałgorzataDe Bellis, LuigiPiotrowski, PaulinaDe Benedetto, GiuseppePiper, BrianDe Castro Ramalho, TeodoricoPiper, Peter W.De Castro, SoniaPiquereau, JérômeDe Ceballos, Maria L.Pires, José CarlosDe Deurwaerdere, PhilippePires, Regina HelenaDe Diego, MartaPiret, JocelyneDe Falco, EnricaPirola, LucianoDe Feo, VincenzoPisapia, PasqualeDe Giorgi, Maria LuisaPisarcik, MartinDe Gonzalo, GonzaloPisklak, Dariusz MaciejDe Graaf, CoenPissarek, MargitDe Jesus Raposo, Maria FilomenaPitsikalis, MarinosDe Jesús Ruíz-Baltazar, ÁlvaroPitterl, FlorianDe La Fuente, CesarPitto, LetiziaDe La Garza, MireyaPitto-Barry, AnaïsDe La Hoz, AntonioPitucha, MonikaDe La Lastra, José Manuel PérezPiunova, VictoriaDe La Puerta, RocioPiva, RobertoDe la Torre, Beatriz G.Piwowar, AgnieszkaDe La Torre, Maria Del CarmenPixley, SarahDe La Vega, José Manuel GarcíaPizzo, ElioDe Las Heras, BeatrizPlácido-Escobar, Jersson E.De León, A. S.Plader, WojciechDe Lima, RenataPlastina, PierluigiDe Luca, Maria AntoniettaPlatts, JamesDe Luca, MichelePlatts, James A.De Luca, PierantonioPlavec, JanezDe Martin, SaraPłaza, GrażynaDe Masi, LuigiPlaza-Díaz, JulioDe Mendonça, Dina I. M. D.Plech, TomaszDe Mieri, MariaPlessas, StavrosDe Miranda, João Antônio LealPletnev, SergeiDe Oliveira, Eduardo BasílioPlohl, OlivijaDe Oliveira, José Miguel P. FerreiraPłotka-Wasylka, JustynaDe Oliveira, Kléber T.Plotnikov, Egor Y.De Oliveira, Luiz Fernando C.Pluhařová, EvaDe Oliveira, Marcos RobertoPluta, RyszardDe Palma, MichelePluth, Michael D.De Pasquale, ClaudioPlymale, AndrewDe Paz, Antonio TaberneroPoad, BerwyckDe Paz, Jose LuisPocheć, EwaDe Pinto, VitoPoddel’sky, AndreyDe Riccardis, M. FedericaPodlewska, SabinaDe Robertis, MariangelaPodsiadły, RadosławDe Santi, MauroPoggi, AlessandroDe Sousa, Frederico B.Pogorzelska, AnetaDe Sousa, Maria João De Almeida CoelhoPohl, EhmkeDe Souza Gil, EricPohl, PawelDe Tommasi, NunziatinaPohl, SebastianDe Vendittis, EmmanuelePohnert, GeorgDe Villiers, MelgardtPoinsot, VerenaDe Vleeschouwer, FreijaPoletto, MatheusDe Vries, MattanjahPolge, CécileDe Vries, SjoerdPoli, GiulioDe Vrieze, ErikPolishchuk, PavelDe Winter, HansPolishuk, IlyaDe Zotti, MartaPolitakos, NikolaosDea-Ayuela, María AuxiliadoraPoliteo, OliveraDeacon, GlenPollastro, StefaniaDeagostino, AnnamariaPolonini, Hudson CaetanoDebiec-Andrzejewska, KlaudiaPolverino De Laureto, PatriziaDębowski, DawidPolyak, StevenDedova, TatjanaPolyakov, NikolayDeffïeux, DenisPoma, PaolaDegennaro, LeonardoPomastowski, Paweł PiotrDegiacomi, Matteo T.Pomerantz, WilliamDegl’innocenti, DonatellaPomin, VitorDegler, DavidPons, JosefinaDegola, FrancescaPonti, AlessandroDehghan, EsmaeilPoon, GregoryDeilmann, ThorstenPoór, MiklósDeineka, VictorPop, OanaDeive, FranciscoPopa, Daniela-SavetaDeja, StanislawPopa, LacramioaraDejeu, JérômePopiołek, ŁukaszDel Caro, AlessandraPopova, Milena P.Del Castillo, Roxana MitzayéPopović-Djordjević, JelenaDel Giudice, RitaPopovich, DavidDel Pozo Bayón, Maria A.Popp, DennyDel Río Celestino, MercedesPoppe, LászlóDel Rio, GabrielPorat, Ze’evDel Valle, JuanPorcelli, FernandoDelalande, OlivierPorollo, AlekseyDelaney, ColmPorro, ChiaraDelattre, CédricPortugal, Camila C.Delaude, LionelPoŝa, MihaljDelbarre Ladrat, ChristinePospisil, JiriDelbarre-Ladrat, ChristinePossik, EliteDelbecq, FrédéricPostigo, AlDelfino, Domenico V.Postila, Pekka A.Dell’Agli, MarioPostnikov, PavelDell’Anna, Maria MichelaPotaczek, Daniel P.Della Ca’, NicolaPotapov, AndreiDella Sala, GiorgioPotgieter-Vermaak, SanjaDellaGreca, MarinaPothupitiya, JinalDelogu, GiovannaPotterat, OlivierDelorme, VincentPoudel, Barunde-los-Santos-Álvarez, NoemíPouli, NicoleDemarquoy, JeanPoupot, RemyDembska, Anna RenataPourcet, BenoitDemel, JanPower, AoifeDemerdash, Omar N.A.Powers, David C.Demey, HaryPowers, RobertDe-Miguel, ManuelPozsgai, GaborDemir, SelvanPozzi, CeciliaDemopoulos, Vassilis J.Prabakar, Sujayden Hertog, JeroenPradoMassarioli, AdnaDeng, JianjunPragliola, StefaniaDeng, NanjiePrajzler, VaclavDeng, Wei-WeiPrakash, AishwaryaDenis, SerenoPrandi, CristinaDenkov, NikolaiPrasad, BhagwatDenny, BillPratsinis, HarrisDeokar, HemantkumarPraus, PetrDerat, EtiennePravetoni, MarcoDerbré, SéverinePreethi, Korangath ChandranDerewiaka, DorotaPreiner, DarkoDerler, IsabellaPreller, MatthiasDesage-El Murr, MarinePremasiri, R. RanjithDesai, Umesh R.Prester, LjerkaDeska, JanPriebbenow, DanielDessent, CarolinePrieto Vidal, NataliaDessolin, JeanPrieto-Lloret, JesusDeStefano, JefferyPrimožič, InesDevasurendra, AmilaPrnová, AnnaDevkota, Hari PrasadProchazka, MarekDey, GangotriProestos, CharalamposDeyrup, StephenProrokova, Natalia PetrovnaDhakal, DipeshProsen, HelenaDhakal, SagarProshlyakov, Denis A.Dhakshinamoorthy, AmarajothiProtti, MicheleDhaliwal, GurjotProvazník, IvoDhar, ArunPruna, Alina IulianaDharavath, SrinivasPrzybył, Jarosław L.Dhaundiyal, AlokPrzybyłek, MaciejDi Bella, SantoPrzybyło, MałgorzataDi Buduo, ChristianPrzybylski, PiotrDi Bussolo, ValeriaPrzybyt, MałgorzataDi Cesare Mannelli, LorenzoPrzysławski, JuliuszDi Ciccio, PierluigiPsaroudakis, NikolaosDi Donato, PaolaPtaszek, MarcinDi Donna, LeonardoPu, JingDi Giacomo, SilviaPu, Ying-ChihDi Gioia, Maria LuisaPuca, AnnibaleDi Gioia, SantePucci, AnnemarieDi Girolamo, RoccoPucheault, MathieuDi Lorenzo, FrancescoPuchot, LauraDi Loreto, SilviaPuddephatt, RichardDi Luccia, AldoPuertas-Mejía, Miguel ADi Maio, LucianoPuga, Alberto V.Di Micco, SimonePuglisi, AlessandraDi Natale, ConcettaPuizina, JasnaDi Profio, PietroPula, GiordanoDi Sotto, AntonellaPullman, David P.Di Stefano, AntonioPulvirenti, AndreaDi Zazzo, ErikaPunta, CarloDi, RongPushkar, YuliaDiaba, FaïzaPuszka, AndrzejDiaconeasa, ZoritaPuttreddy, RakeshDiana, RositaPycia, KarolinaDias Soeiro Cordeiro, Maria NataliaPyka, AlinaDias, DaianePyka-Pająk, AlinaDias, JulianaPyta, KrystianDíaz, CristinaQaim, Syed M.Diaz, GasparQian, ChunqiDíaz, JesúsQian, Jin-yuanDíaz, LorenzaQian, MingxingDíaz, María TeresaQiang, ZheDiaz-Marrero, Ana R.Qin, HuaDíaz-Ortiz, ÁngelQin, Hua-LiDiban, NazelyQin, SanboDickinson, SallyQin, XiaoliDidier, PascalQing, Lin-senDie, JoseQiu, BinDiederich, MarcQiu, FengDiesendruck, CharlesQiu, HongdengDíez, DavidQiu, JianmingDiliën, HanneQiu, MinghuaDill, VeronikaQiu, ZhenDilman, AlexanderQneibi, MohammadDimas, KonstantinosQu, XiaogangDimesso, LucangeloQu, XiaozhongDimkić, IvicaQuadrelli, PaoloDimmock, Jonathan R.Quan, Chun-shanDimova, ElitsaQuan, GaofengDinadayalane, TandabanyQuan, Zhe-ShanDing, BaoqingQuartu, MarinaDing, ChunbangQueiroz Monteiro, RobsonDing, JianxunQueneau, YvesDing, QingbaoQuiles Morales, Jose L.Ding, Wei DanQuiliano, MiguelDing, YousongQuinn, Mark T.Ding, ZhaoyangQuintard, Jean-PaulDinic, JelenaQuintas, Luis Eduardo M.Dinica, Rodica MihaelaQuintavalla, AriannaDinis, Lia Tânia JQuirino, Joselito P.Dinischiotu, AncaQuodbach, JulianDisney, Matthew D.R. El-Seedi, HeshamDittmar, ThomasR. Odell, LukeDjobo, Jean Noël YankwaR. Santos, Cleydson BrenoDludla, Phiwayinkosi V.Raasmaja, AtsoDmitrenok, Pavel S.Rabanal Anglada, FrancescDo Carmo, Sergio J. C.Rabbani, GulamDobosz, BernadetaRabiee, HesamoddinDobričić, VladimirRabot, AmélieDobrovetsky, RomanRachwalski, MichałDobrowolski, PiotrRac-Rumijowska, OlgaDobson, HeidiRácz, AnitaDoci, ColleenRadaram, BhaskerDoda, Sai ReddyRadchenko, Eugene V.Doddapattar, PrakashRadchenko, ValeryDodero, VeronicaRademacher, ChristophDodi, GianinaRadenković, SlavkoDoerksen, Robert J.Rádis-Baptista, GandhiDohi, ToshifumiRadisky, EvetteDolashka, PavlinaRadko, LidiaDolezal, MartinRadmila, PavlovicDolganov, Pavel VladimirovichRadonic, VasaDolgova, OlgaRadosavljević, TatjanaDoligalska, MariaRadtke, AleksandraDołowy, MałgorzataRadu, Gabriel LucianDolzhenko, Anton V.Radulescu, CristianaDomagała, MałgorzataRadušienė, JolitaDomenico, GabrieleRadwan, MohamedDomine, Marcelo E.Radzymińska-Lenarcik, ElżbietaDominguez, RubenRafael, DianaDomínguez-Álvarez, EnriqueRaffa, DemetrioDomínguez-Perles, RaúlRafińska, KatarzynaDomitrović, RobertRaftopoulos, KonstantinosDon, Aakash WelgamageRagain, ChristinaDonato, Rosario FrancescoRagg, EnzioDong, ChangkunRagno, GaetanoDong, HaiRagno, RinoDong, RenhaoRagupathy, SubramanyamDong, ShihuiRagusa, AndreaDong, YueshengRaguso, RobertDormond, OlivierRahimi, FaridDorozhkin, Sergey V.Rahimi, MohammadDos Santos Lima, MarcosRahimi, ParvanehDos Santos Niculau, EdenilsonRahman Pour, RahmanDos Santos, CatarinaRahman, AzizurDos Santos, Demetrio JacksonRahman, MasudurDos Santos, Jean LeandroRahman, Md ToufiqurDos Santos, José C.S.Rahme, KamilDos Santos, Reinaldo SousaRaimondi, LauraDos Santos-García, Antonio JuanRaimondi, Maria ValeriaDoseff, Andrea I.Raimondi, StefanoDosoky, NouraRaimondo, StefaniaDostalek, PavelRaimundo, NunoDotsikas, YannisRaj, Madhwa H.G.Dou, JinboRajauria, GauravDou, LaetitiaRajčević, NemanjaDou, Q. PingRaje, MithunDouda, OndřejRajić, ZrinkaDoupnik, Craig A.Ralston, EmilyDoyen, JérômeRamamurthy, VaidhyanathanDrabińska, NataliaRaman, VenuDrabowicz, JozefRamaraj, PanduranganDračínský, MartinRamasamy, ElamparuthiDragan, Ecaterina StelaRamasamy, MohankandhasamyDraghi, LorenzaRamil, CarloDragos, HorvathRamírez, MartínDragutan, ValerianRamón, Diego J.Drąg-Zalesińska, MałgorzataRamona, PaltineanDrew, MichaelRamos, CatarinaDridi, SamiRamos, Marco A.Drioli, EnricoRamos, SoniaDrobizhev, MikhailRamos-Jiménez, ArnulfoDrozdov, Aleksey D.Ramos-Organillo, ÁngelDrutovic, DavidRamos-Ortiz, GabrielDu Plessis, Heinrich WRan, YanDu, ChangwenRanadheera, Chaminda SenakaDu, DeguoRanc, VáclavDu, GuodongRandazzo, Giuseppe MarcoDu, MingRangnekar, AbhijitDu, Shu-shanRanjan, AlokDu, XingrongRanjit, SumanDu, YanmingRanjkesh, AmidDu, YongleRankin, Rees B.Du, Yong-ZhongRanzato, EliaDu, ZhentingRao, FaizaDuan, LianRapak, AndrzejDuarte, AnaRapeanu, GabrielaDuarte, NoeliaRaposio, EdoardoDubey, NeeruRaposo, Maria ManuelaDucancel, FrédéricRapposelli, SimonaDudas, JozsefRasmussen, Lars HolmDudás, ZoltánRaspanti, MarioDudding, TravisRassidakis, GeorgiosDudek, KatarzynaRastija, VesnaDudhipala, NarendarRatajczak, EwelinaDuefer, MartinaRateb, MostafaDufossé, LaurentRateb, Mostafa E.Dugé De Bernonville, ThomasRathousky, JiriDuggan, Brendan M.Ratković, Zoran R.Duh, Chang-YihRatner, AlbertDulski, MateuszRattray, Nicholas J. W.Dumbrava, AncaRaudonė, LinaDumetz, FranckRault, FrançoisDunietz, Barry D.Rault-Berthelot, JoëlleDuong, Hai MinhRaut, PrasadDurand, Jean OlivierRauwel, ProtimaDurand, Jean-OlivierRaval, YashDurán-Peña, María JesúsRavan, MaryamDurazzo, AlessandraRavelli, DavideDurgo, KsenijaRavera, MauroĐurović, SašaRavula, SudhirDussault, PatrickRawel, HarshadraiDuszczyk, JakubRawson, JeremyDutartre, PatrickRay, Partha (Moores Cancer Center)Dutkiewicz, MichałRay, Partha (University of Reading)Dutta, Kingshukrayan, anwarDutta, PalashRayson, Gary D.Duttaroy, Asim K.Realinho, VeraDuval, Raphaël E.Rebelo, Sofia P.Duzgunes, NejatReber, ChristianDvořáčková, MartinaRecabarren Gajardo, GonzaloDydio, PawełReddy, SakamuriDymarska, MonikaReddy, Sudhir PuttyDymińska, LucynaReed, DanielDžamić, AnaReed, ScottDziadas, MariuszRega, NadiaDzida, MarzenaRegad, TarikDzidic, AlenRegal, PatriciaDziedzic, ArkadiuszRegazzoni, Luca GiovanniDziegiel, PiotrRegel-Rosocka, MagdalenaDziki, DariuszRegiec, AndrzejDzimitrowicz, AnnaRegmi, BishnuDziubek, KamilReguero, MarDziurka, MichalRegulska, ElzbietaDziurkowska, EwelinaRehrig, Erin M.Dzolic, ZoranReid, Christopher W.Dżugan, MałgorzataReid, ScottEaton, GarethReigosa, Roger J.Ebani, Valentina VirginiaReimann, Regina R.Ebeler, SusanRein, TheoEberlin, LiviaReis, Catarina PintoEbert, Timothy A.Reis, Marco S.Ebrahim, WeaamReischl, RolandEbrahimi, AminReiterman, PavelEconomou, VangelisRemenyik, JuditEdafiogho, IvanRemesar, XavierEddy, NicholasRemiao, Fernando M.G.Edin, Nina JeppesenRen, Di-FengEdina, PallagiRen, DingkunEdkins, RobertRen, QingzhongEdmiston, PaulRen, Yuan (David)Edreira, Martin M.Ren, YulinEdström, DanielRenault, NicolasEfferth, ThomasRenčiuk, DanielEfimov, AlexanderRendueles, ManuelEfird, Jimmy T.Renna, MassimilianoEfremenko, ElenaRenner, UlrichEgami, HiromichiReparaz, SebastianEgawa, GyoheiRequicha, JoãoEgea Gutiérrez-Cortines, MarcosRescifina, AntonioEgorkin, VladimirRescigno, AntonioEhlers, PeterResende, Jarbas M.Ehm, ChristianRety, StephaneEhrlich, GarthRevilla, EugenioEid, NabilRevuelta, JuliaEijkelkamp, BartReyer, HenryEisenreich, WolfgangReyes Parada, MiguelEjfler, JolantaReyes, ArchieEjidike, Ikechukwu P.Reyes, FernandoEkaterina, BartashevichReygaert, WandaEkielski, AdamReynisson FRSC, JóhannesEkiert, HalinaReynisson, JóhannesEklund, Patrik C.Rhee, Hyun-WooEl Kalamouni, ChakerRhee, Jin-KyuEl Sayed, SamehRho, Jung-RaeElansary, Hosam O.Ribaudo, GiovanniEl-Dars, FaridaRibeiro, ClaudiaElder, RhoderickRibeiro, DanielaEleftheriadis, TheodorosRibeiro, Helena MargaridaElemans, J.A.A.W.Ribeiro, JoãoELFALLEH, WalidRibeiro, Luís FilipeElgendy, MohamedRibeiro, Maria H. L.Elia, AntonioRiccardi, ClaudiaElkins, KellyRicci, AriannaEllett, FelixRicci, MarilenaElnashar, Magdy M.Riccò, RaffaeleEl-Nezami, Hani SaidRice, Peter J.El-Seedi, HeshamRicelli, AlessandraElshafie, Hazem S.Richard, PeterElshahawi, SherifRichards, ChrisElsinghorst, Paul W.Richardson, Rudy J.El-Zaria, MohamedRichichi, BarbaraEmani, ChandrakanthRichtera, LukasEmmer, AdamRichtera, LukášEnami, ShinichiRiedel, ChristianeEnchev, VenelinRiess, GisbertEndo, HarukaRigano, DanielaEngelberth, JurgenRighetti, LauraEngle, Keary M.Rijo, PatríciaEnglert, UlliRinaldi, AlessandraEnglezos, VasileiosRío Segade, SusanaEnjapoori, Ashwantha KumarRios, RamonEnkvist, ErkiRipperger, Jürgen A.Enríquez Pérez, EstherRisinger, April L.Eppard, ElisabethRitter, Christoph A.Erbe, ArturRitzoulis, ChristosEremeyev, VictorRivera De La Rosa, JavierEritja, RamonRivera-Monroy, Zuly JennyErnst, Oliver P.Rivero-Cruz, J. FaustoEroğlu, AbdulkerimRivero-Müller, AdolfoErrington, Timothy M.Rivero-Pérez, M. DoloresErsan, SevcanRizzo, CarmenErwan, PaineauRizzo, MilenaEsatbeyoglu, TubaRizzolio, FlavioEscalante, AnayansiRizzolo, AnnaEscalante, CarlosRizzuti, BrunoEscalante, JaimeRo, Hyeon-SuEscames, GermaineRobert, PazEscárcega Bobadilla, Martha VerónicaRoberto Da Silva, Dr. EdsonEscolà-Gil, Joan CarlesRoberto, Sergio RuffoEscolano, CarmenRoberts, Arthur G.Escorihuela, JorgeRobertson, Avril A. B.Escudero, AnaRobertson, Mark J.Escudero, OlgaRobien, WolfgangEskin, MichaelRobiette, RaphaelEspada-Bellido, EstrellaRobinson, Samuel D.Espindola, LailaRobledo, Sara M.Espindola, Laila SalmenRobledo, Virginia RodríguezEspinosa-Andrews, HugoRobles, EduardoEspinoza, JavierRoca, MartaEspinoza, LuisRoca-Sanjuán, DanielEspita, PaulaRocca, RobertaEspitia-Pinzón, Clara InesRocchetti, GabrieleEsposito, Carla LuciaRocha Filho, Pedro Alves daEsposito, SergioRocha, HugoEspro, ClaudiaRocha, JavierEsquivel, BaldomeroRochais, ChristopheEstanis, NavarroRocha-Martin, JavierEsteve-Romero, JosepRocio-Bautista, PriscillaEstévez Cabanas, Ramón J.Rodell, ChristopherEstévez, JorgeRodolfo, CarloEstevinho, Leticia M.Rodrigo Saez, LuisEstour, FrançoisRodrigues, António S.Etinski, MihajloRodrigues, Célia F.Ettari, RobertaRodrigues, FernandaEtxebarria, N.Rodrigues, FranciscaEudes, AymerickRodrigues, JoanaEugenio, ApreaRodrigues, JoaoEugenio, María E.Rodrigues, Joaquim RuiEuverink, Gert-JanRodrigues, MárcioEvanno, LaurentRodrigues, RafaelEvans, MarleneRodrigues, RaquelEvtugyn, GennadyRodríguez García, Mario E.Ewald, CollinRodríguez Romero, AdelaEyckens, Daniel J.Rodríguez Villanueva, JavierEyer, LuděkRodríguez, Gustavo RubénEyres, GrahamRodriguez, Hortensia M.Ezquerra-Brauer, Josafat-MarinaRodríguez, IsaacEzzati Nazhad Dolatabadi, JafarRodríguez, JaimeEzzeddine, ZeinabRodríguez, José AntonioFabian, ZsoltRodriguez, Juan B.Fabiszewska, AgataRodríguez, LauraFacão, JorgeRodríguez, MarioFadda, AngelaRodríguez-Flores, M ShantalFagone, PaoloRodríguez-Gómez, ArturoFahey, JedRodríguez-Henche, NievesFahrenbach, AlbertRodríguez-Jimenes, GuadalupeFaisal, Shaikh NayeemRodríguez-Llorente, Ignacio DavidFalanga, AnnaritaRodríguez-Lorenzo, LuisFalcão, Soraia I.Rodríguez-Roque, María JanethFalconer, JamesRodríguez-Solana, RaquelFaleiro, Maria LeonorRodríguez-Yoldi, María JesúsFalqué López, ElenaRodziewicz, PawelFalzone, LucaRoglans, AnnaFamiani, FrancoRogobete, Alexandru FlorinFan, ChenRogozhin, EugeneFan, HaimingRoh, ChanghyunFan, Huan-JungRoh, JaroslavFan, JianglinRohacs, TiborFan, MingtaoRohlena, JakubFan, Tai-HsiRohn, SaschaFan, WeizhengRoiland, ClaireFan, ZhijinRojas-Aguirre, YareliFan, ZhiqiangRojo, Maria AngelesFanali, ChiaraRokita, BożenaFanali, SalvatoreRoll, MarkFanfrlik, JindrichRomán Guerrero, AngélicaFanfrlík, JindřichRoman, AndresFang, ChengRomani, AndreaFang, JimRoman-Junior, WalterFang, LijingRomán-Martínez, M.C.Fang, MarongRomano, GiovannaFang, MingxiRomano, NiclaFang, YiRomanov, Georgy A.Fanizzi, Francesco PaoloRomanowska-Duda, ZdzisławaFantacuzzi, MarialuigiaRomanucci, ValeriaFantini, SebastianRoma-Rodrigues, CatarinaFarantos, Stavros C.Romeo, RobertoFaraone Mennella, Maria RosariaRomero, Aldo HumbertoFarcas, Anca CorinaRomero, JulianFarcasanu, Ileana C.Romero-Enrique, Jose M.Fare, SilviaRomero-García, Juan M.Fargnoli, MarioRomero-Muñiz, CarlosFarhat, GraceRomero-Puertas, María C.Farjas, JordiRomero-Salguero, Francisco J.Farka, ZdeněkRonca, AlfredoFarkaš, PavolRondeau, PhilippeFarneti, BrianRonsisvalle, SimoneFarone, Mary B.Root, MartinFarsa, OldřichRoper, Randall J.Fasullo, MichaelRopka-Molik, KatarzynaFatouros, Dimitrios G.Rosa, Ambra R. DiFatyeyeva, KaterynaRosado, CatarinaFaulconer, Emily K.Rosas, Elaine CruzFavreau, Peter F.Rosato, AntonioFayet, GuillaumeRoşca, Sorin-ClaudiuFeczkó, TivadarRoscini, LucaFederico, StephanieRosenberg, ErwinFedorova, Olga S.Rosenberg, IvanFedorova, Olga V.Rosenberg, MosheFedriani, Luis EsquiviasRosenberry, TerroneFeher, MiklosRosencrantz, Ruben R.Fei, TongRosi, MarzioFelczak, KrzysztofRosicka-Kaczmarek, JustynaFeldblyum, Jeremy I.Ross, IanFelpin, François-XavierRoss, MatthewFenaille, FrancoisRossella, FrancescoFeng, Chia-HsienRossi, DanielaFeng, ChuanpingRossi, FilomenaFeng, NingguoRossi, PaolaFeng, Sheng-WeiRossi, RiccardoFeng, Wei-shengRoszak, SzczepanFeng, WenRoszko, MarekFeng, YongjunRoth, Stephan V.Feng, ZhiweiRothbauer, MarioFenoglio, IvanaRothe, HansjörgFenyvesi, FerencRotondo, ArchimedeFeo, FrancescoRoudier, EmilieFeresin, GabrielaRouge, PierreFerji, KhalidRouhiainen, AriFerlazzo, NadiaRoussel, ChristianFermani, SimonaRoutray, WinnyFernandes, AnaRovero, PauloFernandes, Carla Sofia GarciaRow, Kyung HoFernandes, ChristianRowlands, Gareth J.Fernandes, Elizabeth S.Rowther, Farjana B.Fernandes, Maria José Da SilvaRoy, AnuradhaFernandes, RúbenRoy, DenisFernández Barbero, GerardoRoy, DhruvajyotiFernández Espinar, TeresaRoy, DipankarFernandez, Ana InesRoy, SagarFERNÁNDEZ, ANTONIORoychowdhury, SanjoyFernández, InmaculadaRóżalska, BarbaraFernández, José J.Rozalski, MarcinFernandez, MarioRozhon, WilfriedFernández, MarioRóżyło, RenataFernández-Arévalo, MercedesRozza, ArianeFernández-Ballester, GregorioRtimi, SamiFernández-García, MartaRuan, Chang-ChunFernandez-Lafuente, RobertoRuan, KeFernández-Moreira, VanesaRubert, JosepFernández-Muíño, Miguel AngelRubiolo, PatriziaFerrante, AntonioRubis, BlazejFerrante, ClaudioRubtsov, IgorFerrari, PaolaRudick, JonathanFerraris, DanaRudie, AlanFerraz, Emanuela PradoRudiuk, SergiiFerreira, Ana M.Rudnicka, WiesławaFerreira, JorgeRudnitskaya, AlisaFerreira, MarceloRudolph, ChristianFerreira, Paula M.Rudrawar, SantoshFerrentino, GiovannaRudzińska, MagdalenaFerrer, Maria L.Ruelland, EricFerrer-Gallego, RaulRuggieri, FabrizioFerrer-Gallego, RaúlRuggiero, MichaelFerretti, Anna MariaRugman-Jones, PaulFerrieres, VincentRugman-Jones, Paul F.Ferrigno, AndreaRühl, MartinFerry, LaurentRuiz Molina, DanielFershtat, LeonidRuiz Téllez, TrinidadFesta, Alexey A.Ruiz-Aceituno, LauraFialová, SilviaRuiz-Cruz, SaulFiamoncini, JarleiRuiz-Gómez, GloriaFiaschi, TaniaRuiz-Sánchez, EsaúFidalgo, AlexandraRumyantseva, MarinaFiedot-Toboła, MartaRusakova, IrinaFigiel, AdamRusinek, RobertFigueira, Ana Carolina MiglioriniRusinska-Roszak, DanutaFigueiras, AnaRusmini, PaolaFigueiredo, Ana CristinaRusnati, MarcoFijan, SabinaRussell, TiffanyFilannino, PasqualeRusso Krauss, IreneFilarowski, AleksanderRusso, AlessandraFiligenzi, Michael S.Russo, AnnapinaFilip, GabrielaRusso, DaniloFilipeanu, CatalinRusso, MariaFilipkowska, UrszulaRusso, MariateresaFilippelli, AmeliaRusso, MarinaFilippov, Oleg A.Russo, Mario VincenzoFinelli, CarmineRusso, PatriziaFinne, JukkaRusso, PietroFinney, Eric E.Russu, WadeFiocco, DanielaRuzvidzo, OzinielFiore, Carlos E.Ruzza, PaoloFiori, StefanoRyan, James G.Fiorio, RudineiRybczyńska-Tkaczyk, KamilaFisch, MichaelRybniker, JanFischer, MichaelRydzkowski, TomaszFischer-Fodor, EvaRykowska, IwonaFlamini, GuidoRyu, BoMiFleet, GeorgeRyu, Jae-HaFletcher, Nicola F.Ryu, Seung YoonFlieger, JolantaRyzhov, VictorFlores Johnson, Emmanuel AlejandroRzepecka-Stojko, AnnaFlorescu, MonicaRzymowska, JolantaFlores-Holguín, NormaSaafi, MohamedFloresta, GiuseppeSabater I Serra, RoserFlórez-Fernández, NoeliaSabatier, Jean-MarcFlorowska, AnnaSabatini, ValentinaFochi, MariafrancescaSabatino, DavidFodor, CsabaSabirov, DenisFogacci, FedericaSabol, MajaFogolari, FedericoSacchetti, AlessandroFolgosa, FilipeSacco, PasqualeFonseca, AnaSacks, GavinFontanals, NúriaSackus, AlgirdasFontananova, EnricaSadhasivam, SudharsanFonte, PedroSadkowski, TomaszForli, StefanoSadlej, JoannaFormanek, PavelSadowska, BeataFormanowicz, DorotaSadowska-Krępa, EwaFormato, MarilenaSadykov, VladislavFormisano, CarmenSaez, CarmenFormisano, PietroSafo, Martin K.Fornaguera, CristinaSager, ManfredFornstedt, TorgnySagnou, MarinaForoutan-Nejad, CinaSaha, Manik LalFörsth, MichaelSaha, Sushanta KumarFortenberry, RyanSahoo, Pabitra KumarForteschi, MauroSahu, Ravi P.Forti, LucaSahu, Saura C.Forzato, CristinaSaiano, FilippoFossa, PaolaSaielli, GiacomoFoubelo, FranciscoSaiko, GuennadiFourmigué, MarcSain, SunandaFousteris, ManolisSaini, RupinderFragoso, AlexSaini, ShikhaFraile, AlbertoSaint-Cricq, PhilippeFranca, Eduardo FSaito, AkikoFranceschi, PietroSaito, GenkiFrancesco, StellatoSaito, SatoshiFrancesco, TrapassoSaito, YoshinoriFrancia, SofiaSaitoh, KenjiFrancis, Ashwanth ChristopherSaitz, ClaudioFrančišković-Bilinski, StanislavSaiz-Fernández, IñigoFranco, Carlos M.Sajeva, MaurizioFranco, RicardoSakač, NikolaFrank, EvaSakai, RyuichiFrank, RenéSakamoto, MasamiFrank, SašaSakamoto, ToshioFranko, AndrasSakar, MohanFrankowski, MarcinSakharov, AlexeyFranzoni, GiuliaSakilam, SatishFrasson, IlariaSakkas, HerculesFratila, RalucaSako, YasushiFratini, FilippoSakuma, TetsushiFrattini, DomenicoSakurai, AkihikoFreccero, MauroSal’nikov, Denis S.Frediani, MarcoSalachna, PiotrFredotović, ŽeljanaSalafranca Lázaro, Francisco J.Freitas, Ana CristinaSalaheen, SerajusFreitas, MarisaSalahub, DennisFreitas, Vanessa MoraisSalamanca, Constain H.Freitas, Vera L.S.Salanta, LianaFrelon, SandrineSalazar, Juan RodrigoFresco-Cala, Beatriz MaríaSaldanha, SabitaFrey, MaximilianSaleh, MahmoudFrezza, ClaudioSalehi, Sayed AhmadFries, Pascal H.Salehzadeh-Yazdi, AliFrizzo, Clarissa P.Salerno, MarcoFrkanec, RužaSales, EsterFroehlich, LeopoldSalifoglou, AthanasiosFroldi, GuglielminaSalim, Nora Salina MdFrolov, AndrejSalinas-Rodríguez, EleazarFrolova, LiliyaSalis, AndreaFrontera, Antonio (Universitat de les Illes Balears)Salmas, Ramin EkhteiariFrontera, Antonio (University of Maragheh)Salmeia, Khalifah A.Frontistis, ZachariasSalmerón, IvanFrudakis, GeorgiosSalo-Ahen, OutiFrugis, GiovannaSalobrar-García, ElenaFu, GuopengSalomone, AntonioFu, LiSalonen, LauraFu, PengSalopek-Sondi, BrankaFu, QiangSALUCCI, SARAFu, Shu-LingSalud Bea, Roberto De LaFu, XiaotingSalvador, GilFu, XingSalvador-Reyes, LilibethFuchs, HendrikSalvo, AndreaFuente, ElenaSalzano De Luna, MartinaFuentes, EduardoSamanidou, VictoriaFujii, RitsukoSamara, PinelopiFujii, SatoshiSamborska, KatarzynaFujii, YoshiharuŠamec, DunjaFujii, YukiSamhan-Arias, AlejandroFujimori, KoSami, LakhdarFujisawa, TomotsumiSampaio, Bruno LeiteFujita, MasakiSamperi, FilippoFujita, MikakoSamulionis, VytautasFujita, MorifumiSan Fabian, EmilioFujiwara, TomoyaSanabria-Ríos, David JuliánFukuda, MitsuruSanchez Bornot, Jose MiguelFukuda, TakeshiSánchez Del Pino, Manuel M.Fukumoto, ManabuSánchez, Diego HernánFukuoka, ShuheiSanchez, GoarFuller, AmeliaSánchez, GoarFülöp, FerencSánchez, J. LozanoFülöp, LiviaSánchez, ManuelFunar-Timofei, SimonaSánchez-García, DavidFunes-Ardoiz, IgnacioSanchez-Jimenez, Pedro E.Fuoco, AlessioSánchez-Monedero, MiguelFuoco, TizianaSánchez-Ortiz, AraceliFurdui, BiancaSánchez-Pérez, RaquelFürnsinn, ClemensSánchez-Vergara, María ElenaFürtig, BorisSancho, MatiasFurukawa, TatsuhikoSancineto, LucaFurukawa, YoshiakiSancini, GiulioFuruta, HiroyukiSandhu, AmanFuruta, SaoriSandhu, BhupinderFusi, FabioSandín-España, PilarFuso, FrancescoSandjo, Louis PergaudFutai, MasamitsuSandlers, YanaG. Kozlova, SvetlanaSandulescu, MadalinaGaan, SabyasachiSandusky, Peter OlafGaascht, FrancoisSanganyado, EdmondGabdulkhakov, AzatSangiovanni, EnricoGabelica, ValerieSanina, Nina M.Gabriele, BartoloSanjust, EnricoGabrielson, Nathan P.Sankaranarayanan, Nehru VijiGad, AnnicaSanna, GavinoGadella, Bart M.Sannino, FilomenaGaffet, EricSanoh, SeigoGagaoua, MohammedSansone, AnnaGagat, MaciejSansone, ClementinaGaglione, RosaSansone, FrancescaGagnon, Matthieu G.Sansone, FrancescoGagoś, MariuszSantana, Andrés GonzálezGajdács, MárióSanti, ClaudioGajger, Ivana TlakSantilio, AngelaGalamba Correia, João DomingosSantini, AntonelloGalanakis, CharisSantolla, Maria FrancescaGalan-Vidal, Carlos AndresSantoni, Marie-PierreGalasiti Kankanamalage, AnushkaSantos De Gois, JeffersonGalasso, GiovanniSantos Júnior, Aníbal De FreitasGalbiati, MassimoSantos, AbelGalderisi, UmbertoSantos, ClementinaGaleone, AldoSantos, Eva M.Gales, LuísSantos, Sonia A.O.Galic, AnteSantos, SusanaGalicia-García, TomásSantos-Gomes, GabrielaGalisteo Jr., Andres JimenezSantovito, GianfrancoGall Troselj, KoraljkaSantra, BiswajitGallagher, TravisSantulli, CarloGallardo, EugéniaSantulli, GaetanoGallavardin, ThibaultSanyal, MrinmoyGallego, MartaSanyal, OishiGallina-Toschi, TulliaSanz, CarlosGallo, CarmelaSanz, Mari LuizGallo, MonicaSanz-Marco, AmparoGalloni, PierlucaSapozhnikov, Alexander M.Galus, SabinaSaquet, AdrianoGalvez, JorgeSarabia, FranciscoGalzitskaya, Oxana V.Saraiva, NunoGama, SofiaSaravanakumar, KandasamyGamba-Sánchez, DiegoSardão, VilmaGambetta, Joanna M.Sardella, RoccaldoGambini, JuanŠarić, AnkicaGamble, Donald S.Sarigiannis, YiannisGambuti, AngelitaŠarkanj, BojanGamelas, JoséSarkar, DebanjanGamella Carballo, MariaSarkar, SibajiGámiz-Gracia, LauraSarkar, SujanGan, RenyouSarkies, PeterGan, Ren-YouSarma, SauravGan, YongSarti, ElenaGanesan, PalanivelSartillo-Piscil, FernandoGang, WeiSassi, MohamedGangoli, Varun ShenoySastre, GermanGanguly, AvishekSastre-Santos, AngelaGannett, Peter M.Satake, MasayukiGanta, DeepakSathe, Gajanan JalindarnathGao, BeiŠatínský, DaliborGao, HaoSato, KazunoriGao, HuileSato, KazuyukiGao, JieSato, KousukeGao, Jing-FengSato, KyotakaGao, MinSato, Marcello OtakeGao, WenyunSato, Shunsuke A.Gao, YongfengSato, YusukeGao, ZheSatou, TadaakiGarau, AlessandraSattar, SampurnaGarbayo, InésSaturnino, CarmelaGarcía García, LuisSatyal, PrabodhGarcía, FelipeSaucier, CedricGarcía, Gabriel BlázquezSaul, NadineGarcia, JordiSaurav, KumarGarcia, Jose H.Saurina, JavierGarcía, LucíaSavage, Paul B.Garcia-Bosch, IsaacSavić, AleksandarGarcia-Castello, Esperanza MariaSavić, JelenaGarcia-Deibe, AnaSavina, IrinaGarcia-Gallego, SandraSavy, DavideGarcia-Orduña, PilarSayago, AnaGarcía-Ramos, Juan CarlosSayago, Francisco J.Garcia-Segura, SergiSayydi, NimaGarcia-Serna, JuanSberveglieri, VeronicaGarcia-Sosa, AlfonsoScafato, PatriziaGardeli, ChrysavgiScaini, DenisGargiulo, ValentinaScalici, TommasoGarneau-Tsodikova, SylvieScalvenzi, LauraGarner, AngieScampicchio, MatteoGaro, ElianeScarafoni, AlessioGarozzo, DomenicoScardaci, VittorioGarrett Da Costa, RafaelScarpa, Emanuele-SalvatoreGarrido, Narciso M.Scarso, AlessandroGarrigós, María del CarmenSchachner, JörgGarroni, SebastianoSchaich, KarenGarrote, GilSchaller, HubertGartia, Manas RanjanScharber, MarkusGarvalov, Boyan K.Scheiner, SteveGarzoli, StefaniaSchenone, SilviaGašić, Uroš M.Schepens, BertGáspári, ZoltánScherer Koester, LetíciaGasparri, FabioScheuermann, JörgGasparrini, MassimilianoSchiattarella, AntonioGaston, KirkSchiavon, MichelaGauden, Piotr A.Schiavone, MarcoGaul, DavidSchicho, RudolfGauld, James W.Schiemann, OlavGaur, NishthaSchimmenti, RobertoGautam, SiddharthSchinnerl, JohannGautam, UmaSchirhagl, RomanaGavara, LaurentSchlosser, GittaGavara, RafaelSchmedake, Thomas A.Gaviña, PabloSchmidt, BerndGawel, KingaSchmidt, HeinarGawin, MartaSchmidt, Jonas DamgårdGawlik-Dziki, UrszulaSchmidtke, GunterGazizov, AlmirSchmitz, ChristianGazouli, MariaSchmitz, John C.Gazzano, ElenaSchmutz, MarcGazzano, MassimoSchnabel, ThomasGbylik-Sikorska, MałgorzataSchneekloth, JohnGe, XiaoweiSchneider, BerndGe, XinSchnekenburger, MichaelGebicki, JacekSchoepp-Cothenet, BarbaraGebicki, JanSchoffstall, AllenGeffroy-Rodier, ClaudeSchohn, HervéGelb, MichaelScholkmann, FelixGeletii, YuriiSchöllhorn, BerndGellerman, GaryScholtzová, EvaGellrich, UrsScholz, CarmenGendaszewska-Darmach, EdytaSchönitzer, VeronikaGendek-Kubiak, HannaSchönthal, Axel H.Geng, FangSchopfel, JoachimGeng, TaoSchouten, AlexanderGenovese, AlessandroSchramm, Michael P.Genovese, DamianoSchripsema, JanGenta-Jouve, GrégorySchroeckh, VolkerGentile, FabrizioSchroeder, GrzegorzGentili, AlessandraSchubert, MarioGentilucci, LucaSchuehly, WolfgangGeorgakilas, VasiliosSchughart, KlausGeorgiades, SavvasSchulz, Benjamin L.Georgiev, VasilSchulz, FlorianGérard, StéphaneSchulz, MargotGerasimaitė, RūtaSchulze, JohannesGerbi, VincenzoSchulze, MargitGeremia, SilvanoSchutte, StaceyGergs, UlrichSchutznerova, EvaGeronikaki, AthinaSchwaminger, SebastianGerothanassis, Ioannis P.Schwan, WilliamGerschenson, Lía NoemíSchwarz, PatrickGersen, HenkjanSchwarz, SimonaGervasi, TeresaSchwenzer, BirgitGervasoni, JacopoSchwotzer, MatthiasGeszke-Moritz, MałgorzataŚcianowski, JacekGhadwal, RajendraŚcibisz, IwonaGhag, GauravSciortino, GiuseppeGhanem, AshrafScipioni, AnitaGhanotakis, Demetrios DemetriosScocchi, MarcoGharibyan, Anna L.Scognamiglio, MonicaGhatak, ArindamScott, Amy M.Ghiasvand, AlirezaScott, PeterGhica, Mariana EmiliaScotti, MarcusGhilardi Lago, João HenriqueScotti, NicolaGhosal, SutapaScrima, MarioGhosh, AbhijitSczepanski, JonathanGhosh, MonojSebesta, RadovanGhosh, SandeepSebestik, JaroslavGiacomazza, DanielaSeca, Ana M. LGiacometti, JasminkaSeca, Ana Maria Loureiro DaGiacomino, AgneseSedic, MirelaGiamperi, LauraSeelam, Prem KumarGiampietro, LetiziaSeene, TeetGiannakas, ArisSegale, LorenaGiannakopoulos, PanteleimonSegundo, MarcelaGiannini, CleliaSehgal, InderGiansanti, LuisaSeidl, ClaudiaGiarratana, FilippoSeidl, P. R.Gibasiewicz, KrzysztofSeifitokaldani, AliGiebultowicz, JoannaSeijas Vázquez, Julio A.Gieleciak, RafalSeijo, M. CarmenGierlinger, NotburgaSeipel, KatjaGigli, MatteoSekara, AgnieszkaGigliotti, Casimiro LucaSekiya, MizukiGikas, EvagelosSekutor, MarinaGil, M.L.A.Selan, LauraGil, MinchanSeley-Radtke, KatherineGilch, PeterSelig, MalteGilis, DimitriSellars, JonathanGilje, John W.Selleri, SilviaGillan, Edward G.Selopal, Gurpreet SinghGillanders, Ross NSemeniuc, Cristina AnamariaGillespie, James W.Semenyuk, PavelGilmer, JohnSemsarilar, MonaGil-Muñoz, RocioSena, Marcelo MGil-Rubio, JuanSenbonmatsu, TakaakiGimeno, ConcepciónSenderowitz, HanochGiner-Casares, Juan J.Sendón, RaquelGiokas, DimosSendra, EstherGiorgi, GiorgioSene, NdolaneGiorgio, SelmaSenge, MathiasGiorno, LidiettaSengupta, ArjunGiovagnoli, StefanoSengupta, BidishaGiovanni, RotiŞenilă, MarinGiovannoni, RobertoSenkovska, IrenaGiovinazzo, GiovannaSenz Diez, María TeresaGiró Paloma, JessicaSeo, YongbeomGismondi, AngeloSepuru, Krishna MohanGiubileo, FilippoSequeda-Castañeda, Luis G.Giudetti, AnnaSerafín, VerónicaGiuffrè, Angelo MariaSerdaroğlu, GoncagülGiuffrida, AlessandroSerdyuk, Olga V.Giungato, PasqualeSerefko, AnnaGiurg, MirosławSereikaite, JolantaGłąbska, DominikaSerikov, ArkadyGlebe, UlrichSerio, AnnalisaGlebov, Evgeni M.Serpil, DemirciGlobisch, DanielSerra, StefanoGloess, Alexia N.Serralheiro, Maria LuísaGlover, Stephen A.Serrano, MónicaGłuszyńska, AgataSerrano-Aroca, ÁngelGmurek, MartaSertić, MirandaGnat, SebastianSetaka, WataruGniewosz, MałgorzataSethi, GautamGnyba, MarcinSetny, PiotrGobeil Jr, FernandSeto, ShigekiGóbi, SándorSetyaningsih, WidiastutiGobis, KatarzynaSetzer, WilliamGodlewska-Żyłkiewicz, BeataSetzer, William N.Goel, Peeyush N.Sevic, DragutinGoel, SarikaSevilla-Morán, BeatrizGoettert, MarciaSeymour, MathewGoga, MichalSgarbanti, MarcoGogulamudi, Venkateswara ReddySgherri, CristinaGohy, Jean-FrancoisSgrignani, JacopoGojo, SatoshiShaaban, Khaled A.Golanov, Eugene V.Shabalin, IvanGolding, JonShah, RohanGoldoni, LucaShai, YechielGolic-Grdadolnik, SimonaShakeri, MajidGolinelli, Marie-PierreShamay, YosefGolkowski, CzeslawShamonin, MikhailGoltz, Douglas M.Shan, XinGolub, EyalShang, ChaoqunGolub, IgorShankar, EswarGolubkina, NadezhdaShao, FangweiGombart, AdrianShao, HuaGomes da Cruz, AdrianoShao, HuaiyuGomes, Ana P.Shao, LeiGomes, Clara S. B.Shao, YihanGomes, Maria SaloméShariare, MohammadGomes, NelsonSharma, AbhisheakGomes, SóniaSharma, AnirudhGómez Barrios, XiomarSharma, AshutoshGómez Castellà, CristinaSharma, AtulGómez De Cedrón, MartaSharma, DeepikaGómez Pinchetti, Juan LuisSharma, KavitaGomez, DanielSharma, Piyush SindhuGomez-Crisostomo, RocioSharma, PriyankaGómez-Graña, SergioSharma, RitinGómez-Plaza, EncarnaSharma, Shiv K.Gomez-Ruiz, SantiagoSharma, Sunil K.Gomha, SobhiShaughnessy, KevinGomis-Rüth, F. XavierShaw, SubrataGonçalves De Araújo, ThaiseShe, Zhi-GangGonda, SandorShehu, AmardaGonda, SándorSheldon, RogerGondi, Christopher S.Sheldrake, HelenGonec, TomasShellaiah, MuthaiahGoněc, TomášShellie, RobertGong, HuaShen, KelvinGong, XingchuShen, Szu-ChuanGonsalvi, LucaShen, Tang-LongGonzález Cappa, Stella MarisShen, XiaoGonzález Domínguez, RaúlShen, XingguiGonzález Laredo, Rubén F.Shen, XiulongGonzález, Concepción C.Shen, YuemaoGonzález, Florenci V.Sheng, RongGonzalez, Juan M.Sheng, RuilongGonzález, Luis CrovettoSheri, MadhuGonzález, MartaSherrell, PeterGonzález, SoniaSherwood, JamesGonzalez, VictorSheu, Joen-RongGonzalez-Aguilar, GustavoShevtsova, Elena F.González-Domínguez, RaúlShewmaker, Frank P.González-García, JorgeShi, HuaGonzález-Laredo, Rubén FranciscoShi, Jie-huaGonzález-Maya, LeticiaShi, Yan-HongGonzález-Minero, Francisco JoséShi, YongqianGonzález-Miret, M. LourdesShi, ZengqianGonzález-Pérez, JoséShiao, Young-JiGonzález-Vallinas, MargaritaShiau, L. D.González-Vera, Juan A.Shibata, IkuyaGonzález-Zamora, EduardoShibata, ToshiyukiGoosen, Neill J.Shibatomi, KazutakaGoppelt-Struebe, MargareteShibayama, NobukoGordillo Arrobas, BelénShidoji, YoshihiroGórecki, MarcinShigdar, SarahGori, AntonellaShigemori, HideyukiGornas, PawelShigemura, YasutakaGornowicz, AgnieszkaShih, Shao-juGoroncy, AlexanderShikov, AlexanderGorrasi, Ilaria Silvia RossellaShim, HyunboGórska, SabinaShimada, YasuhitoGorska-Ponikowska, MagdalenaShimanouchi, ToshinoriGorski, ŁukaszShimizu, KazuyukiGórski, ŁukaszShimizu, TakahikoGościańska, JoannaShimpi, ManishkumarGossage, R. A.Shimura, TatsuoGoswami, SubhadipShin, Beom SooGoszczyński, TomaszShin, Crystal S.Goto, MasuoShin, Eun JuGoto, YosukeShin, Hyun-JaeGotsbacher, MichaelShin, Jae SupGouault, NicolasShin, JongheonGoudarzi, MaryamShin, MalshickGoulas, VlasiosShin, SoyoungGouvinhas, IreneShin, Young G.Gouws, ChrisnaShinada, TetsuroGoverna, PaoloShinde, SudhirkumarGowda, KrishneShinohara, MitsuruGoya, LuisShinomiya, KazufusaGozin, MichaelShioda, NorifumiGrabarczyk, MałgorzataShiono, TakeshiGrabauskas, GintautasShiragami, TsutomuGraber, Zachary T.Shirahata, TatsuyaGrabowski, SlawomirShirai-Matsumoto, KeikoGrabska, JustynaShirakami, YoheiGraczyk-Jarzynka, AgnieszkaShirato, KenGradoni, LuigiShoeva, OlesyaGraebin, CedricShoffstall, Andrew J.Graikou, KonstantiaShoji, MitsuoGrajek, HenrykShokal, UpasanaGramlich, MichaelShokouhimehr, MohammadrezaGramza – Michałowska, AnnaShornick, LaurieGramza-Michałowska, AnnaShosha, EsraaGranato, DanielShozeb, HaiderGrancha, ThaisShrestha, Vivek RajGrande Tovar, Carlos DavidShrivastava, AbhishekGrande, RossellaShu, QinghaiGrandi, NicoleShu, XinhuaGrandinetti, FeliceShui, Hao-AiGranica, SebastianShults, Elvira E.Grant, David C.Shumoy, HabtuGrant, GeorgeShymanovich, TatsianaGrasselli, ElenaSiano, FrancescoGrassi, MarioSiatka, TomášGrassia, PaulŠic Žlabur, JanaGravalidis, ChristoforosSiciliani, MarioGravel, EdmondSiciliano, CarloGraves, Steven WSiczek, MiłoszGray, ChristophertSiddharth, SumitGrazulevicius, JuozasSiddique, Abu BakarGreatrex, BenSidorenko, Viktoriya S.Greco, AntonioSidorova, YuliaGreen, BenSiebenhofer, MatthäusGreen, BrianSieber, FritzGreen, IvanSiebert, Hans-ChristianGreenhalgh, DavidSiegel, JakubGreenwood, Michael T.Sieniawska, ElwiraGreetham, DarrenSierecki, EmmaGregáň, JurajSieroslawska, AnnaGregor, IngoSierra-Campos, ErickGregori, AlbertoSierra-Rosales, PaulinaGregory, Stephen L.Siesler, Heinz WilhelmGrembecka, MalgorzataSiessere, SelmaGrider, ArthurSignore, GiovanniGrieco, DomenicoSignorile, MatteoGriesbeck, Axel G.Sikazwe, DonaldGrilc, MihaSikder, PrabahaGris, DenisSikirzhytski, VitaliGrishin, MaximSikora, MariuszGrisoni, FrancescaSilaghi-Dumitrescu, RaduGrkovic, TanjaSilakhori, MahyarGrob, KoniSild, SulevGröger, ThomasSilenko, Alexander J.Groh, Isabel Anna MariaŠiler, BranislavGrohmann, UrsulaSiligardi, GiulianoGrolli, StefanoSilina, Yuliya E.Gross, AaronSilipo, AlbaGross, Joshua B.Silk, PeterGrosse, StephanSilkina, AllaGrossi, GiancarloSilva, Amelia MGrossi, MarcoSilva, Ana M.g.Grosso, GiuseppeSilva, ArturGroves, PatrickSilva, CarlaGruber, ChristianSilva, Carlos AlbertoGrudnik, PrzemyslawSilva, FilomenaGrul’ová, DanielaSilva, João PedroGrulke, ChristopherSilva, JoséGrumezescu, Alexandru MihaiSilva, Maria FátimaGryczynski, IgnacySilva, PaulaGryko, DorotaSilva, Pedro JorgeGrynkiewicz, GrzegorzSilva, RicardoGrzegorczyk, IzabelaSilva, Saimon MoraesGu, BaohuaSilva, TaniaGu, HongzhouSilva-Martínez, SusanaGu, LipingSilve, AudeGu, ZhenSilveira, Nádya P. DaGualandi, AndreaSilvestre, SamuelGuarrotxena Arlunduaga, Miren NekaneSilvestris, NicolaGuascito, Maria RacheleSim, TaehoonGuatame-Garcia, AdrianaSimal, JesusGubanska, IgaSimari, CataldoGüder, FiratSimeonov, VasilGudmundsdottir, AnnaSimes, DinaGuedes, Rita C.Simiczyjew, AleksandraGuedes, Rui MirandaSimirgiotis, MarioGuenet, Jean-MichelSimirgiotis, Mario J.Guerlesquin, FrancoiseSimko, FedorGuerriero, GiuliaSimm, RogerGuerrini, MarcoSimmons, KatieGuescini, MicheleSimões, PedroGuha, SoniaSimões, Zilá Luz PaulinoGuiamet, Patricia S. Simon Brigida, Fernandez DeGuido, LuisSimon, JulianGuidotti, MatteoSimón, LuisGuillaume, PierreSimon, PeterGuillemin, Jean-ClaudeSimončič, BarbaraGuillén-Bejarano, RafaelSimone, Angela DeGuimarães, Allan J.Simone, ElenaGuiné, RaquelSimone, VincenziGuix, Francesc XavierSimonet Morales, Ana MariaGulic, TamaraSimonetti, GiovannaGulicovski, JelenaSimoni, FrancescoGun’ko, Vladimir M.Simos, YannisGunaje, JayaramaSinanoglou, VassiliaGunasekara, SamanSinditskii, V. P.Gunawidjaja, RaySingh, AnilGuncheva, MayaSingh, ArunimaGünes, CagataySingh, ChanpreetGunia-Krzyżak, AgnieszkaSingh, Deepak PratapGunji, TakaoSingh, JitendraGuo, Chih-HungSingh, JonathanGuo, FenghaiSingh, KamalendraGuo, JingshuSingh, KamayaniGuo, LiangqiaSingh, RaghurajGuo, Ming-quanSingh, SarbjitGuo, SiweiSingh, Surya P.Guo, TingSingha, SubhankarGuo, WeiminSingla, BhupeshGuo, XiaoyuSinha, AbhijeetGuo, XinboSinha, KalyanGuo, Zhan-HuSinha, Sudarson SekharGuo, ZhengSiniscalco, DarioGupta, CharuŠinko, GoranGupta, KajalSinnott, Michael L.Gupta, Ram K.Sintim, HermanGupta, SanjaySiodłak, DawidGus’kov, VladimirSiopa, FilipaGusev, AlexanderSipos, LászlóGushchin, Artem L.Sippl, WolfgangGushina, IrinaSiracusa, RosalbaGuskov, AlbertSiracusa, ValentinaGuskova, OlgaSiren, HeliGust, RonaldSirsat, SujataGustafson, KirkSiskos, Michael G.Gutierrez Ramirez, ManuelSissi, ClaudiaGutiérrez, GemmaSistla, SrinivasGutiérrez, MargaritaSitarek, PrzemysławGutiérrez-Capitán, ManuelSithole, BruceGutiérrez-Gallego, RicardoSivaev, Igor B.Gutiérrez-Juárez, RogerSivakumar, SushamaGutiérrez-Uribe, JanetSivanesan, IyyakkannuGutowska, IzabelaSivasankaran, HarishGütschow, MichaelSivathanu, VivekGuzmán Ruiz, RocíoSiwek, AgataGuzman, FannySkalniak, LukaszGuzow-Krzemińska, BeataSkarková, VeronikaGyémánt, GyöngyiSkarżewski, JacekH. Gonzalez, DanielSkellam, ElizabethH. Moreno, Paulo RobertoSkenderidis, ProdromosHa, EunyoungSkibiński, RobertHa, HoehunSkidmore, MarkHaak, AndrewSkoko, ŽeljkoHabala, LadislavSkorik, Yury A.Habaue, ShigekiSkorko-Glonek, JoannaHaberl, BiancaSkotak, MaciejHabuda-Stanic, MirnaSkouta, RachidHada, MasahikoSkrabana, RostislavHadden, KyleSkroza, DanijelaHadjikakou, Sotiris K.Skrzydlewska, ElzbietaHadjipavlou-Litina, DimitraŠkulcová, AndreaHa-Duong, TâpSkwarek, EwaHagar, MohamedSlama, James T.Hagelueken, GregorSlavik, RogerHagerman, AnnSlavov, Svetoslav NanevHägglund, Per MårtenŚliwińska-Wilczewska, SylwiaHaidar, SamerSliwinski, TomaszHaiges, RalfŚliwka-Kaszyńska, MagdalenaHajabdollahi, FarzanehSlobodnikova, LiviaHalagarda, MichałSłoczyńska, KarolinaHalder, Amit KumarSłomińska-Wojewódzka, MonikaHalder, AvikSłomkiewicz, Piotr MarekHalet, Jean-FrançoisSlouka, ChristophHall, F. ScottSlowinska, KatarzynaHam, Young WanŚlusarczyk, SylwesterHamaguchi, MasahideSmani, YounesHamberger, BjörnŠmejkal, KarelHamon, Jean-RenéSmetana, MichaelHampel, DanielaSmetana, VolodymyrHan, AidongSmiesko, MartinHan, Byung WooŚmigielski, Krzysztof B.Han, JaehongSmith Callahan, Laura A.Han, JianlinSmith, Aaron T.Han, Jong WonSmith, JenniferHan, Quan-BinSmith, Judith A.Han, YuyanSmith, KerryHanai, ToshihikoSmith, MarkHanaoka, HirofumiSmodiš Škerl, MajaHanaor, DorianSmolensky, DmitriyHancock, SarahSmolikova, GalinaHancu, GabrielSmonou, IouliaHanda, MakotoSmulders, MaartenHandy, ScotSmułek, WojciechHandzlik, JadwigaSnapper, MarcHanif, MuhammadSnegur, LubovHankir, Mohammed K.Snell, Terry W.Hanne, HoffmannSnežana, PapovićHano, ChristopheŚNIEGOCKI, TOMASZHanoune, BenjaminSnow, NicholasHansen, NielsSnow, Nicholas H.Hanski, LeenaSnowden, TimothyHao, YongSo, Hong-SeobHarasym, JoannaSoares, Ana RaquelHarbottle, DavidSobarzo-Sánchez, EduardoHardwick, James P.Sobeh, MansourHardy, Jeanne A.Sobiesiak, MagdalenaHardy, John GeorgeSobisch, TitusHargreaves, JustinSobotta, LukaszHarijan, Rajesh KSobotta, ŁukaszHarijith, AnanthaSocaci, SoniaHarikrishna, KommidiSocas-Rodríguez, BárbaraHarings, J.A.W.Socol, MarcelaHarmanci, Arif OzgunSoengas, Raquel G.Harp, Joel M.Soeta, TakahiroHarper, JamesSogorb Sánchez, Miguel ÁngelHarrell, William R.Sohal, SandeepHarrington, CharlesSohng, Jae KyungHarris, Audray K.Sokół-Łętowska, AnnaHarris, DanniSokolov, MaximHarris, TrevorSokolová, RomanaHarrison, Paul H. M.Solache-Ríos, Marcos JoséHarrison, Roger G.Šolaja, Bogdan A.Hart, Sarah R.Solano, FranciscoHartman, MariuszSolans-Monfort, XavierHartman, Matthew C.T.Soler, Miguel ÁngelHartung, JensSoler-Vasco, LauraHaruki, MitsuruSoleti, RaffaellaHasan, MehediSolhaug, Knut AsbjørnHasanzadeh Kafshgari, MortezaSoliven, ArianneHasegawa, MorifumiSolomon, MelaniHasegawa, UraraSolovchenko, AlexeiHashimoto, Makoto (Hokkaido University)Solovyev, NikolayHashimoto, Makoto (Tokyo Metropolitan Institute of Medical Science)Somani, SukrutHashimoto, MasanoriSomlyai, GáborHashimoto, MasayukiSommer, MichaelHaslinger, KristinaSon, Seung UkHassan, Amr AbdulfattahSon, Young-gyuHassan, Amro B.Sonda, SabrinaHassan, ErrolSong, ChunhuaHassan, Sherif T. S.Song, HailongHassanali, Ali A.Song, Im SookHastings, GarySong, JaewhanHatano, AkihikoSong, JeongminHatano, TsutomuSong, JianweiHati, SantanuSong, JinshengHattori, TomokazuSong, JoeHatzakis, EmmanuelSong, Jong-WonHaubrich, Brad A.Song, Kwang HoHaudecoeur, RomainSong, MinjungHausmann, MichaelSong, Yang-HeonHausner, GeorgSonntag, Matthew D.Havlik, JaroslavSontakke, Atul D.Hawkins, BillSophocleous, MariosHawrył, Anna M.Sorcar, SauravHawrył, MirosławSørensen, Jens LauridsHayakawa, KazuichiSorensen, JohnHayashi, NorikoSørensen, Klavs MartinHayashi, YoshihitoSorokina, I. V.Hayes, Sidney J.Sorrenti, ValeriaHayhurst, AndrewSorrentino, AndreaHaynes, CallySorrentino, E.Haynes, RichardSorsa, TimoHazai, LászlóSosic, AliceHazell, GavinSosic, IzidorHe, ChengweiSosnowska, AnitaHe, HailunSosnowska, DorotaHe, HaiyingSosnowski, Tomasz R.He, LiliSotelo Mundo, RogerioHe, PeigangSotiriadis, KonstantinosHe, PingliSoto-Hernández, MarcosHe, YongSoto-Reyes, ErnestoHeber, DieterSoto-Reyes, IleanaHeeley, Ellen L.Soulère, LaurentHegde, Muralidhar L.Soundararajan, MeeraHegde, VijaySour, AngéliqueHegedűsová, AlžbetaSoural, MiroslavHeger, DominikSousa, AnaHeider, NorbertSousa, ClaraHeine, ElisabethSousa, Joana L. C.Heinzmann, Silke SophieSousa, Sérgio F.Heise, Herbert MichaelSousa, Sérgio FilipeHeiskanen, JuhaSouza-Garcia, JanainaHeld, ChristophSovova, HelenaHelliwell, JohnSowa, IreneuszHelms, VolkhardSpagnuolo, PaulHemming, KarlSpahn, ViolaHenderson, WilliamSpalletti, AnnaHenen, Morkos A.Spanakis, MariosHenkel, JaninSpano, GiuseppeHenriques, Marta H. F.Spanopoulos, IoannisHepnarova, VendulaSpasiano, DaniloHerbert, JohnSpasojevic, Pavle M.Herczegh, PálSpear, MorwennaHerfindal, LarsSpégel, PeterHermann, PetrSpelzini, DaríoHernández, IgnacioSpendier, KathrinHernandez, Jean-FrançoisSpengler, Dietmar H.Hernandez-Hernandez, OswaldoSpengler, GabriellaHernández-León, AlejandroSpiegler, VerenaHernández-Mesa, MaykelSpivak, DavidHernandez-Montelongo, JacoboSpizzirri, U GianfrancoHernández-Núñez, EmanuelSpoerri, LoredanaHerndon, James W.Spooner, NeilHerrera, RaquelSpringer, ValeriaHerrero-Martínez, José ManuelSpur, Bernd W.Herrmann, ChristianeSpychaj, TadeuszHess, GrzegorzSpyridon N., KarrasHess, MichaelSquillaro, TizianaHetényi, CsabaSreedharan, SreejeshHettegger, HubertSridhar, JayalakshmiHevey, RachelSridhar, RadhakrishnanHibino, TakashiSrinivas, KeerthiHigashi, KyoheiSrinivasan, BharathHikawa, HidemasaSrinivasan, SeshaHildner, RichardSriram, GopuHildreth, SherrySrivastava, Sanjay K.Hilhorst, MarcSrivedavyasasri, RadhakrishnanHill, GrantSroka, ZbigniewHilloulin, BenoîtStacchiotti, AlessandraHills, RonaldStachelska-Wierzchowska, AlicjaHimoto, TakashiStadnik, JoannaHinchliffe, PhilipStagos, DimitriosHindocha, ChandniStaicu, TeodoraHines, Justin K.Stalikas, ConstantineHinze, ThomasStana, AncaHirabayashi, JunStanculescu, IoanaHirakawa, HidehikoStankevic, MarekHirano, MasafumiStanković, DaliborHirashita, TsunehisaStanstrup, JanHirata, YokoStarek, MałgorzataHirayama, FumitoshiŠtarha, PavelHirose, TakashiStarowicz, MałgorzataHirota, YuichiroStarynowicz, PrzemyslawHiroyuki, MatsuiStarzak, KarolinaHisada, KenjiStasiak-Różańska, LidiaHitachi, KeisukeStaszak, KatarzynaHlina, Johann A.Stavrianidi, AndreyHlozek, TomasSteed, JonathanHnyk, DrahomírStefane, BogdanHo, JunmingStefano, Stefano DiHo, Megan Yi-PingStefanon, BrunoHo, MengfeiStefanowicz-Hajduk, JustynaHo, Shih-ChingStefańska, PatrycjaHo, Su-ChenStein, ViktorHo, Wing ShingSteinbach, DavidHo, Yi-ChengSteindel, MárioHobbs, Christopher E.Steiner, DeniseHocek, MichalStellato, FrancescoHodaifa, GassanStelzer, FranzHodges, H. CourtneyStepanić, VišnjaHodgson, David R. W.Stephan, HolgerHoehn, KyleStephen, Michael RajeshHofbauer, StefanStephens, JonathanHoffman, AngelaStępień, ŁukaszHoffmann, Lucia VieiraŠtěpnička, PetrHofmann, AndreasStepnowski, PiotrHofmann, UteSteup, MartinHögger, PetraStewart, GrahamHohensinner, PhilippStieger, BrunoHohmann, JuditStilinović, VladimirHoldsworth, CloviaStiriba, Salah-EddineHolguin, Francisco OmarStobiecki, MaciejHolt, Stephen A.Stocchero, MatteoHolynska, MalgorzataStockner, ThomasHolzgrabe, UlrikeStoikov, Ivan IvanovichHomma, Miwako KatoStojakowska, AnnaHonciuc, AndreiStojan, JureHonda, ChikakoStojko, JerzyHonda, MasakiStojković, DejanHong, KuiStojmenović, MarijaHong, Mee YoungStoleru, ElenaHong, Seok HoonStoll, RaphaelHong, Seung-HeonStoppato, AnnaHong, TaoStornaiuolo, MarianoHong, Yeol YoonStote, KimHong, YuningStoychev, GeorgiHonório, KáthiaStrąkowska, AnnaHöpfl, HerbertStrankowski, MichalHorak, EmaStrati, Irini F.Horax, RonnyStreb, CarstenHorel, ÁgotaStrehlitz, BeateHorhat, Florin GeorgeStrekowski, LucjanHori, AkikoStrekowski, RafalHorino, YoshikazuStringaro, AnnaritaHoriuchi, YasueStripp, Sven T.Horne, William SethStrube, JochenHörner, GeraldStruga, MartaHorobin, Richard WŠtrukil, VjekoslavHorrocks, RichardStruzik, JustynaHorsmans, YvesStrzałkowski, KarolHorvai, GeorgeStrzemiecka, BeataHorvath, DragosStucchi, MartaHorváth, OttóStudt, LenaHoshino, NorihisaSTUDZIŃSKA-SROKA, ELŻBIETAHosohata, KeikoStudzinski, George P.Hosokawa, SeijiroStührwohldt, NilsHosomi, RyotaStuper-Szablewska, KingaHossain, M.NurSturesson, ChristianHostaš, JiříSturlese, MattiaHosur, VishnuSturtevant, JoyHou, HarveyStürup, StefanHoung, Jer-YiingStyczyński, JanHousecroft, CatherineSu, An-ChungHouzé, PascalSu, DanHowarth, AshleeSu, Hai-ChingHowell, BobSu, HongmeiHowes, Laurence GuySu, JunfengHoyos, PilarSu, PeifengHrabal, RichardSu, Wen-TaHrckova, GabrielaSu, Zheng-YuanHrobárik, PeterSuárez-Roca, HebertoHroboňová, KatarínaSubasi, AbdulhamitHryciw, Deanne H.Subbiah, MuruganHsia, Shih-MinSubirana, JuanHsieh, Chang WeiSubramanian, ChitraHsieh, Chang-WeiSuda, YoshihitoHsieh, ShulingSudac, DavorinHsin, Kun-YiSudar, MartinaHsu, Cheng-KuangSuddala, Krishna ChaitanyaHsu, Chih-ChiehSudo, AtsushiHsu, Day-ShinSugahara, TakuyaHsu, Fei-TingSugai, TakeshiHsu, Jun-TeSugamata, KohHsu, Shang-Te DannySugase, KenjiHsu, Sodio C.N.Sugawara, AkihiroHsuan, JustinSugawara, TatsuyaHsueh, Yi-HuangSuh, Dae-yeonHU, CHE-CHIASuh, Won HyukHu, ChenlinSuh, YongyoonHu, ChunSuhail, YasirHu, FangzhongSuk, Ki TaeHu, GuangSukhikh, TaisiyaHu, GuokuSukumar, DeepthaHu, GuoliSuleria, Hafiz Ansar RasulHu, JunhuiSullivan, ConHu, MinSulová, ZdenkaHu, Shang-HsiuSülsen, ValeriaHu, Wei-PingSun, ChengwenHu, WenhuiSun, DiHu, Wen-YangSun, GongqinHu, XiaosuSun, GuohuiHu, YaxiSun, GuoxingHuang, Chao-MingSun, HaoHuang, Chih-ChiaSun, JianghaoHuang, Chih-FengSun, Jing ZhiHuang, Chiung-ChengSun, JingjingHuang, Chun-YungSun, LinaHuang, Guan-JhongSun, MeijunHuang, Huey-ChunSun, WujinHuang, Hui-ChiSun, XiaofeiHuang, Jason C.Sun, YiHuang, JingfengSunatsuki, YukinariHuang, LimingSundar, Victor JohnHuang, LinfangSundén, HenrikHuang, Nai-KueiSung, Ping-JyunHuang, RimingSung, Wen-ChiehHuang, Shu-PinSuntharalingam, KogularamananHuang, WeixinSuperti, FabianaHUANG, Wen-ChinŠupová, MonikaHuang, Wen-ChungSurber, ChristianHuang, Wu-JangSurguchov, AndreiHuang, XiaodanSurowska, BarbaraHuang, XiaoyuSusic, Michaelhuang, xueshiSut, StefaniaHuang, Ya-lingSutherland, Hamish S.Huang, YuanyuSutradhar, ManasHuang, YunlongSüzen, SibelHuang, YuxiongSuzui, MasumiHuang, ZiweiSuzuki, KatsuhikoHuber, ImreSuzuki, MasaakiHübner, ChristianSuzuki, ToshikazuHudalla, Gregory A.Suzuki, YukiHuefner, AntjeSvavarsson, Halldor GudfinnurHuerta-Angeles, GloriaSvete, JurijHuertas, Jesús R.Svetlana, Tretsiakova-McNallyHuh, Yun SukSvetlov, MaksimHui, Patrick Chi-leungSvidzinski, Terezinha Inez EstivaletHuisman, MarcSvingen, TerjeHulme, ChristopherSvoronos, Paris D.N.Humphries, BrockŚwiątek, ŁukaszHung, AndrewŚwiątek, PiotrHunt, Piper ReidŚwiderek, KatarzynaHunter, LukeSwieca, MichałHuntosova, VeronikaSwiecilo, AgataHuot, JacquesŚwietlik, RomanHuppi, KonradSwiezewska, EwaHur, YoonkangSwinarew, AndrzejHura, TomaszSykłowska-Baranek, KatarzynaHurtado Oliva, Miguel ÁngelSykora, DavidHussain, HidayatSynytsya, AndriyHussein, AhmedSyrpas, MichailHussein, WaleedSytar, OksanaHusson, JeromeSytykiewicz, HubertHutter, MichaelSzabelski, PawełHutter, TanyaSzabla, RafałHwang, Bang YeonSzacon, ElzbietaHwang, Chi-SunSzafranski, KrzysztofHwang, Dae YounSZAJDAK, Lech WojcienchHwang, Gil TaeSzakiel, AnnaHwang, Long-ChihSzakonyi, ZsoltHwang, SungyeonSzala, MateuszHymel, DavidSzaleniec, MaciejHyun, Tae KyungSzczepański, PiotrIaccino, EnricoSzczes, AlexandraIacovino, RosaSzczolko, WojciechIadarola, PaoloSzczurek, AndrzejIannazzo, DanielaSzebenyi, MarianIatsunskyi, Igor RSzederkenyi, GaborIbarra, Israel S.Szegedi, ViktorIbrahim, FandiSzeiffova Bacova, BarbaraIbrahim, Mohamed AliSzeleszczuk, ŁukaszIbrahim, SalamSzeliga, MonikaIbrahim, Sherif AbdelazizSzeluga, UrszulaIchikawa, SatoshiSzewczuk, Myron R.Iciek, MałgorzataSzewczyk, AgnieszkaIclodean, CalinSzewczyk, KatarzynaIdo, YasuoSzikra, DezsőIebba, ValerioSzilagyi, AndrasIelciu, IrinaSzilágyi, LászlóIentile, RiccardoSzöllősi, DánielIfelebuegu, AugustineSzolnoki, BeátaIftekhar, SidraSzőri, MilánIglesias, EmiliaSzostak, MichalIglesias, MartaSzostak, RomanIglesias, MartínSztanke, MałgorzataIgne, BenoîtSzukalski, AdamIgnjatovic, NenadSzulc-Dąbrowska, LidiaIkai, TomoyukiSzulczyński, BartoszIkegami, TakahisaSzultka-Młyńska, MałgorzataIkeguchi, MasamichiSzumny, AntoniIkehara, KenjiSzweda, PiotrIkehata, AkifumiSzweda, PoitrIkura, TeikichiSzwengiel, ArturIlagan, Ma. Xenia G.Szymanowska, UrszulaIlahi, BouraouiSzymańska, GrażynaIlia, GheorgeSzymanska-Chargot, M.Ilia, GheorgheSzyszkowicz, MieczysławIlic, NeboksaT. Carvalho, DiogoIliev, BoyanTa, HangIlla, OnaTabacchi, GloriaIllescas, JavierTabarrini, OrianaIlluminati, SilviaTabudravu, Jioji N.Imai, ToshioTadić, VanjaImai, YoichiTaghibiglou, ChangizImamoto, YasushiTagit, OyaImamura, MasahiroTagliatesta, PietroImhof, PetraTaglietti, AngeloImlach, Wendy L.Taguchi, Y-h.Imm, Jee-YoungTaha, MuhammadImperatore, ConcettaTaheri, ShimaInada, NatáliaTai, William Chi-ShingInada, RyojiTaira, JunseiInagaki, HidetoshiTakac, TomasInguimbert, NicolasTakada, AkihikoIno, KosukeTakafuji, MakotoInokuchi, TsutomuTakagi, NoriakiInokuma, TsubasaTakahashi, FumikiInoue, AtsukoTakahashi, HideyukiInoue, KazushiTakahashi, KaitoInoue, SeijiTakahashi, KoretaroInoue, ShigeyoshiTakahashi, KosakuInukai, JunjiTakahashi, OhgiInuzuka, ToshiyasuTakahashi, TakuInyushin, Mikhail Y.Takale, BalaramIoannides, TheophilosTakanami, ToshikatsuIoelovich, Michael YaTakano, YuIonete, Roxana ElenaTakasu, KiyoseiIonita, GabrielaTakaya, TomohisaIonut, Ioana A.Takayanagi, ToshiyukiIordache, FlorinTakayoshi, SuzukiIorio, EgidioTakeda, YouheiIorizzi, MariaTakemori, HiroshiIpsakis, DimitrisTakemoto, HiroyasuIqbal, Hafiz M. N.Takenaka, ShigeoriIrakli, MariaTakeoka, ShinjiIrez, Alaeddin BurakTakeuchi, OsamuIrie, TetsumiTakizawa, ShinobuIriepa Canalda, IsabelTakizawa, ShinyaIrina Vlasova-St., LouisTalele, Tanaji T.Irina, CarlescuTalhi, OualidIron, MarkTalib, WamidhIsaza Martínez, José HipólitoTalukder, PoulamiIsemura, MamoruTaly, AntoineIshiguro, HitoshiTam, Eric Kwok WaiIshii, KenichiroTamaian, RaduIshii, TakahiroTamamura, HirokazuIshizuka, TakumiTamayo-Ortiz, MarcelaIsland, J. O.Tampieri, FrancescoIslas-Jácome, AlejandroTampucci, SilviaIsmael, M. IsabelTamura, OsamuIsmaili, LhassaneTamura, RuiIsman, Murray B.Tan, ChenIsopescu, RalucaTan, JianengItahana, KojiTan, Kui-ThongItahashi, SyuichiTan, LongzhiIto, HiroyasuTan, Swee ChingIto, MichihoTan, YanglanIto, MikioTan, YingIto, TakamichiTan, ZhijianIto, TakeruTanabe, KatsuyukiIto, TakuyaTanabe, KenjiIto, YujiTanabe, YooItoh, YukihiroTanaka, HidenoriItou, JunjiTanaka, KeijiItsuno, ShinichiTanaka, RyoIturriaga-Vasquez, PatricioTanaka, ShigenoriIvanda, MileTanaka, TakayukiIvanov, AlexanderTanaka, TakujiIvanov, AndreyTanaka, TomonariIvanov, Ilia N.Tananaiko, OksanaIvanov, SergeyTanase, CorneliuIvanov, Yuri D.Tančinová, DanaIvanova, Alla V.Tanemura, KiyoshiIvanova, BojidarkaTang, AihuaIvanova, Olga A.Tang, Chih-HsinIvashchenko, OlenaTang, JingIvshina, IrenaTang, LongtengIwai, AtsushiTang, WeiweiIwamoto, YujiTang, YuanIwaniak, AnnaTanigawa, TetsuyaIwasaki, TakashiTaniguchi, HiroakiIzawa, HironoriTaniguchi, NobukazuIzumi, YoshihiroTaniguchi, YasushiIzumi, YudaiTanimoto, HirokiJ Edagwa, BensonTankiewicz, MaciejJ. Barba, FranciscoTankiewicz-Kwedlo, AnnaJablan, JasnaTanner, EdenJabłońska-Trypuć, AgataTanner, Julian A.Jacek Koziel, JacekTanner, Julian AlexanderJackson, RyanTannous, KatiaJackson, William F.Tantos, ÁgnesJacob, FrancisTao, FeifeiJacob, GopasTao, LimingJacob, IsaacTao, ShanwenJacomin, Anne-ClaireTao, W. AndyJadeja, RavirajsinhTao, Ya-XiongJadwicienczak, WojciechTao, YunwenJaenicke, StephanTapia, Alejandro A.Jaffrezic-Renault, NicoleTarabanko, Valery E.Jagerovic, NadineTaranto, Alex GutterresJagoda, ElaineTarkowská, DanušeJahandideh, SamadTarozzi, AndreaJahncke, MichaelTarr, Matthew A.Jain, VaibhavTartaglione, LucianaJairo H., López-VargasTartour, EricJaiswal, Jagdish KumarTashima, ToshihikoJakasa, IvoneTasic, LjubicaJaki, BirgitTatulian, Suren A.Jakobek, LidijaTaub, MaryJakobusic Brala, CvijetaTauchen, JanJakopic, JernejaTava, AldoJakubczyk, AnnaTavanti, FrancescoJakubowski, WitoldTavares, José CarlosJamieson, AndrewTavares, Josean FechineJampilek, JosefTaviano, Maria FernandaJamwal, RohitashTavío, María Del MarJanczarek, MarcinTawfik, Sherif AbdulkaderJańczuk, BronisławTaylor, MatthewJanda, PavelTaylor, RebeccaJanecki, TomaszTaylor, SpencerJaneczko, MonikaTecilla, PaoloJaneczko, TomaszTedesco, IdoloJänes, AlarTeeri, TeemuJaneta, MateuszTeixeira, FilipeJang, Dae-sikTeixeira, PaulaJang, Hae WonTeixeira, RitaJangampalli Adi, PradeepkiranTeixeira, Sofia RodriguesJaniszewska-Turak, EmiliaTeixidó, JordiJankowska, MagdalenaTeixidó, MeritxellJanot, RaphaelTellez Jurado, LuciaJanzen, NilsTéllez-Téllez, MauraJara Palacios, María JoséTéllez-Valencia, AlfredoJara, PaulTellier, MichaelJaradat, NidalTello, JavierJarmuła, AdamTempera, GiannaJarosz, TomaszTeng, PengJarvis, KarynTenore, GianJasinski, MarcinTenorio, Manuel JimenezJasiński, RadomirTeodorescu, FlorinaJaśkowska, JolantaTeodori, ElisabettaJaudzems, KristapsTeramoto, HidetoshiJaumot, JoaquimTerekhova, Irina V.Jauregi, PaulaTerent’ev, AlexeiJayaraman, AnushaTerentyev, AlexanderJaźwiński, JarosławTeresa, Del Castillo-SantaellaJean, LudovicTerraneo, GiancarloJee, JunGooTerreni, MarcoJeffery, Constance J.Tersariol, Ivarne L. S.Jeffryes, ClaytonTerzaghi, William BryanJeleń, HenrykTerzopoulou, ZoeJeleń, Henryk H.Tesic, ZivoslavJelesko, John G.Tesnière, Catherine M.Jelonek, KatarzynaTetreau, GuillaumeJembrek, Maja JazvinšćakTey, Siew LingJendelova, PavlaTezuka, YasuhiroJendrzejczyk-Handzlik, DominikaThakurela, SudhirJenett-Siems, KristinaThamattoor, Dasan M.Jeng, Dong-ShengThang, Tran QuyetJenkins, IanThanigaimani, ShivshankarJenkins, Samir V.Tharmalingam, NagendranJensen, MikaelThavasyappan, ThambiJenssen, HåvardTheile, DirkJeon, Byeong HwaTheodorakis, Emmanuel A.Jeon, JonghoTheodorou, GeorgosJeon, SookyoungTheoduloz, CristinaJeon, Sung HoThéron, LaëtitiaJeon, Tae-ilThibault, JulesJeong, Byung-HoonThiel, WernerJeong, DaewonThiem, BarbaraJeong, Lak ShinThiéry, ValérieJeong, Seong HoonThil Smith, Adam AlexanderJeong, Seon-YongThilakarathna, Surangi H.Jepsen, JulianThirunavukarasu, DeepakJeremic, Aleksandar M.Thiruvengadam, MuthuJerkowić, IgorThomas, Dr. RaymondJeromel, AnaThomas, OlivierJeschke, MarcThomas, T.J.Jessen, HenningThompson, Karl ManleyJessop, Philip G.Thompson, Miles D.Jetten, AntonThompson, RichardJha, Ramesh K.Thorat, NanasahebJi, TuoThreadgill, MichaelJia, KailiangThrivikraman, GreeshmaJia, LiThyagarajan, BaskaranJiang, ChengTian, Fang-BaoJiang, DianluTian, Shung WuJiang, FuyiTian, YuanJiang, LiuweiTibullo, DanieleJiang, NanTietel, ZiporaJiang, XiqianTietjen, IanJiang, XuchuanTietze, AlesiaJiang, XuefengTietze, RainerJiang, XuntianTikhonov, VladimirJiang, ZaixingTimlin, Jerilyn A.Jiang, ZiwenTimoshenko, AlexanderJiao, KuiTimperio, Anna MariaJijie, RoxanaTing, Jeffrey M.Jiménez Arellanes, AdelinaTing, Richard C. H.Jimenez Migallón, AlfonsoTiperciuc, BrindusaJiménez, CarlosTischler, DirkJiménez, JuanTisi, RenataJimenez, M. AngelesTitov, Aleksei A.Jiménez-Aparicio, ReyesTiwari, Rakesh K.Jiménez-Araujo, AnaTIXIER, Anne-SylvieJiménez-Aspee, FelipeTiziani, StefanoJimeno, CirilTlak Gajger, IvanaJin, Chang HyunTlili, AnisJin, Guo-XinTobrman, TomášJin, Hyeong MinToczylowska-Maminska, RenataJin, LiTodoroki, YasushiJin, MingTodorova, DessislavaJin, TengchuanTofani, DanielaJirovetz, LeopoldToffoli, DanieleJo, ChangbumToiu, AncaJo, HyunilTokatly, IlyaJobling, MalcolmToldy, AndreaJofre-Reche, Jose A.Tolomelli, AlessandraJohn, ŁukaszTolosa, JosefaJohn, ShaliniTolvanen, Martti E.E.Johnson, Samuel A.Tomanek, BoguslawJohnston, Linda J.Tomas, AlejandraJohnston, Michael J. W.Tomasini, ClaudiaJokić, StelaTomaz, Cândida TeixeiraJones, CarolTomaz, IvanaJones, HuwTomczykowa, MonikaJones, MarjorieTomi, FélixJones, Owen GriffithTomić, SimonidaJoo, Hong-GuTomovska, RadmilaJoo, TaihaTomšič, BrigitaJoos, JonasTomuta, IoanJordão, António ManuelTondi, DonatellaJorgensen, FlemmingTondo, MireiaJørgensen, Kåre B.Tonelli, MicheleJose, JineyToniolo, LuanaJoshi, Pushpa RajToorie, AnikaJoshi, SanketTopin, JeremieJoshi, TanmayaTopoglidis, EmmanuelJosue, DelgadoTopuz, FuatJothi, Palani RajaTorbeev, VladimirJotshi, ChandToribio, LauraJouvenot, DamienTorikai, KoheiJoven, JorgeTormo, José RubénJówko, EwaTornabene, FrancescoJu, Jyh-CherngTornesello, Anna LuciaJudson, Richard S.Toro, Roberta G.Juhász, BélaTorović, LjiljaJulian, FernandoTorrado, Juan J.Julio, Nogales-BuenoTorras-Ambròs, JoanJullian, NathalieTorres, CristianaJung, Byung JunTorres, MarielaJung, HaesungTorres, RodrigoJung, HeonTorres-Ramírez, EduardoJung, Hye JinTosaka, MasatoshiJung, HyunToscani, AnitaJung, In HwanTosheva, LubomiraJung, JaehanTosi, SolveigJung, Jong-WhaTositti, LauraJung, Kyung HwanTošner, ZdeněkJung, Kyung-HyeTóth, TündeJung, ManfredTotsingan, FilbertJung, SeunhoTouil-Boukoffa, ChafiaJung, Yong ChaeTowers, ChristinaJung, Young MeeToyama, IkuoJung, Young-SikToyoma, TadashiJurak, MałgorzataToyota, TaroJuranović Cindrić, IvaTrabocchi, AndreaJurcek, OndrejTrachtman, HowardJurczak, JanuszTrader, DarciJurečka, PetrTrakselis, Michael A.Jurić, MarijanaTrammell, SamJurikova, TundeTran, Anh NhatJurk, KerstinTran, TrongJurkowska, HalinaTrandafirescu, Cristina-MariaJuskowiak, BernardTrashin, StanislavJusticia, JoséTrcek, JanjaJuszczak, LesławTres, Marcus ViniciusJyothi, Rajesh KumarTresserra-Rimbau, AnnaK. Karabagias, IoannisTretyakov, Evgeny V.Kabelác, MartinTrevisan, AndreaKabir, AbuzarTriaca, VivianaKacaniová, MiroslavaTrievel, RayKachamakova-Trojanowska, NeliTrilleras, JorgeKachrimanidou, VasilikiTrinchera, MarcoKącka-Zych, AgnieszkaTrincone, AntonioKaczmarek, HalinaTrindade, HelenaKaczor, Agnieszka A.Trindle, CarlKaczor, GrzegorzTringali, CorradoKaczorek, EwaTrinh, ThuatKadam, AvinashTriola, GemmaKadela-Tomanek, MonikaTripathi, DiwakerKadokawa, Jun-ichiTripathi, Lokesh P.Kadota, IsaoTripodi, GianlucaKageshima, YosukeTripodo, GiuseppeKageyama, HakutoTrivedi, Evan R.Kagiya, TadayoshiTrivedi, VivekKagota, SatomiTroise, Antonio DarioKairys, VisvaldasTroppmair, JakobKaiser, AnnetteTrotsko, NazarKaji, NoritadaTrouillon, RaphaëlKajimoto, ShinjiTrovato, FabioKajiya, HiroshiTrujillo, Antonio-JoséKakiuchi, FumitoshiTrujillo, CristinaKakkar, AshokTrujillo-Cayado, LuisKálai, TamasTrujillo-Rodríguez, María JoséKalani, KomalTrukhanov, Sergei ValentinovichKalas, WojciechTrusso Sfrazzetto, GiuseppeKalayda, GannaTruzzi, CristinaKalchenko, VyacheslavTrzeciak, AnnaKalemba, DanutaTsafantakis, NikolaosKalhapure, Rahul S.Tsai, Chen-YenKalíková, KvětaTsai, Jen-ChiehKalíková, KvӗtaTsai, Mei-LinKalinina, OlgaTsai, Wan-YuKalinowska Lis, UrszulaTsakovska, IvankaKalinowska, MonikaTsaltas, DimitrisKaliva, MariaTsarkova, Larisa A.Kaliyan, NalladuraiTsatsanis, ChristosKALLITHRAKA, StamatinaTsay, CHien-YieKallitsis, Joannis K.Tscheliessing, RupertKalogianni, DespinaTschentscher, RomanKalogiannis, Konstantinos G.Tseng, Chih-HuaKalogirou, Andreas S.Tseng, Wei-LungKaltner, HerbertTsenkova, RoumianaKalyana Sundaram, Ramalingam VenkatTshuva, Edit Y.Kamachi, ToshiakiTsigelny, Igor F.Kamal, Muhammad ShahzadTsimidou, MariaKamaly, NazilaTsiolaki, Paraskevi L.Kamatham, NareshbabuTsirelson, Vladimir G.Kamato, DanielleTsitsilonis, Ourania E.Kamdem, DonatienTso, Chi YanKamel, KarolTSO, Edwin Chi-YanKameo, HajimeTsoi, James Kit-honKameshima, SatoshiTsoupras, AlexandrosKamiloglu, SenemTsubaki, KazunoriKamińska, GabrielaTsuchimoto, TeruhisaKaminski, DanielTsuchiya, YuichiKamysz, ElżbietaTsuji, HidetoKamysz, WojciechTsukahara, ToshifumiKamzolova, SvetlanaTsumoto, KouheiKan, Chi-waiTsunoda, MakotoKanai, MasashiTsutsumi, OsamuKananovich, Dzmitry G.Tubaro, CristinaKanazawa, ArihiroTuccinardi, TizianoKanczler, JanosTucker, StevenKanda, NaokoTucker, Thomas J.Kandylis, PanagiotisTudela, JoseKaneko, GenTugizimana, FideleKanetis, LoukasTukhbatullin, AdisKang, ChulhunTulkens, PaulKang, CongBaoTuller, TamirKang, DaeshikTullio, VivianKang, DongchulTullius Scotti, MarcusKang, Hee EunTůma, KarelKang, Hee Y.Tung, Chih-KuanKang, InhaeTunick, MichaelKang, Jae SeungTunon, InakiKang, Jeon WoongTuorto, FrancescaKang, JonghoonTuovinen, OlliKang, Sang SunTupally, Karnaker R.Kang, SinyoungTurhanen, PetriKang, Sun ChulTurk, TomKang, WonkuTurkowski, Volodymyr M.Kangas, HeliTurku, AinoleenaKanie, OsamuTurnbull, GrahamKanno, TakahiroTuttolomondo, AntoninoKanno, YosukeTuvdendorj, DemidmaaKano, NaokiTuzimski, TomaszKantminienė, KristinaTyliszczak, BożenaKantono, KevinTymecki, LukaszKao, Chai-LinTymecki, ŁukaszKapcia, Konrad JerzyTzanavaras, Paraskevas D.Kaplan, BarbaraTzen, Jason T.C.Kapsalis, Vasilios C.Tzeng, Biing-ChiauKapusta, IreneuszTzeng, Nian-ShengKarabaliev, MiroslavTziveleka, Leto AikateriniKarakashev, DimitarTzortzakis, NikosKarakashev, StoyanTzschucke, ChristophKarakasidis, TheodorosUbeda, CristinaKaralis, VangelisUccelletti, DanielaKaramac, MagdalenaUchiyama, YasuoKaraman, SinemUddin, JamalKaraś, MagdalenaUebanso, TakashiKarasawa, KenUebe, RenéKarcz, DariuszUeda, TadaharuKarczmarzyk, ZbigniewUemiya, ShigeyukiKardos, JózsefUllevig, SarahKareiva, AivarasUllrich, MatthiasKarelson, MatiUmehara, TakashiKarim, NazmulUmerska, AnitaKarkanis, AnestisUnderwood, Mark D.Karmali, AminUnno, KeikoKARPIŃSKI, TOMASZ MUno, TomohideKarpinski, Tomasz M.Unterlass, MiriamKashman, YoelUpadhyaya, JasbirKaškonienė, VilmaUpare, Pravin P.Kasnatscheew, JohannesUrakawa, OsamuKasner, Margaret T.Urban, MilanKasprowicz, DobrosławaUrban, PawelKaszewski, JarosławUrbanc, BrigitaKataev, EvgenyUrbanczyk-Lipkowska, ZofiaKataoka, RyotaUri, AskoKatarzyna, MalarzUriarte, EugenioKatayama, ShigeruUrra, FelixKathuria, HimanshuUrzua, AlejandroKatikou, PanagiotaUsami, YoshihideKato, EisukeUshijima, KentaroKato, MasayaUsui, KenjiKato, YasumasaUsuki, ToyonobuKatoh, ToshihikoUsuki, YoshinosukeKatsila, TheodoraUversky, VladimirKatsoris, PanagiotisUziel, OritKatsoulas, NikolaosV Ivanov, AlexanderKatsuta, ShoichiV. González López, CynthiaKattamuri, PadmanabhaVaara, MarttiKatyal, PriyaVairetti, MariapiaKatz, Howard E.Vakros, JohnKatz, LeonardValant-Vetschera, KarinKaur, AmandeepValcarcel, JesusKaur, HarleenValdes-Lopez, OswaldoKaushik, NagendraVale, NunoKavadiya, ShalineeVale, PauloKavan, LadislavValentao, PatriciaKawabata, KyuichiValente, LigiaKawade, KensukeValenti, MariaKawaguchi, Shin-ichiValentová, KateřinaKawakami, SusumuValentovic, MonicaKawakita, HidetakaValenzuela, RodrigoKawamura, AkiraValenzuela, SusanaKawamura, IzuruValiante, VitoKawamura, TomoyukiValiullin, RustemKawano, Daniel FábioValko, MarianKawano, KouichiroVallejo, Javier P.Kawa-Rygielska, JoannaVallejos, CarlosKAWASAKI, HiroshiVallet, ValérieKawashima, TakayukiValletta, AlessioKawiak, AnnaValoppi, FabioKayano, Shin-ichiValverde, IbaiKayumov, AiratVamanu, EmanuelKearsey, StephenVan Bennekom, W.P.Keglevich, GyörgyVan Berkel, WillemKeijzers, GuidoVan Bommel, Maarten R.Kéki, SándorVan Cutsem, PierreKeller, AdrianVan Den Berg, Marco AlexanderKeller, JohannesVan den Bergh, HubertKeller, SandroVan Der Meulen, Nicholas P.Kello, MartinVan Der Schaaf, UlrikeKellogg, Joshua J.Van Dyke, Michael W.Kemp, Michael G.Van Herwijnen, HendrikKennedy, EileenVan Laar, TriciaKenny, Hilary A.Van Lis, RobertKensler, Thomas W.Van Maarseveen, JanKeppler, JuliaVan Waardenburg, RobertKerch, Garryvan Wullen, LeoKerman, KaganVance, DavidKerr, PhilipVanderlei, Maria De FátimaKersten, RolandVanella, LucaKertmen, AhmetVannini, AndreaKerwin, Sean M.Vanoli, MaristellaKeseru, GyorgyVanoni, Maria AntoniettaKessel, DavidVanthuyne, NicolasKetnawa, SunanthaVányolós, AttilaKetoja, JukkaVáradi, GyörgyiKeyzers, RobVáradi, JuditKeyzers, RobertVarga, SzabolcsKhalil, ZeinabVargas Jentzsch, PaulKhalymbadzha, Igor A.Varghese Gupta, SheebaKhan, MerajuddinVarikuti, SanjayKhan, Mohammad EhtishamVarley, RussellKhan, MohsinVarma, Rajender S.Khan, Muhammad JamalVarna, MarianaKhan, MujeebVaroni, Elena MariaKhan, NaghmaVarotsis, ConstantinosKhandavilli, UdayaVarriale, AntonioKhanfar, Monther A.Varrot, AnnabelleKhasainov, Boris A.Varvounis, GeorgeKhasawneh, FadiVasas, AndreaKhatib, SolimanVasdev, NeilKhattab, MuhammadVashurin, ArturKheradpezhouh, EhsanVasic, VesnaKhlebnikov, Andrei I.Vasilache, VioricaKhodadadi, Jay M.Vasile, CorneliaKhoo, Kay-HooiVasile, FrancescaKhoshnood, AbbasVasiljević, JelenaKhotimchenko, Maxim Y.Vasilyev, AlexanderKhozin-Goldberg, InnaVásquez-Velásquez, DavidKhrustalev, VladislavVassaux, GeorgesKhung, Yit LungVassilev, Nikolay G.Khurshid, ChrowVauthey, EricKhyzhun, O.Yu.Vaverkova, MagdalenaKi, Sung HwanVaz Junior, SilvioKianifariz, MahnazVaz, JosianaKiba, AkinoriVazquez Belda, BeatrizKicel, AgnieszkaVázquez, José AntonioKida, MałgorzataVazquez, ManuelKidane, DawitVázquez, SantiagoKiec-Kononowicz, KatarzynaVázquez-Flota, FelipeKieliszek, MarekVázquez-García, Rosa AngelesKiemer, AlexandraVazquez-Martínez, José AlfredoKierys, AgnieszkaVázquez-Tato, M. PilarKijjoa, AnakeVazquez-Vazquez, OlallaKikowska, MałgorzataVeatch-Blohm, M.E.Kikuchi, HaruhisaVecchio Ciprioti, StefanoKikuchi, TakashiVédrine, JacquesKikuchi, TakeshiVeeramani, SureshKilár, AnikóVega Gutierrez, SarathKim, BongleeVehus, Tore SandnesKim, Byoung SikVeis, Deborah J.Kim, Chang-EopVejpravova, JanaKim, Chuk YoungVekariya, RakeshKim, Chun HoVelagapudi, RavikanthKim, Chu-YoungVelasco, PabloKim, ChyerVelasco-Torrijos, TrinidadKim, DaehwanVelaz, ItziarKim, Dae-YoonVeleeparambil, ManojKim, DokyoungVelezheva, Valeriya S.Kim, DongukVelić, NatalijaKim, Gue-HyunVelikova, TsvetelinaKim, HakwonVelitchkova, MayaKim, HangunVelkov, TonyKim, Hee-JoonVemula, HarikaKim, HoonVendrell, JosepKim, HyesunVenhuis, Bastiaan J.Kim, Hyeung-RakVenkatadri, RajkumarKim, HyoungsuVennerstrom, JonathanKim, Hyun JaeVentura-Juárez, JavierKim, Hyung SikVenturi, FrancescaKim, HyunwooVenugopal, DhamodharanKim, Il WonVenus, JoachimKim, In-JungVera, LuisaKim, Jae KwangVerardo, VitoKim, JaheonVerde, IgnacioKim, JeongkwonVerdejo, BegoñaKim, Ji YeonVerga, DanielaKim, Jin SuVergani, LauraKim, JoungmokVergara, DanieleKim, Jung-HoonVerhoeven, AdrieKim, Ki HyunVerhulst, AnjaKim, Ki SuVerlet, JanKim, Kil-SooVerly, Rodrigo MoreiraKim, KyobumVerma, Navin KumarKim, KyounghyunVerma, RanjitKim, Kyung BoVerma, Santosh KumarKim, LouVermeulen, Christiaan EtienneKim, Mi-hyunVermeulen, MarcKim, MinVerotta, LuisellaKim, Min-SooVersari, AndreaKim, Seok-HoVersino, ElisabettaKim, Seok-JhinVerza, Simone GasparinKim, Seong-GonVetter, MarcusKim, SeonghoVetter, Stefan W.Kim, Seong-KyunViapiana, AgnieszkaKim, Seung HyunVicent, CristianKim, Seung WookVictor, BrunoKim, Seung-HyunVidal, FernandoKim, Seung-JooVideau, PatrickKim, SeyunViegas, OlgaKim, Soo UnViennet, CelineKim, SoyounVigier, KarineKim, Sung WonVíglaský, ViktorKim, Sung-HoonVignolle, JoanKim, T. DoohunVijayavenkataraman, SanjairajKim, Tae KonViktorova, JitkaKim, YangVil’, Vera A.Kim, YoungmeeVila, CarlosKim, YoungsooVila, Fernando D.Kim, Young-SukVilardi, GiorgioKim, Yu ChulVilgis, ThomasKim, YunjeongVilla, ChiaraKim, YuriVilla, FedericaKimbaris, AthanasiosVilla, StefaniaKimura, HideoVillaverde, Juan JoséKimura, Ken-ichiVillegas, DolorsKimura, YoshiharuVillena, JoanKinder, DavidVilloutreix, BrunoKindl, MarijaViña Olay, JaimeKing, Chih-YenVinardell, MariaKinzhalov, Mikhail A.Viñas, MiguelKirillov, EvgueniVincent, DelphineKirk, AndrewVincenti, FlaminiaKirsch, Gilbert H.Vincenzo, De LeoKirton, Stewart B.Vinciguerra, ManlioKiselev, VitalyVinnik, DenisKishikawa, NaoyaVinogradov, VladimirKisková, TeréziaViola, ManuelaKiss, AnnaViollet, BenoitKiss, Nóra ZsuzsaVirjamo, VirpiKiss, RitaVirta, PasiKitagaki, HiroshiVirto, MailoKitahama, YasutakaVishe, MaheshKitajima, MarikoVisioli, FrancescoKitamura, AkiraViso, AlmaKitano, YoshikazuViswanathan, SowmyaKitchen, JonathanVital, Joaquim Silvério MarquesKlajner-Maculewicz, BarbaraVitalone, AnnabellaKlaus, AnitaVitetta, LuisKleczkowska, PatrycjaVittadello, MicheleKleczkowski, Leszek A.Vittal, Jagadese J.Kleier, Daniel A.Vlachova, ViktorieKlein, Andrés D.Vladimir, KalininKlein, Lisa C.Vladimirov, Vladimir I.Kleinpeter, ErichVlainić, JosipaKlensporf-Pawlik, DorotaVlashi, ErinaKlepac, DamirVllasaliu, DritonKletsas, DimitrisVo, Duc-ThangKlewicka, ElzbietaVoca, SandraKlika, VaclavVocciante, MarcoKlimek, AnnaVockley, JerryKlimkowicz-Pawlas, AgnieszkaVodnar, Dan C.Kline, PaulVoellger, BenjaminKlochkov, Vladimir V.Vogel, ManjaKłodzińska, EwaVogel, PierreKlokov, DmitryVögele, MartinKlose, ThomasVoglmeir, JosefKlouda, LedaVoisset, CecileKlug, Dennis D.Vokurka, AlešKlupczynska, AgnieszkaVolf, IrinaKmiecik, DominikVoliani, ValerioKnapp, SpencerVolk, DavidKnez Hrnčič, MašaVolker, KörstgensKnežević, AnamarijaVolkova, ElenaKnezevic, JelenaVollbrecht, JoachimKnight, ChristopherVolmajer, JulijaKnittelfelder, OskarVon Wright, AtteKnudsen, GabrielVovk, TomažKnupfer, MartinVozzi, GiovanniKnupp, GerdVračko, MarjanKobayakawa, TatsuVranes, MilanKobayashi, KenichiVrontaki, EleniKobayashi, YayoiVrsic, StankoKobayashi, YuichiroVujicic, MiloradKobeissy, Firas H.Vuko, ElmaKobera, LiborVukovic, RosemaryKoboziev, Iurii YuriVuong, Quan V.Kobrak, Mark N.Vuong, Thu V.Koch, WojciechVuppala, SrikanthKochan, EwaVuts, JozsefKochan, KamilaVyas, DiwakarKochana, JolantaVyatkina, KiraKoda, TomokoVynios, DemitriosKodama, ShintaroVyviurska, OlgaKodogiannis, Vassilis S.Wacławek, StanisławKoga, FumitakaWada, RyuichiKoga, Jun-ichiroWadhwa, MeenuKoh, Cho YeowWadsak, WolfgangKoh, DavidWagner, AnkeKöhl, GudrunWagner, Bridget K.Koichi, KatoWagner, PawelKoikawa, MasayukiWahab, M. FarooqKoizume, ShiroWähälä, KristiinaKoizumi, Take-akiWajs-Bonikowska, AnnaKokado, KentaWakao, MasahiroKókai, ZoltánWaligorski, PiotrKokkinos, ChristosWalker, HeatherKokoska, LadislavWall Medrano, AbrahamKokotkiewicz, AdamWallner, BjörnKokotos, ChristoforosWalsby, CharlesKoksharova, Olga A.Walsh, KerryKolbe, JanaWalter, JeskeKoller, MartinWalton, ChristopherKolluru, GopiWalton, JohnKolobov, Alexandr AlexandrovichWan, ChaoKolodziejczyk, JoannaWang, Be-JenKolot, MikhailWang, BoKomaki, HisayukiWang, Bo-ChengKomati, RajeshWang, BujunKomatsu, MasanobuWang, Chao-MinKomatsu, ToruWang, Chih-ChiehKomis, GeorgeWang, Chin-KunKomives, TamasWang, ChuanlongKommireddy, VasuWang, DanhongKomorowicz, IzabelaWang, DavidKomov, Viktor TrophimovichWang, DongmeiKonar, ArkaprabhaWang, ErkangKonar, SumitWang, FazuoKonc, JanezWang, FeiKong Thoo Lin, PaulWang, Feng-ShengKong, Chang-SukWang, FupengKong, DuanyangWang, FuyiKong, WeiliWang, GaoxueKong, XianWang, Hao-ChingKong, XiangruiWang, Hay-Yan J.Kong, Xiang-TianWang, HsiuyingKonger, Raymond L.Wang, Huei-JuKonieczna, LucynaWang, HuiKonieczny, KrzysztofWang, Hui-minKonieczny, PiotrWang, JeffreyKönigs, Rene M.Wang, JiabinKonishi, MasaakiWang, JiaminKonno, HiroyukiWang, JianfengKonno, KatsuhiroWang, JianxuKonopacka-Łyskawa, DonataWang, JinKonstantinov, Spiro M.Wang, Julie Tzu-WenKontoyianni, MariaWang, JunKoo, Hee-beomWang, JunjieKooi Yeong, KhawWang, KaiKooistra, AlbertWang, KankanKootala, SujitWang, Lei (Queen’s University)Kopecký, VladimírWang, Lei (University of Minnesota System)Kopel, PavelWang, LianchunKopjar, MirelaWang, LinKoppel, KadriWang, LirongKopra, KariWang, Michael CaiKopsahelis, NikolaosWang, NingKorac, BatoWang, PengKorb, MarcusWang, PengchengKorchowiec, JacekWang, PingyuanKore, RajkumarWang, QingsongKoren, KlausWang, QiuanKorlyukov, Alexander A.Wang, QunKornath, AndreasWang, RuiyongKoronkiewicz, StanisławaWang, San-LangKorsching, EberhardWang, Shang-KweiKorving, LeonWang, ShengKorzhikov-Vlakh, ViktorWang, Sheng-YangKosakowska, OlgaWang, Shi-BinKosalec, IvanWang, Shin-WeiKosanovic, DjuroWang, ShixingKoschella, AndreasWang, ShuaoKoshino, HiroyukiWang, ShueKosicka, KatarzynaWang, ShuqiKosik-Bogacka, DanutaWang, ShyiwuKosinski, PawelWang, SongcanKosinsky, Robyn LauraWang, SonglinKosmala, MonikaWang, TaiKosman, JoannaWang, TaoKošmrlj, JanezWang, TingKosters, AstridWang, WeiqunKostić, Aleksandar Ž.Wang, WeizhiKostin, Gennadiy A.Wang, WentianKostoglou, NikolaosWang, XiangKosuge, YasuhiroWang, Xiao YongKoszelnik, PiotrWang, XiaodongKot, BarbaraWang, Xiaoguang (William)Kotek, JanWang, XiaopingKotlowski, RomanWang, XinKotlyar, Alexander B.Wang, XingyuanKotora, MartinWang, XizuKotormán, MártaWang, XuKotowska, UrszulaWang, XuefengKotula-Balak, MalgorzataWang, XuejuKoufaki, MariaWang, XutongKoukounaras, AthanasiosWang, YiKoulen, PeterWang, YiboKounovsky-Shafer, KristyWang, YifeiKouremenos, Konstantinos A.Wang, YingKoutahzadeh, NeginWang, YongqiangKoutakis, PanosWang, YuKoutelidakis, Antonios E.Wang, Yun-MingKouza, MaksimWang, YupingKovačević, DavorWang, ZenengKovačević, StrahinjaWang, ZhaoKovacs, LajosWang, ZhaohuiKovács, ZoltánWang, ZheKoval, AlexeyWang, ZhenKovalenko, Galina A.Wang, ZhihanKovalenko, SergeyWang, ZhisongKovanecz, IstvanWang, ZhuoKovvasu, Surya PrakasaraoWanner, JuergenKowalczewski, PrzemysławWardill, Hannah R.Kowalczuk, MarekWardowska, AnnaKowalczyk, AgataWarnken, ZacharyKowalkowski, TomaszWarren, J. DavidKowalska, EwaWarren, Warren S.Kowalska, KatarzynaWasan, EllenKowalski, RadosławWaser, MarioKoyioni, MariaWasilewski, TomaszKozakiewicz, AnnaWasylka, Justyna PłotkaKozelka, JiriWatanabe, KazuhisaKozhukhova, Natalia IvanovnaWatanabe, NaoharuKoziróg, AnnaWaterlot, ChristopheKozliak, Evguenii I.Watkins, Gordon LeonardKozlova, ElenaWatrelot, Aude AnnieKozłowska (Kozlowska), MariolaWatts, PaulKracht, MichaelWebb, IanKragović, MilanWebba Da Silva, MateusKrahe, JamesWebber, MarkKrähenbühl, StephanWeber, CameronKrajewska, BarbaraWeber, DanielKrajka-Kuźniak, ViolettaWeber, JohnKrämer, Stefanie-DorotheaWeeks, BrandonKrammer, Eva-MariaWei, CunxuKranjac, MarinaWei, GangKrappmann, DanielWei, Guor-JienKrasavin, MikhailWei, Hong-YuKraševec, NadaWei, HuaKrátký, MartinWei, Jennifer N.Kraus, ChristophWei, KayaKrause, JeffreyWei, KongchangKrauze-Baranowska, MiroslawaWei, RenboKrawczyk, PrzemysławWei, Wei (Stanford University)Krchnak, ViktorWei, Wei (Washington State University)Krebsz, MelindaWei, ZhaojunKregiel, DorotaWei, ZhengKremer, LaurentWeigand, JuliaKřen, VladimirWeindl, GuentherKrick, StefanieWeinhold, ArneKrisch, JuditWeitkamp, Joern-HendrikKritchenkov, Andreii S.Welch, AlanKrivetskiy, ValeriyWelker, MarkKrivovichev, VladimirWen, JianjunKrol, EwelinaWen, XiaoyanKról, EwelinaWen, XuesongKról-Kilińska, ŻanetaWen, Zhi-HongKruger, CherieWen, ZhiweiKruszynski, RafalWende, KristianKrylov, IgorWende, WolfgangKrylov, Sergey N.Weng, Ching FengKrystyna, Skalicka - WoźniakWeng, XiaoyuKrzaczek, PawełWenzel, BarbaraKrzanowski, MarcinWerlé, ChristopheKrzyściak, WirginiaWesełucha-Birczyńska, AleksandraKrzywonos, MałgorzataWesołowski, MarekKu, SeockmoWestcott, Stephen A.Ku, Yang-GyuWestmark, CaraKuan, Yu-HsiangWestover, Andrew SKubala, LukášWestphal, AdrieKuban-Jankowska, AlicjaWhitfield, PhilKubatka, PeterWhitten, SteveKubiak, PiotrWia̧cek, Agnieszka EwaKubica, PawełWianowska, DorotaKubíček, VojtěchWibowo, DavidKubicova, LenkaWichert, MorenoKubinová, RenataWiczk, WiesławKubinova, SarkaWiczkowski, WieslawKubiński, KonradWiddifield, CoryKubinyi, MiklósWidhalm, MichaelKubo, AtsushiWie, Jeong JaeKubo, TakanoriWieczorek, MarcinKubota, TakaakiWieczorek, Władysław G.Kuca, KamilWiedman, GregoryKuča, KamilWientjes, EmilieKucerova, MartaWigle, JeffreyKucharska, AlicjaWijethunga, TharangaKucharska, KarolinaWiktorska, KatarzynaKucinska-Lipka, JustynaWilding, MatthewKucinski, KrzysztofWilds, Christopher J.Kucuk, IsrafilWilk, AleksandraKuczera, KrzysztofWilk, PiotrKudaibergenov, SarkytWilkins, HeatherKudelko, AgnieszkaWilkinson, JennyKudłak, BłażejWillberg-Keyriläinen, PiaKudlek, EdytaWilletts, AndrewKudo, FumitakaWilliams, BarbaraKudo, YutaWilliams, VanceKudr, JiriWills, MartinKudryashov, Sergey I.Wilson, Anne M.Kueppers, StephanWilson, Danny WKuhnle, GunterWilson, DavidKujawa, JoannaWilson, Jeffrey M.Kujawska, MałgorzataWilson, Justin J.Kujawski, WojciechWilson, StevenKukli, KaupoWiltschi, BirgitKuklja, MaijaWimmer, ZdenekKukula-Koch, WirginiaWinckler, ThomasKula, SławomirWinkelstern, IanKulak, NoraWinkler, JohannesKulczyński, BartoszWinkler, LarsKulišić Bilušić, TeaWińska, KatarzynaKulkarni, KetavWinter, AndreasKulkarni, ShashankWinum, Jean-YvesKulnitskiy, Boris A.Wiraja, ChristianKumamoto, EiichiWisian-Neilson, PattyKumar Patel, SravanWitonska, IzabelaKumar, AmitWlaź, PiotrKumar, AshutoshWłoch, MarcinKumar, AvneeshWłodarczyk, MaciejKumar, BinodWlodarczyk, RenataKumar, BrajeshWłodarczyk-Stasiak, MarzenaKumar, KrishanWnorowski, ArturKumar, PankajWöhnert, JensKumar, PenmetchaWójciak-Kosior, MagdalenaKumar, PradeepWojciechowski, KamilKumar, PrashantWojcieszak, RobertKumar, PraveenWójcik, GrzegorzKumar, RaviWojdyło, AnetaKumar, SandeepWojnarowicz, JacekKumar, VipinWójtowicz, AgnieszkaKumar, Vivek A.Wojtowicz, WojciechKumazawa, ShigenoriWojtunik-Kulesza, Karolina A.Kumirska, JolantaWojtyczka, RobertKummer, UrsulaWołejko, ElżbietaKundakovic-Vasović, TatjanaWölfle, UteKung, Chung-WeiWollweber, JuergenKunz, HorstWołosiak, RafałKuo (Marie), Meng-IWołowiec, StanisławKuo, Chia-HungWong, AlanKuo, Dong-HauWong, Chi-MingKuo, Pei-YuWong, ChungKuo, Ping-ChungWong, Clarence T. T.Kuo, Yao-HaurWong, James Tsz FungKupcewicz, BogumiłaWong, Ka-LeungKuppusamy, ManiselvanWong, Man-SauKurata, YoheiWood, ScottKurczab, RafalWood, Troy D.Kureha, TakumaWoodcock, BenKurek, MarcinWoolbright, Benjamin L.Kurgan, LukaszWoolf, Eric J.Kurka, OndrejWorek, FranzKurlyandskaya, Galina V.Wöstemeyer, JohannesKuroboshi, ManabuWoszuk, AgnieszkaKuroda, ChiakiWoźniak, ŁukaszKuroda, MasaharuWozniak-Knopp, GordanaKuroda, YoshiyukiWragg, David S.Kurrikoff, KaidoWright, AmyKurtán, TiborWright, SandraKus, PiotrWróbel, TomaszKuś, PiotrWrobel, Tomasz M.Kusano, ShuheiWrześniok, DorotaKustov, LeonidWu, Chang-YiKustov, Leonid M.Wu, Chi-ReiKustrowski, PiotrWu, ChunKutikhin, Anton G.Wu, ChungChunKuwata, ShigekiWu, Chung-HsinKuzuya, AkinoriWu, DiKvasnica, MiroslavWu, GongweiKwak, Jong HwanWu, HongjingKwak, SeonyeongWu, HongliKwasniewski, Misha T.Wu, Hung-ChinKwiecień, IwonaWu, Jing-YunKwok, Hang Fai (Henry)Wu, Jin-HuKwolek, PrzemysławWu, Jin-YiKwon, Hyung JooWu, KaiyuKwon, Oh SeokWu, KejunKwon, OranWu, Li-chenKwon, Soon-HongWu, LiliKwon, Young-WanWu, MaochunKyei, George BWu, Ming-ChungKyselka, JanWu, NanKyzas, George Z.Wu, Sheng YunLa Clair, JamesWu, Shih-HsiungLa Flor, Silvia DeWu, Shu-PaoLa Motta, ConcettinaWu, SiLa Regina, GiuseppeWu, Ta YeongLa Rocca, Renato V.Wu, WeiLa Rosa, CarmeloWu, XiaofengLa Russa, DanieleWu, XiaohuaLa Spina, RitaWu, Xin-PingLa, Duc DuongWu, Yi-XianLabbozzetta, ManuelaWu, Yuh-LinLabesse, GillesWu, YunlongLabruna, GiuseppeWu, Yu-TseLacaille-Dubois, Marie-AlethWu, ZhanhongLachowicz, Joanna IzabelaWujec, MonikaLachowicz, SabinaWunderlich, GerdLachowiec, JenniferWuttke, StefanLachter, Elizabeth R.Wyganowska-Świątkowska, MarzenaLacoste, ClémentXavier, Cristina Pinto RibeiroLafanechere, LaurenceXi, XinpingLafond, MickaëlXi, ZhengxiongLaforenza, UmbertoXia, GuanglinLaganà, Antonio SimoneXian, MingLaghi, LucaXiang, YuanLago, Ana B.Xiao, AnfengLaguna, O.H.Xiao, DequanLahav, OriXiao, KaijunLahem, DrissXiao, RuiyangLai, Chin-HungXie, HongfengLai, QinghuaXie, LanLai, YanhaoXie, LishiLakatos, AkosXie, QingmeiLakatos, LorantXie, WankunLakshmanan, ImayavarambanXie, WenyanLalas, StavrosXie, Zhong-RuLalatsa, AikateriniXimenes, ValdecirLam, KevinXin, DongyueLam, Kim-HungXin, JiayuLam, Sio-HongXing, FeiLamac, MartinXiong, WeiLamas, AlexandreXodo, LuigiLamba, DorianoXu, DongLambrinidis, GeorgeXu, HongtaoLambropoulou, DimitraXu, JianLamela-Raventos, Rosa M.Xu, JianweiLamm, Monica H.Xu, JiaxiLamprou, Dimitrios A.Xu, JiminLan, LanXu, JunLanc, DomagojXu, Jun-LiLanças, FernandoXu, KaikaiLancelin, Jean-marcXu, LinruLandeka Dragičević, TibelaXu, LuLandi, MarcoXu, PengfeiLang, DiXu, WenyanLang, IngeborgXu, XiaomingLangowski, Horst-ChristianXu, XuesongLannoy, DamienXu, Yan (Ramapo College of New Jersey)Lanza, GiuseppeXu, Yan (University of Miyazaki)Lanzi, MassimilianoXu, YifeiŁapiński, AndrzejXu, Yi-JunLaquintana, ValentinoXu, YitingLaraia, LucaXuan, Tran DangLara-Sanchez, AgustinYabuki, SoichiLariccia, VincenzoYadav, MithileshLaroche, CélineYakura, TakayukiLaronze-Cochard, MarieYakymovych, IhorLash, Timothy D.Yallapu, MuraliLasik-Kurdyś, MałgorzataYamada, ShigeyukiLaskowska, MagdalenaYamada, ShuheiLászló, KrisztinaYamada, SohsukeLatacz, GniewomirYamagishi, TakehiroLatorre-Moratalla, Mariluz LuzYamaguchi, EijiLatosińska, Jolanta NataliaYamaguchi, FumioLattanzio, RossanoYamaguchi, MakotoLau, Woei JyeYamaguchi, TakumiLauberts, MarisYamamura, SoichiroLauder, BobYamanaka, MasamichiLauren E., BrownYamanaka, ShusukeLaurent, FERRIÉYamashita, AkiraLaurenti, EnzoYamato, TakehikoLaurie, FelipeYamauchi, SatoshiLaurienzo, PaolaYan, DayunLaurita, RomoloYan, JingLavandera, IvanYan, LifengLavecchia, RobertoYan, ShengLavilla, RodolfoYan, XiaohuiLavogina, DarjaYanagawa, AyaLaw, Ga-LaiYanagi, TerukiLawrence, KarlYanda, MuraliLázár, IstvánYáñez-Sedeño, PalomaLazzara, GiuseppeYang, C.-F.Lazzarato, LorettaYang, Chao-HsunLazzari, ElisaYang, ChengLe Bourvellec, CarineYang, Chii-RongLe Gall, ThierryYang, Chul-SuLe Grognec, ErwanYang, Chun-LongLe Joncour, VadimYang, Ding-YahLe Questel, Jean-yvesYang, DongLe, Anh VYang, Feng-LingLe, ZaiyuanYang, Hang-ShengLeal, Ana MendesYang, HongshunLeardi, RiccardoYang, Hsiao-YuLeBlanc, GabrielYang, Hung-ChihLebleu, BernardYang, InhoLeblond, JeanneYang, JiaweiLebrun, VincentYang, JingLeclercq, AlexandreYang, KunLedesma, Ana EstelaYang, LeiLedeţi, IonuţYang, LijiangLedinsky, MartinYang, Shao-YuLednev, VasilyYang, Shun-ChinLedo, AnaYang, Si KyungLedwon, PrzemyslawYang, TingbinLee, Bong HoYang, WeiLee, BonggiYang, XiuweiLee, Chang HoonYang, XuanLee, ChangheeYang, Xue-MingLee, ChangwonYang, YannanLee, ChangWooYang, Ying-WeiLee, Che-HsinYang, Ying-YingLee, Chen-HsiangYang, YongkangLee, Chia-HungYang, Yu-ChiaoLee, Ching-KuoYANGUI, AymenLee, Chun-LinYao, Chao-LingLee, DongjooYao, YangLee, E-jianYaremenko, Ivan A.Lee, Geun-ShikYarlagadda, VenkataLee, GihyunYashchenok, AlexeyLee, Hian KeeYasuda, ShinLee, Hon ManYasui, KyuichiLee, Hong-KiYatsunyk, LiliyaLee, Hwa-JinYazaki, RyoLee, Hye SukYazhgur, PavelLee, IlkeunYe, AnpeiLee, In-ChulYE, EnyiLee, Jae WookYe, YunpengLee, JaehwiYedjou, Clement G.Lee, JeongmiYeh, Jwu-LaiLee, Jin-KooYeih, Wei-ChungLee, Jong-DaeYeliseev, AlexeiLee, Jun SeokYen, Feng-LinLee, Jun SikYen, Ming-ShienLee, Jung RoYeo, SynLee, JungwoonYepuri, Nageshwar R.Lee, JunseongYerabolu, JAYASUDHAN REDDYLee, Jun-SooYi, MyunggiLee, Kyu SupYi, YougenLee, Li-AngYi, Young-SuLee, Maw-RongYildirim, AdemLee, Meng-shiouYildirim, IlyasLee, Min WookYilmaz, MehmetLee, MinaYin, HengLee, Myung KooYin, KingsleyLee, RichmondYing, MingyaoLee, Sang GilYohda, MasafumiLee, Sang KookYokomatsu, TsutomuLee, Sang MiYokosuka, AkihitoLee, Sang-HanYokosyo, KengoLee, Seok JaeYokota, ShingoLee, Shiow-JuYokota, Shin-ichiLee, Shyi-LongYoneda, ToshiyukiLee, SooyeunYoo, DongwonLee, SunwooYoon, In SooLee, Tae-KwonYoon, JinhwanLee, VictorYoon, MinjoongLee, Wei-ChiehYoon, MinyoungLee, Yeon SunYoshida, JunLee, Yeon-JuYoshida, Nidia CristianeLee, Young-ChulYoshida, TakashiLee, Yun BinYoshida, YukikoLee, ZhenghongYoshido, AtsuoLee-Ruff, EdwardYoshikawa, TakuyaLefebvre, TonyYoshimatsu, MitsuhiroLeffler, HakonYoshimi, YasuharuLegentil, LaurentYoshimoto, MakotoLéger, BastienYoshimura, YuichiLeggio, AntonellaYoshinaga, MasafumiLehotsky, JanYoshinari, NobutoLehtonen, AriYoshioka, Ken-ichiLei, FanYoshioka, NaokiLei, XiujuanYou, Ren-InLei, ZhentianYou, XiaonaLeitão, AnaYounes, HusamLeitão, Jorge H.Young, MichaelLeitgeb, MajaYu, BinLeja, KatarzynaYu, Cheng-JuLekka, MalgorzataYu, ChenglongLelli, MorenoYu, Deng-GuangLemaire, MartinYu, FabiaoLemanowicz, MarcinYu, GuangliLemli, BeátaYu, HaifengLemon, ChristopherYu, HuaLen, ChristopheYu, Jae WoongLenaghan, ScottYu, JingangLenardão, EderYu, JornLenci, ElenaYu, KangLenik, JoannaYu, LuLente, GáborYu, Lu-GangLentini, GiovanniYu, MeihuaLeo, Chen HueiYu, MingfengLeón, Gerardo A.Yu, MinzhongLeonard, StephenYu, RongminLeone, AntonellaYu, Ruei-SungLeone, GemmaYu, ShibataLeong, Max K.Yu, WeilingLeon-González, Antonio J.Yu, XiaobinLeoni, AlbertoYu, Yan PingLeonidas, Demetres D.Yu, YanleiLeonori, DanieleYuan, BingLeón-Rivera, IsmaelYuan, Min-HaoLeonti, MarcoYuan, YaxiaLeonyuk, N.I.Yuan, ZhihongLeonzio, GraziaYuan, ZuanningLepczynski, AdamYuan-Tsung, ChenLepenies, BerndYuba, EijiLephart, Edwin D.Yucesan, GundogLepkova, KaterinaYue, LingLeporatti, StefanoYukl, Erik T.Lepore, MariaYurkevich, Nataliya V.Leppert, WojciechYurovskaya, Marina A.Lequin, OlivierYushina, Irina D.Les, FranciscoZabierowski, PiotrLesac, AndrejaZabłocka-Godlewska, EwaLeśniewicz, AnnaZabot, Giovani L.Lesov, IvanZaccarini, FedericaLesyk, Roman B.Zacchia, MiriamLeung, Chung-HangZacconi, FlaviaLeung, DuncanZacharis, Constantinos K.Leung, IvanhoeZagotto, GiuseppeLeung, KaHoZaguła, GrzegorzLeuteritz, AndreasZaharia, ValentinLevesque, CelineZahid, MalihaLevin, Oleg VladislavovichZaid, HilalLevine, MindyZaidi, SaheemLevine, Mindy S.Zajdel, PawełLevy, MichaellaZajíčková, ZuzanaLewandowicz, GrażynaZakoldaev, Roman A.Lewandowski, WiktorZakrzewska, MalgorzataLewinska, AnnaZakrzewski, JerzyLewkowski, JaroslawZaleśny, RobertLewkowski, Jarosław A.Zaltariov, Mirela-FernandaLgaz, HassaneZamaratskaia, GaliaLhiaubet, Virginie LyriaZambrano, Maria De La LuzLi, Chia-YangŻamojć, KrzysztofLi, Dr. ZijianZamudio-Rivera, Luis S.Li, Fu-shuangZamyatnin, AndreyLi, GuangmingZang, LiqingLi, GuohuiZapata, CarlosLi, HongchunZaprutko, LucjuszLi, Hou-JinZAQUEN, NeomyLi, HuabinZarabadi-Poor, PezhmanLi, JianZarkovic, NevenLi, JinganZarra, IgnacioLi, JunshengZarrelli, ArmandoLi, KefengZarzycki, Pawel K.Li, Kuo-BinZavattaro, ElisaLi, LixinZavriev, Sergey K.Li, MeichunZawada, KatarzynaLi, Mei-LingZayas, BeatrizLi, MingqiangZbancioc, GheorghiţăLi, NaZdanowicz, MagdalenaLi, PingZdarta, JakubLi, PingyaZdzisińska, BarbaraLi, Po-HsienZebib, BacharLi, RenfengZeeshan, MuhammadLi, Ruei-NianZeiner, MichaelaLi, ShihaoZeković, ZoranLi, ShimingŻelaszczyk, DorotaLi, ShitaoZeleny, ReinhardLi, ShuangZeman, SvatoplukLi, SihengZemková, ErikaLi, TianhuZeng, MinxiangLi, WangZeng, ShuwenLi, Wen-WuZeng, TaoLi, WenyueZepeda, Rossana C.Li, XianZerbe, PhilippLi, XiangZerva, AnastasiaLi, Xiang-GuoŻesławska, EwaLi, XianglinZgórka, GrażynaLi, XiaoZha, GaofengLi, XinyongZhai, KuiLi, YanZhan, PengLi, Yeou-FongZhang, Bo (USA The Ohio State University)Li, YifanZhang, CanyangLi, Yi-FangZhang, ChunhuiLi, YingZhang, ChunyongLi, YiwenZhang, DimingLi, YouxinZhang, ErshuaiLi, YuanzuoZhang, FanLi, YunfeiZhang, GuannanLi, ZhaohuiZhang, GuanshiLi, ZhengqiangZhang, HongqiaoLi, Zhi-MinhgZhang, HuaLi, ZhongweiZhang, HuaweiLiagre, BertrandZhang, Jian-TingLian, HailanZhang, JinnanLiang, GuodongZhang, JunboLiang, Po-HuangZhang, JunjieLiang, ShuangZhang, Jun-LongLiang, YanZhang, JunzengLiang, YongZhang, KaiLiang, YuerongZhang, Ling-juanLiang, ZhibinZhang, MingzheLiao, Ling-HsiuZhang, QiaobaoLiao, PengZhang, QinghaiLiao, WupengZhang, Qing-WenLiao, Yi-JenZhang, RuiyongLiao, ZhihuaZhang, RunLiapi, CharisZhang, ShaozhongLiberato, FerraraZhang, ShupingLiberto, EricaZhang, TaoLicausi, FrancescoZhang, Wei (Nanjing University of Science and Technology)Licea, AlexeiZhang, Wei (Soochow University)Lichtenberg-Kraag, BirgitZhang, Wei (USA, Washington University in St. Louis)Lidon, FernandoZhang, WeicaiLiebman, JoelZhang, WujieLiem, Djin GieZhang, XiangLih-Ming, YiinZhang, XianghuiLilge, LotharZhang, XiaoxingLim, DaewoonZhang, XuLim, Hye-SunZhang, XuanjunLim, Jae HyangZhang, XuefengLim, Ji-HongZhang, XuexinLim, SoyeonZhang, YinglaoLim, Sung-JigZhang, YixiangLim, Sun-HyungZhang, YixuanLima Do Monte-Neto, RubensZhang, YoumingLima, BeatrizZhang, YuLima, DênisZhang, YugangLima, Fabio H. B.Zhang, YumiaoLima, Giuseppina P.P.Zhang, YunkaiLima, Rita De Cássia LemosZhang, YunyanLima, Sofia CostaZhang, ZhanhuiLimbeck, AndreasZhang, ZhenbinLimnios, DimitrisZhang, ZhenhuaLimongi, TaniaZhang, ZhibingLin, B.Y.Zhang, ZhiyuanLin, Chia-HerZhao, BinLin, Chih-LiZhao, GuohuaLin, Chung-HoZhao, JianzhangLin, Chun-LiangZhao, JidongLin, HaishuZhao, Jin-HaoLin, Hai-ShuZhao, LianfengLin, HongjunZhao, LihuaLin, Kuo-ShyanZhao, LiyanLin, LigenZhao, NingningLin, Mei-HsiangZhao, QianLin, Ming-KuemZhao, ShouLin, MingyueZhao, XuebingLin, Muh-ShiZhao, YadongLin, QishanZhao, YanLin, RijiaZhao, YanchuanLin, Shan-YangZhao, ZhenjiangLin, Xian-FuZhao, ZhenwenLin, XufengZhao, ZongminLin, Yi-LiZhaopeng, LiuLin, Ying-HungZhaowei, ZhangLin, Yuan-PinZhen, QiLin, ZekaiZheng, JiaLin, ZhiweiZheng, KaiLin, ZhoumengZheng, ShilongLin, ZongtaoZheng, XiaomingLindberg, GerrickZhi, ChunyiLindel, ThomasZhong, HaizhenLindemann, PeterZhong, JiangLinden, MikaZhong, Wei-ZhuLindoy, LenZhong, YuchengLinert, WolfgangZhou, ChuncaiLing, ChenZhou, HaiboLing, MauriceZhou, Heshan SamLinhari Rodrigues Pietro, Rosemeire CristinaZhou, HongyiLinko, VeikkoZhou, HuiLinscheid, Michael W.Zhou, JiaLinzaga-Elizalde, IrmaZhou, LiliLipiński, Piotr F. J.Zhou, QiLipka-Belloli, E.Zhou, QibingLisetskii, FedorZhou, QunLiszkowska, JoannaZhou, ShengbinLitti, LucioZhou, TengLitvić, MladenZhou, XiaochunLiu, BoZhou, XuLiu, Chao lienZhou, Yi-hongLiu, Chao-LinZhou, YonghuiLiu, ChengmeiZhou, YubinLiu, Chen-WuingZhou, ZheLiu, Cheuk LunZhou, ZhiLiu, ChuanjunZhu, BingLiu, DelongZhu, ChangfuLiu, FufengZhu, ChongyuLiu, FupengZhu, FengLiu, Gang (Institute of Microbiology, Chinese Academy of Sciences)Zhu, JianhuiLiu, Gang (The University of Texas)Zhu, JieLiu, Guang-YawZhu, LiangdongLiu, GuozhenZhu, MinghuiLiu, HongyuZhu, NingLiu, Jhy-ChernZhu, ShanLiu, JiZhu, WeiLiu, JieminZhu, WufuLiu, JunZhu, XiaoshanLiu, Jun-JenZhu, XuejunLiu, LeiZhu, Xu-HuiLiu, LiangliangZhuang, WeiLiu, ManingZhuang, XuliangLiu, Shing-HwaZhuang, YongliangLiu, Shiuh-TzungZhuravlev, VictorLiu, Shi-XiaZiarno, MałgorzataLiu, Shou-HengŽiarovská, JanaLiu, Shyh-JiunŽic, MarkLiu, SongpoZięba, AndrzejLiu, TaihongZiegler, ThomasLiu, TianyuZiegler-Borowska, MartaLiu, WenqiZielinska, BeataLiu, XiangrongZielińska, SylwiaLiu, XiaoboZielinska-Jurek, AnnaLiu, XiaoxiZieliński, MarcinLiu, XifengZieniuk, BartłomiejLiu, XinhuaZille, AndreaLiu, Xinyue (Sherry)Zille, MariettaLiu, XuqingZimba, PaulLiu, YajunZimmermann, BorisLiu, Yi-ChangZimoch-Korzycka, AnnaLiu, YingtaoZingg, Jean-MarcLiu, YuZinna, FrancescoLiu, Yuan-ShuaiZinnai, AngelaLiu, YuzhouZitka, OndrejLiu, ZhiZitko, JanLiu, ZhipingZiyatdinova, GuzelLiu, ZhixiaZizza, PasqualeLiverani, ElisabettaZloh, MireLiverani, LilianaZłotek, UrszulaLiWang, AndyZnosko, Brent M.Lizundia, ErlantzZobel, PatrickLjubenkov, IvicaZoccali, MariosimoneLleonart, RicardoZoidis, EvangelosLlop, JordiZoidis, GrigorisLloret, JulioŻołnowska, BeataLo Giudice, AngelinaZoltan-Istvan, SzaboLo Muzio, LorenzoZoppellaro, GiorgioLo Scalzo, RobertoZorc, BrankaLo, Yi-ChenZou, ChunbinLøbner-Olesen, AndersZou, GangLoboda, AgnieszkaZou, QuanLocardi, FedericoZou, WeiLocatelli, MarcelloZovko Končić, MarijanaLodyga-Chruscinska, ElżbietaZozulya, AlexeyŁodyga-Chruścińska, ElżbietaZubair, HaseebLofberg, AxelZubkova, OlgaLognay, GeorgesZubrik, AntonLohézic-Le Dévéhat, FrançoiseZucca, PaoloLohman, JeremyZuffo, MichelaLohning, AnnaZuk, MagdalenaLoira, IrisZukowski, WitoldLoiseau, PhilippeZuliani, Juliana PavanLoizides-Mangold, UrsulaZuluaga, PaolaLoizzo, Monica R.Zünkler, Bernd JoachimLoizzo, Monica RosaZuo, RanLojkowska, EwaZuorro, AntonioLok, Chun NamŽupanič, AnžeLoll, BernhardZupko, IstvanLollo, GiovannaŽuvela, PetarLoman, AbdullahZwergel, ClemensLombardi, Vincent C.Zwierzchowski, GrzegorzLomberget, ThierryZygmunt, MagorzataLomonaco, TommasoZygmunt, MałgorzataLongo, PasqualeŻyłła, RenataLongo, ValentinaŻyżelewicz, DorotaLoniewski, Igor


